# Chronic stress–induced ANPEP drives liver cancer progression by increasing glutathione synthesis and inhibiting ferroptosis

**DOI:** 10.1172/JCI195685

**Published:** 2025-12-02

**Authors:** Yongkang Wu, Yankun Zhang, Xiaojia Shi, Mengting Wu, Min Sun, Ying Feng, Wenmeng Ma, Xiule Jiang, Dingqi Fei, Mingjian Zhao, Zhuanchang Wu, Chunyang Li, Xiaohong Liang, Lifen Gao, Chunhong Ma, Xuetian Yue

**Affiliations:** 1 Department of Cellular Biology, School of Basic Medical Sciences;; 2 Key Laboratory for Experimental Teratology of Ministry of Education and Department of Immunology, School of Basic Medical Sciences; and; 3 Key Laboratory for Experimental Teratology of the Ministry of Education, Department of Histology and Embryology, School of Basic Medical Sciences, Cheeloo Medical College, Shandong University, Jinan, China.

**Keywords:** Cell biology, Gastroenterology, Hepatology, Apoptosis, Liver cancer, Transcription

## Abstract

Emerging evidence demonstrates that chronic stress alters immunological, neurochemical, and endocrinological functions, thereby promoting tumor progression. However, the underlying metabolic mechanism of chronic stress in tumor progression is still elusive. Using multiomics analysis, we found that aminopeptidase N (ANPEP) was upregulated in tumors with chronic restraint, associating with the reprogramming of amino acid metabolism. Functional assays revealed that ANPEP promoted liver cancer growth and metastasis. Knockdown of ANPEP blocked chronic stress–induced liver cancer progression. Chronic stress–induced glucocorticoids promoted nuclear receptor subfamily 3 group C member 1 nuclear translocation to activate ANPEP transcription by directly binding to its promoter. Furthermore, ANPEP promotes glutathione synthesis, subsequently inhibiting ROS-induced ferroptosis. Mechanistically, ANPEP interacted with solute carrier family 3 member 2 (SLC3A2) to block membrane associated ring-CH-type finger 8–mediated lysosome-dependent degradation of SLC3A2, promoting intracellular l-cystine transport, thereby increasing glutathione synthesis. The combination of ANPEP silencing and sorafenib treatment showed a synergistic effect in inhibiting liver cancer progression. Finally, clinical data and mouse models demonstrated that chronic stress drove liver tumor progression via ANPEP-regulated SLC3A2. These findings reveal unanticipated communication between chronic stress and metabolic reprogramming during liver cancer progression, providing potential therapeutic implications for liver cancer.

## Abstract 2

Wu et al showed that chronic stress promotes APN expression to increase GSH synthesis and inhibit ferroptosis in liver cancer.

## Graphical abstract

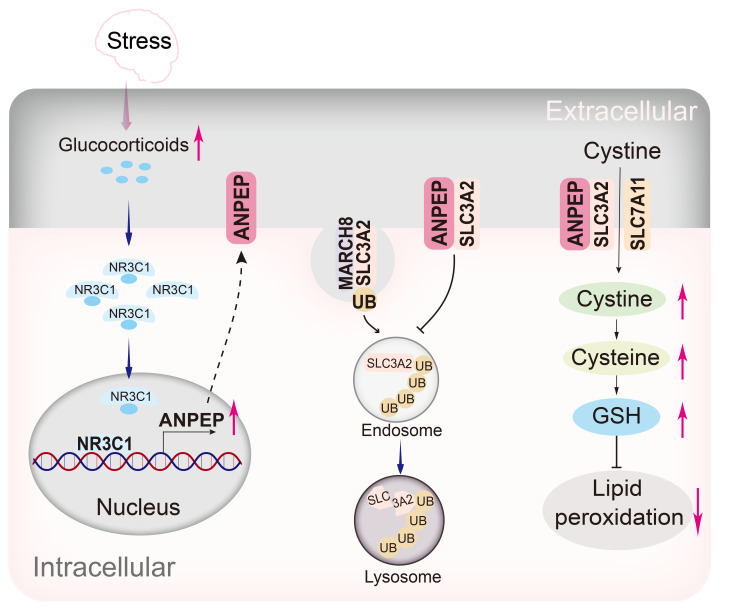

## Introduction

Mental stress, including depression, anger, fear, and so on, is naturally experienced in individuals. Emerging evidence has revealed that chronic stress is an associated risk factor for headaches, cardiovascular diseases, and even cancers (1–3). Preclinical studies using chronic restraint or social isolation in mice, 2 well-established chronic stress models, also support the notion that chronic stress promotes cancer initiation and progression (4–6). Stress-induced hormones and subsequent signals have been shown to promote cancer cell growth, angiogenesis, metastasis, and immunosuppression (4, 5, 7, 8). Glucocorticoids (GCs), important stress hormones dramatically elevated in mice with chronic restraint, play critical roles in promoting cancer progression (5, 6, 9, 10). A large meta-analysis demonstrated that chronic stress is epidemiologically linked to cancer-related mortality (in particular colorectal cancer, prostate cancer, leukemia, etc.) (11). However, the impact of chronic stress on liver cancer remains poorly understood. The underlying molecular mechanism still needs to be elucidated.

Chronic stress influences the onset, progression, and mortality of cancers by modulating hallmarks of cancer, including immune evasion, angiogenesis, sustained proliferation, metastasis, and metabolic reprogramming (4–6). Glutathione (GSH) metabolism, one of the reprogrammed cancer metabolic hallmarks, plays a critical role in cancer initiation, progression, and treatment resistance (12–16). GSH, the most abundant tripeptide (l-γ-glutamyl-l-cysteinyl-glycine), is elevated in cells and the tumor microenvironment (17, 18). GSH synthesis, depending on activity of enzymes and concentration of substrates, is a 2-step, ATP-dependent enzymatic reaction. Therefore, glutamate cysteine ligase (GCL) expression and activity, along with l-cysteine abundance, constitute rate-limiting steps for GSH synthesis (12, 19). Previous studies have reported a noticeable increase in the expression of GSH synthesis enzymes (GCLM, GCLC, and GS) and l-cysteine transporters (ASCT1/2, solute carrier family 3 member 2 [SLC3A2], and SLC7A11) in various types of cancers (12–14). GSH contributes to maintaining cellular redox homeostasis, which limits the potential of tumor cell survival and growth under majority conditions (12, 20). GSH neutralizes reactive oxygen species (ROS) and prevents cell death, particularly an iron-dependent lipid peroxidation–induced cell death, ferroptosis (21, 22). However, the interplay between chronic stress and GSH metabolism in liver cancer progression has not been well studied.

In this study, using multiomics analysis, we identified that aminopeptidase N (ANPEP, also known as CD13) was upregulated in tumors from mice subjected to chronic restraint. The prognostic significance of ANPEP expression and activity has been reported in various types of cancer, including liver cancer (23, 24). High expression of ANPEP has been strongly linked to cancer hallmarks, such as sustaining proliferative signaling, inducing or accessing vasculature, and activating invasion and metastasis (24–26). Although clinical research has suggested ANPEP as a diagnostic and prognostic marker of liver cancer, the underlying molecular mechanism of ANPEP in liver cancer progression remains unexplored. Our results demonstrated that elevated expression of ANPEP was associated with poor prognosis in patients with liver cancer. Functional experiments confirmed the oncogenic role of ANPEP in liver cancer. Interestingly, chronic stress–induced GCs promoted ANPEP transcription via nuclear receptor subfamily 3 group C member 1 (NR3C1). Further studies revealed that ANPEP stabilizes SLC3A2 at the plasma membrane by blocking its lysosome-dependent degradation mediated by membrane associated ring-CH-type finger 8 (MARCH8). This stabilization enhances intracellular transport of l-cystine and subsequent GSH synthesis, collectively suppressing ferroptosis. Targeting ANPEP combined with sorafenib treatment exhibited a greater tumor-inhibitory effect than each individual operation. Clinical data and in vivo mouse models confirmed that SLC3A2, stabilized by ANPEP, plays a pivotal role in liver cancer progression under chronic stress. These findings provide compelling evidence for possible mechanistic links between psychological stress and metabolic reprogramming during liver cancer progression.

## Results

### Chronic stress reprograms amino acid metabolism associated with ANPEP upregulation in liver cancer.

**Figure 1:**
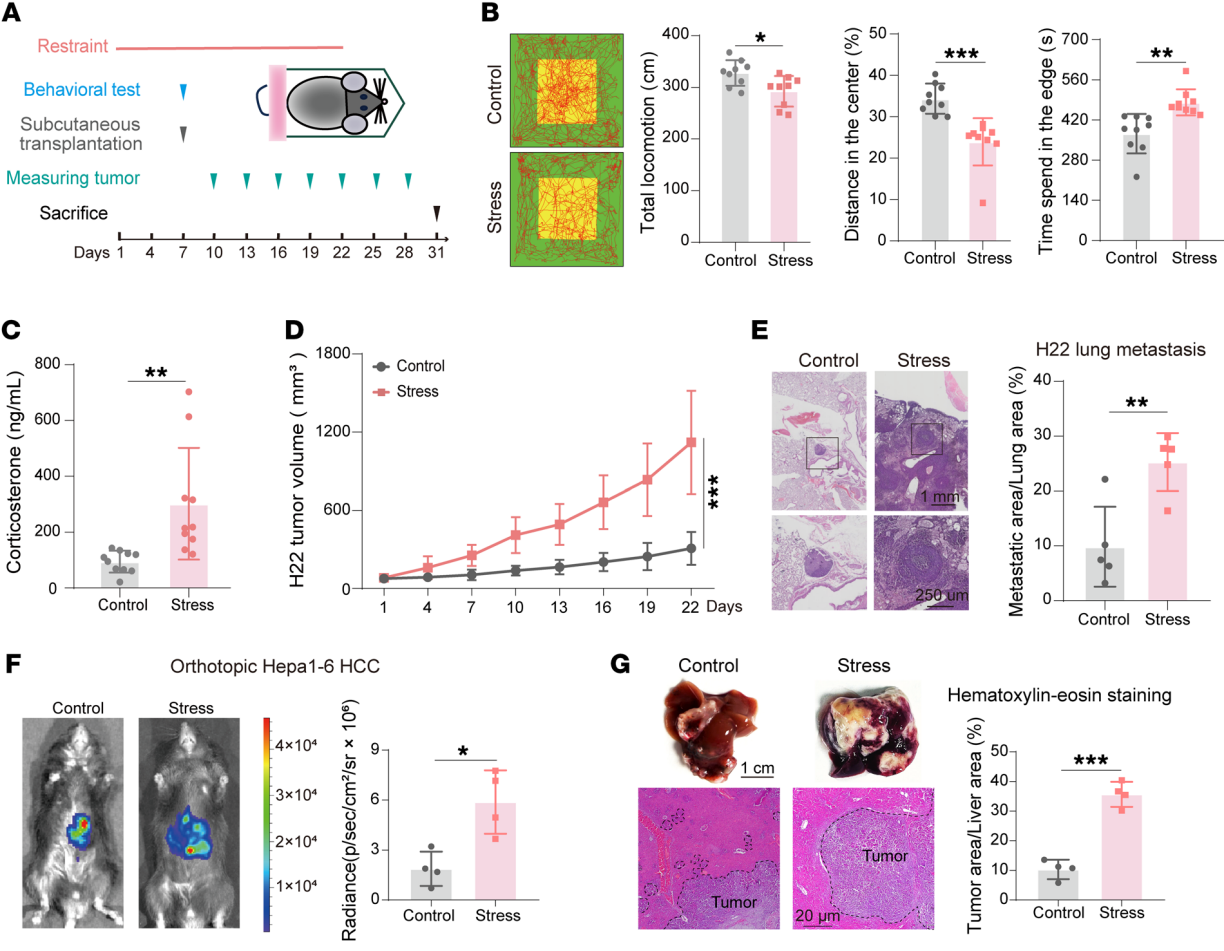
Chronic stress promotes the progression of liver cancer. (**A**–**D**) Primary H22 allograft tumors in control and stressed mice. Schematic diagram of the workflow (**A**); representative locomotion tracks and quantification of track length, center distance (%), and edge time (s) of open field test (*n* = 9) (**B**); serum corticosterone levels (*n* = 10) (**C**); and growth curves of tumors (*n* = 10) (**D**). (**E**) Lung metastasis burden in control and stress-preconditioned mice after tail vein injection of H22 cells, assessed as representative H&E images and quantification of metastatic area/lung area (%) (*n* = 5). (**F** and **G**) Orthotopic Hepa1-6–luciferase tumors in control and stressed mice after 3 weeks’ inoculation. Presented as representative bioluminescence imaging and quantification of radiance (*n* = 4) (**F**), representative liver and H&E images, and quantification of tumor area/liver area (%) (*n* = 4) (**G**). Data are presented as mean ± SD. Significance was assessed by 2-way ANOVA with Šidák’s multiple comparisons test (**D**) or 2-tailed unpaired Student’s *t* test (**B**, **C**, and **E**–**G**). *P < 0.05; **P < 0.01; ***P < 0.001. HCC, hepatocellular carcinoma.

**Figure 2:**
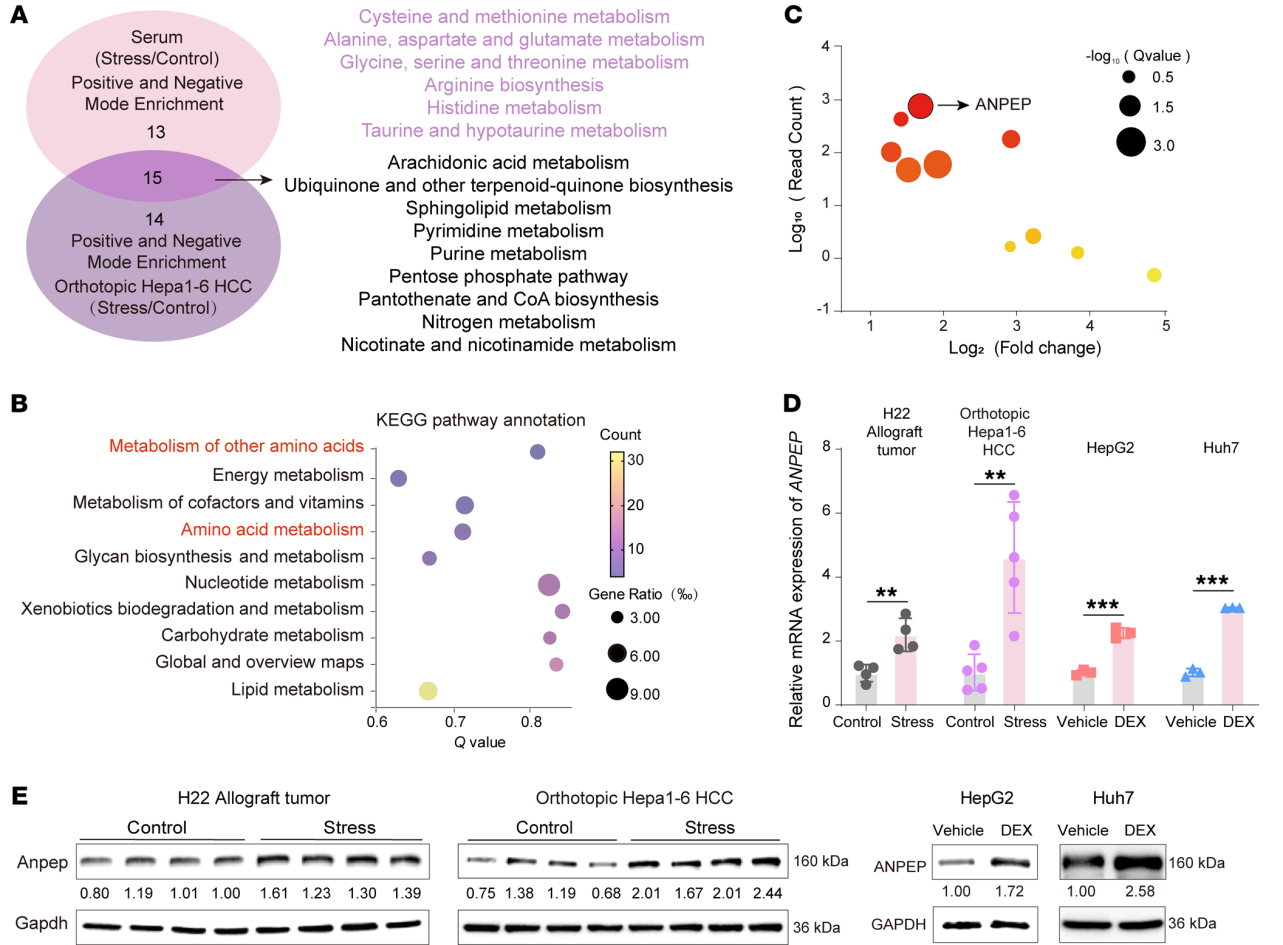
Chronic stress reprograms amino acid metabolism and upregulates ANPEP expression in liver cancer. (**A**) Venn diagram displayed intersecting pathway enrichment analysis of differential metabolites in serum of mice with H22 allograft tumor (*n* = 3) and in liver of mice with orthotopic Hepa1-6 tumor (*n* = 5). (**B**) The bubble chart of stress-upregulated genes in metabolic signaling pathways, as identified by KEGG pathway analysis. (**C**) The bubble plot of stress-upregulated genes in amino acid–associated pathways. (**D** and **E**) ANPEP mRNA and protein levels in primary H22 (*n* = 4) and Hepa1-6 tumors from control and stressed mice (*n* = 5) and in Huh7/HepG2 cells after dexamethasone (DEX; 1 μM) for 48 hours (*n* = 3). Data are presented as mean ± SD. Significance was assessed by 2-tailed unpaired Student’s *t* test (**D**). **P < 0.01; ***P < 0.001.

To examine the impact of chronic stress upon tumorigenesis in vivo, a chronic restraint mouse model was used (Figure 1A). Chronic restraint caused anxiety-like behavior, which manifested as diminished total locomotion and exploration in the central area of the open field test (Figure 1B). Mouse body weights were reduced in stressed mice (Supplemental Figure 1A; supplemental material available online with this article; https://doi.org/10.1172/JCI195685DS1). Serum corticosterone levels, TG levels, and free fatty acid levels were elevated, whereas glucose levels remained unchanged in mice subjected to chronic restraint (Figure 1C and Supplemental Figure 1, B and C). As expected, chronic restraint promoted liver cancer progression, displaying as higher tumor growth rate and lung metastatic ability in mice with chronic restraint than those in control mice (Figure 1, D and E). Similarly, chronic restraint promoted orthotopic liver tumor growth (Figure 1, F and G). To investigate the systemic metabolic changes, the sera of allograft tumor models and orthotopic liver tumor models were collected for metabolomics (Supplemental Tables 1–4). The principal component analysis (PCA) showed that the metabolites from both models were well separated in chronic restraint and control groups (Supplemental Figure 1, D and E). The metabolite set enrichment analysis (MSEA) revealed that many amino acid metabolic pathways, such as cysteine and methionine metabolism; alanine, aspartate, and glutamate metabolism; and arginine biosynthesis, were commonly enriched in both models with chronic restraint (Figure 2A and Supplemental Table 5). These data suggest that chronic restraint promotes liver cancer progression associated with systemic amino acid metabolic reprogramming.

To identify a key regulator involved in amino acid metabolic reprogramming in liver cancer with chronic stress, the allograft tumor samples were collected for RNA sequencing (RNA-Seq). RNA-Seq identified a total of 889 differentially expressed genes (DEGs) (|log_2_FC| ≥ 1.00, adjusted *P* ≤ 0.05) (Supplemental Figure 1F and Supplemental Table 6). Kyoto Encyclopedia of Genes and Genomes (KEGG) enrichment analysis of upregulated DEGs showed that amino acid metabolism–related pathways were among the most substantially altered metabolic pathways in tumors from mice exposed to chronic stress (Figure 2B). Further analysis demonstrated that *ANPEP*, a gene related to amino acid metabolism, was the most strongly upregulated gene in tumors from mice subjected to chronic restraint (Figure 2C). The elevated ANPEP mRNA and protein levels were confirmed in mouse tumors with chronic restraint (Figure 2, D and E). Dexamethasone (DEX), a synthetic glucocorticoid receptor (GR) agonist, upregulated ANPEP expression in both HepG2 and Huh7 cells (Figure 2, D and E). Furthermore, analysis of publicly available single-cell datasets of liver cancer showed that high *ANPEP* mRNA levels in malignant cell populations were observed in 3 of 4 datasets (Supplemental Figure 1G) (http://tisch.compbio.cn). Notably, ANPEP upregulation can also be induced in colon cancer by chronic stress (Supplemental Figure 1, H–J). Therefore, ANPEP, regulating amino acid metabolism, is elevated in tumors induced by chronic stress.

### Chronic stress drives liver cancer progression via ANPEP.

**Figure 3:**
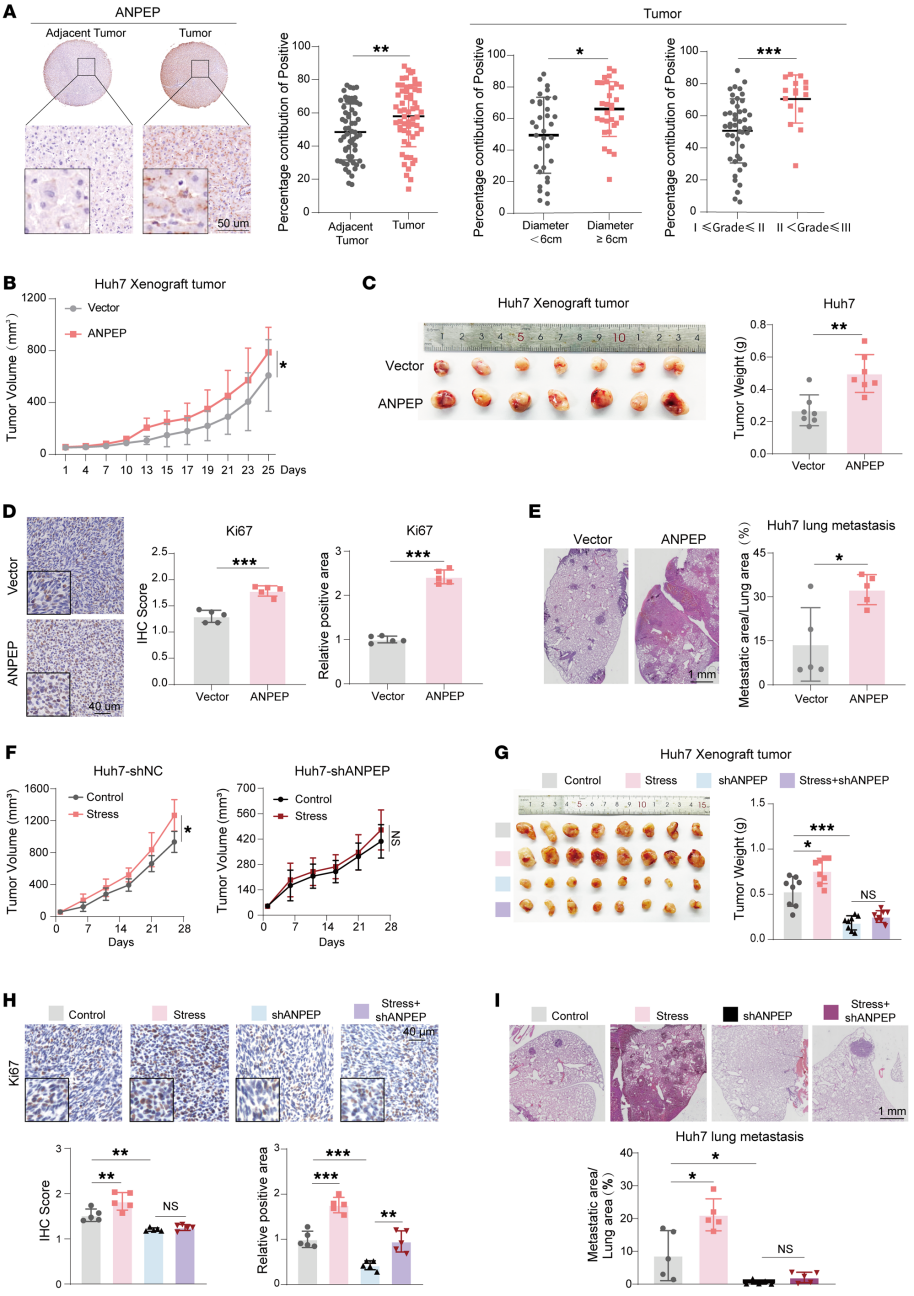
ANPEP mediates chronic stress–induced liver cancer progression. (**A**) Representative immunohistochemical images of tumor tissues and adjacent nontumor tissues stained with anti-ANPEP antibody in patients with HCC. ANPEP-positive staining was quantified using ImageJ (NIH) and statistically analyzed (*n* = 60 per group). Subgroup analyses based on tumor diameter (<6 cm, *n* = 34; ≥6 cm, *n* = 30) and pathological grade (I ≤ grade ≤ II, *n* = 47; II < grade ≤ III, *n* = 16). (**B**–**D**) Xenograft models established with Huh7-Vector or Huh7-ANPEP cells. Tumor growth curves (*n* = 10) (**B**), tumor images and weights (*n* = 7) (**C**), and Ki67-positive cells (*n* = 5) (**D**) are shown. (**E**) Lung metastasis models established by tail vein injection of Huh7-Vector or Huh7-ANPEP cells. Representative H&E-stained lung sections and quantification of metastatic area are shown (*n* = 5). (**F**–**I**) Huh7 control shRNA (Huh7-shNC) and Huh7-shANPEP xenograft tumors and lung metastatic nodes in nude mice with or without restraint stress. Tumor growth (*n* = 9) (**F**), tumor images and weights (*n* = 8) (**G**), Ki67 staining (*n* = 5) (**H**), and lung metastases (*n* = 5) (**I**) were analyzed. Data are presented as mean ± SD. Statistical significance was determined by 2-way ANOVA with Šidák’s multiple comparisons test (**B** and **F**), 1-way ANOVA with Tukey’s multiple comparisons test (**G**–**I**), and 2-tailed unpaired Student’s *t* test (**A** and **C**–**E**). *P < 0.05; **P < 0.01; ***P < 0.001.

To explore the role of ANPEP in liver cancer, we analyzed the clinical relevance of ANPEP using patient-derived liver tumor samples. As shown in Figure 3A, the protein levels of ANPEP were higher in liver cancer tissues than those in adjacent nontumor tissues. High expression of ANPEP was positively associated with tumor size and tumor grade. We got similar results by analyzing publicly available liver cancer datasets (TCGA and GTEx, GSE1898, and GSE39791) (Supplemental Figure 2A) (27, 28). High level of ANPEP was associated with a poor prognosis of patients with liver cancer (Supplemental Figure 2B). Subsequently, we established liver cancer cells with stable overexpression or knockdown of ANPEP (Supplemental Figure 2, C and D). In vitro functional assays showed that ANPEP overexpression promoted proliferation and migration of liver cancer cells, whereas knockdown of ANPEP inhibited these functions (Supplemental Figure 2, E–H). Transfection of shRNA-resistant ANPEP reversed the inhibitory effect of ANPEP knockdown in cell proliferation and migration (Supplemental Figure 2, F and H). Further in vivo experiments demonstrated that overexpression of ANPEP promoted xenograft tumor progression and lung metastasis, displaying as increased tumor growth, tumor weight, Ki67-positive cells, and lung tumor areas (Figure 3, B–E). Together, these results suggest that ANPEP is an oncogene in liver cancer.

To examine the role of ANPEP in chronic stress driving liver cancer development, we subcutaneously injected chronic restraint nude mice with ANPEP-silenced or control Huh7 cells. As shown in Figure 3F, chronic stress promoted xenograft tumor growth in mice injected with control Huh7 cells. Notably, silencing of ANPEP almost completely abolished chronic stress–induced increase in tumor growth. Similarly, chronic stress–induced increases in mouse tumor weight and Ki67-positive cell numbers were also eliminated by ANPEP knockdown (Figure 3, G and H). In addition, knockdown of ANPEP clearly abolished chronic stress–induced increase in lung metastasis (Figure 3I). Consistently, in vitro Transwell assay verified that knockdown of ANPEP abolished DEX-induced increase in migrated cell numbers (Supplemental Figure 2I). All these data demonstrate that chronic stress drives liver cancer progression via ANPEP.

### Chronic stress induces NR3C1 nuclear translocation to promote ANPEP transcription.

**Figure 4:**
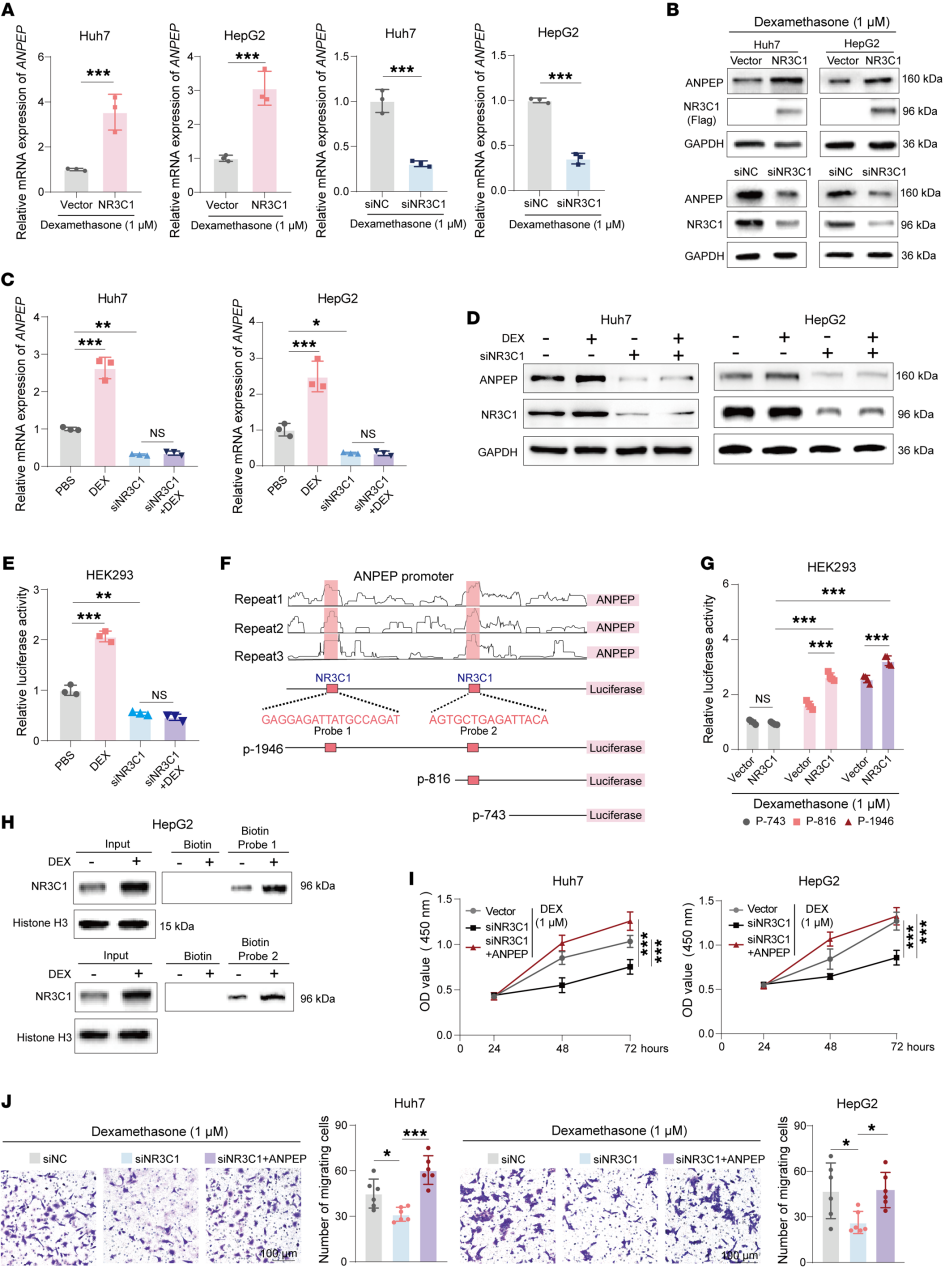
Chronic stress promotes ANPEP transcription through NR3C1 activation. (**A** and **B**) ANPEP mRNA and protein levels in Huh7 and HepG2 cells with indicated manipulations after 48 hours of DEX treatment (**A**, *n* = 3). (**C** and **D**) ANPEP mRNA and protein levels in control and NR3C1-knockdown Huh7 and HepG2 cells after 48 hours of DEX treatment (**C**, *n* = 3). (**E**) ANPEP promoter activity in HEK293 with or without NR3C1 knockdown after DEX treatment (*n* = 3). (**F**) A schematic of NR3C1 binding regions on the ANPEP promoter based on the ENCODE database. Corresponding truncations of the ANPEP promoter were constructed, and biotin-labeled DNA probes were designed. (**G**) Luciferase activity of ANPEP promoter truncations in HEK293 cells with or without NR3C1 overexpression, followed by 48 hours of DEX treatment (*n* = 3). (**H**) Biotin-labeled DNA probes were incubated with nuclear extracts from HepG2 cells with or without DEX treatment, followed by streptavidin pull-down and Western blotting. (**I** and **J**) Cell proliferation (**I**) and Transwell assays (**J**) in Huh7 and HepG2 cells with indicated manipulations, after 48 hours of DEX treatment (**I**, *n* = 5; **J**, *n* = 6). Data are presented as mean ± SD. Statistical significance was determined by 2-way ANOVA with Šidák’s multiple comparisons test (**I**), 1-way ANOVA with Tukey’s multiple comparisons test (**C**, **E**, and **J**) and 2-tailed unpaired Student’s *t* test (**A** and **G**). *P < 0.05; **P < 0.01; ***P < 0.001.

The stress hormone cortisol and synthetic glucocorticoids transcriptionally activate target genes via the GR, encoded by *NR3C1* (29). To determine whether glucocorticoids upregulate ANPEP transcription via NR3C1, we manipulated NR3C1 expression in liver cancer cells with DEX treatment (Supplemental Figure 3A). As shown in Figure 4A, overexpression of NR3C1 upregulated *ANPEP* mRNA levels, whereas knockdown of NR3C1 downregulated *ANPEP* mRNA levels in liver cancer cells with DEX treatment. Consequently, the protein levels of ANPEP were positively correlated with NR3C1 levels (Figure 4B). Furthermore, DEX treatment induced NR3C1 nuclear translocation (Supplemental Figure 3B). DEX-induced increase in ANPEP expression was abolished by NR3C1 knockdown (Figure 4, C and D). These data indicate that chronic stress promotes ANPEP transcription via NR3C1.

To decipher the mechanism by which NR3C1 regulates ANPEP transcription, the luciferase reporter containing ANPEP promoter was constructed. DEX treatment increased luciferase activity of ANPEP promoter (Supplemental Figure 3C). Similarly, overexpression of NR3C1 enhanced ANPEP promoter activity, whereas NR3C1 knockdown reduced it in HEK293 cells with DEX treatment (Supplemental Figure 3D). Notably, knockdown of NR3C1 completely abolished DEX-induced increase in ANPEP promoter activity (Figure 4E). Furthermore, we identified 2 putative NR3C1 binding sites on ANPEP promoter by analyzing a ChIP-Seq dataset obtained from ENCODE (GSE170210) (30). A series of ANPEP promoter luciferase reporters were constructed according to the location of NR3C1 binding sites (Figure 4F). As shown in Figure 4G and Supplemental Figure 3E, deletion of the 2 NR3C1 binding sites abolished the reporter activation induced by NR3C1 overexpression or DEX treatment, respectively. Further pull-down assay demonstrated that biotin-labeled probes specifically interacted with nuclear NR3C1, which was extracted from HepG2 cells with or without DEX treatment (Figure 4H). In vitro function assays found that knockdown of NR3C1 inhibited cell migration and proliferation. Overexpression of ANPEP rescued the effect of NR3C1 knockdown in inhibiting cell migration and proliferation (Figure 4, I and J, and Supplemental Figure 3F). Therefore, glucocorticoids promote ANPEP transcription through direct binding of NR3C1 to its promoter.

### ANPEP enhances GSH synthesis to inhibit ferroptosis in liver cancer.

**Figure 5:**
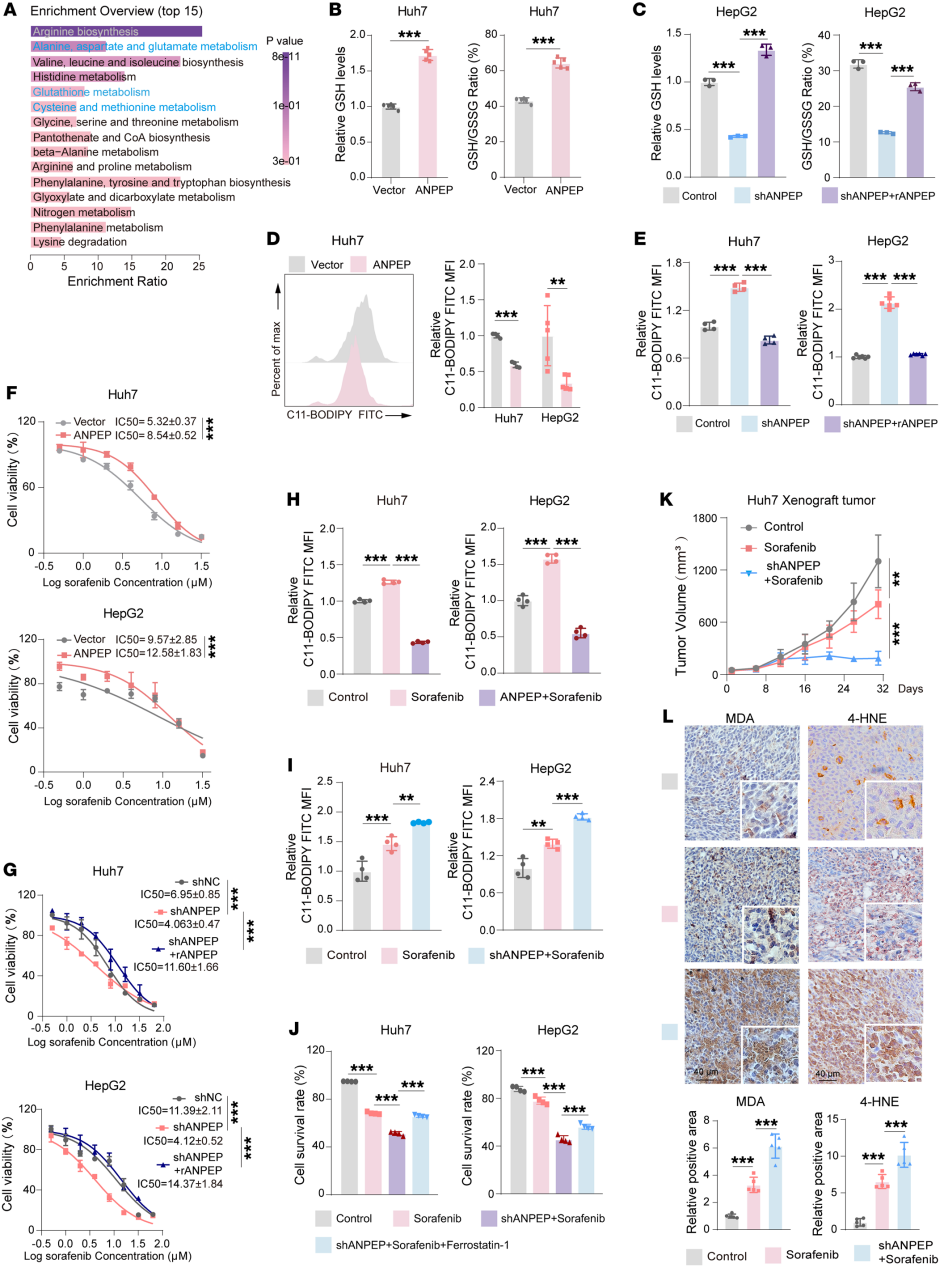
ANPEP enhances GSH levels to reduce sorafenib sensitivity of liver cancer. (**A**) Targeted amino acid metabolomic profiling of Huh7 cells with or without ANPEP overexpression, followed by KEGG pathway enrichment analysis of differential metabolites. (**B** and **C**) GSH levels and GSH/GSSG ratios in liver cancer cells with ANPEP manipulation (**B**, *n* = 5; **C**, *n* = 3). (**D** and **E**) Lipid peroxide levels were labeled by C11-BODIPY in Huh7 and HepG2 cells with ANPEP manipulation (**D**: Huh7, *n* = 4; HepG2, *n* = 5. **E**: Huh7, *n* = 4; HepG2, *n* = 6). (**F** and **G**) IC_50_ of ANPEP-manipulated Huh7 and HepG2 cells with 24 hours of sorafenib treatment (0, 1, 2, 4, 8, 16, 32, 64 μM) (*n* = 3). (**H** and **I**) Lipid peroxides in liver cancer cells treated with sorafenib alone or sorafenib plus ANPEP manipulation, quantified by C11-BODIPY intensity (*n* = 4). (**J**) Cell viability of Huh7 and HepG2 cells stably expressing shNC or shANPEP after 24 hours of treatment with sorafenib (10 μM) and/or ferrostatin-1 (2 μM) (*n* = 4). (**K** and **L**) Xenograft tumor model established by subcutaneous injection of ANPEP-manipulated Huh7 cells into nude mice, followed by treatment with sorafenib or vehicle. Tumor growth (**K**) and MDA and 4-HNE levels (**L**) were assessed (**K**, *n* = 8; **L**, *n* = 5). Data are presented as mean ± SD. Statistical significance was determined by 2-way ANOVA with Šidák’s multiple comparisons test (**K**), 1-way ANOVA with Tukey’s multiple comparisons test (**C**, **E**, **H**–**J**, and **L**), and 2-tailed unpaired Student’s *t* test (**B**, **D**, **F**, and **G**). **P < 0.01; ***P < 0.001.

To further explore how ANPEP regulates amino acid metabolism, targeted amino acid metabolomics was performed in Huh7 cells with ANPEP manipulation. The PCA revealed a clear separation in metabolite profiles between vector- and ANPEP-transfected Huh7 cells (Supplemental Figure 4A). The MSEA demonstrated that glutathione metabolism, and its related metabolic pathways including glutamate metabolism and cysteine metabolism, were among the top changed metabolic pathways (Figure 5A). GSH/GSSG is the main redox pair that determines the antioxidative capacity of cells (12, 20). Here, overexpression of ANPEP increased GSH levels and GSH/GSSG ratio in Huh7 cells (Figure 5B). Knockdown of ANPEP decreased GSH levels and GSH/GSSG ratio, which were reversed by transfection of shRNA-resistant ANPEP in HepG2 cells (Figure 5C). Subsequently, ANPEP levels were negatively associated with total ROS levels in liver cancer cells (Supplemental Figure 4, B and C). Interestingly, ANPEP overexpression reduced ROS-induced lipid peroxidation (Figure 5D), whereas ANPEP knockdown enhanced lipid peroxidation, which was reversed by shRNA-resistant ANPEP (Figure 5E). In addition, silencing ANPEP reduced GSH levels and GSH/GSSG ratio, and increased total ROS levels and lipid peroxidation in HepG2 cells, with or without DEX treatment (Supplemental Figure 4, D and E). Collectively, ANPEP upregulates GSH levels to reduce ROS levels and lipid peroxidation.

Sorafenib is the first FDA-approved drug for liver cancer treatment, which induces lipid peroxidation–dependent ferroptosis (31–33). Therefore, the synergistic therapeutic effect of ANPEP knockdown and sorafenib combination was tested. As shown in Figure 5, F and G, overexpression of ANPEP decreased the sensitivity of liver cancer cells to sorafenib, while knockdown of ANPEP increased the sensitivity of liver cancer cells, which was reversed by transfection of shRNA-resistant ANPEP. Further examinations demonstrated that ANPEP overexpression attenuated sorafenib-induced ROS levels and lipid peroxidation increasing, whereas ANPEP knockdown enhanced them (Figure 5, H and I, and Supplemental Figure 4, F and G). Importantly, silencing ANPEP increased sorafenib cell-killing effects, which were reversed by treating with ferroptosis inhibitor ferrostatin-1 (Figure 5J), indicating ANPEP blocks sorafenib-induced ferroptosis. In addition, ANPEP regulated GSH metabolism, redox balance, and lipid peroxidation depending on its aminopeptidase activity (Supplemental Figure 4, H and I). Furthermore, a xenograft tumor model showed that ANPEP silencing enhanced the tumor-inhibitory effect of sorafenib. This was evidenced by greater reduction in tumor growth, tumor weight, and number of Ki67-positive cells in the combination group than sorafenib treatment alone (Figure 5K and Supplemental Figure 4, J and K). Notably, the sorafenib-induced increase in 4-hydroxynonenal (4-HNE) and malondialdehyde (MDA), lipid peroxidation markers detected by IHC, was more pronounced in ANPEP-silenced tumors than with sorafenib treatment alone (Figure 5L), indicating that the combination induces higher lipid peroxidation.

### ANPEP promotes GSH synthesis via SLC3A2-mediated intracellular transport of l-cystine.

**Figure 6:**
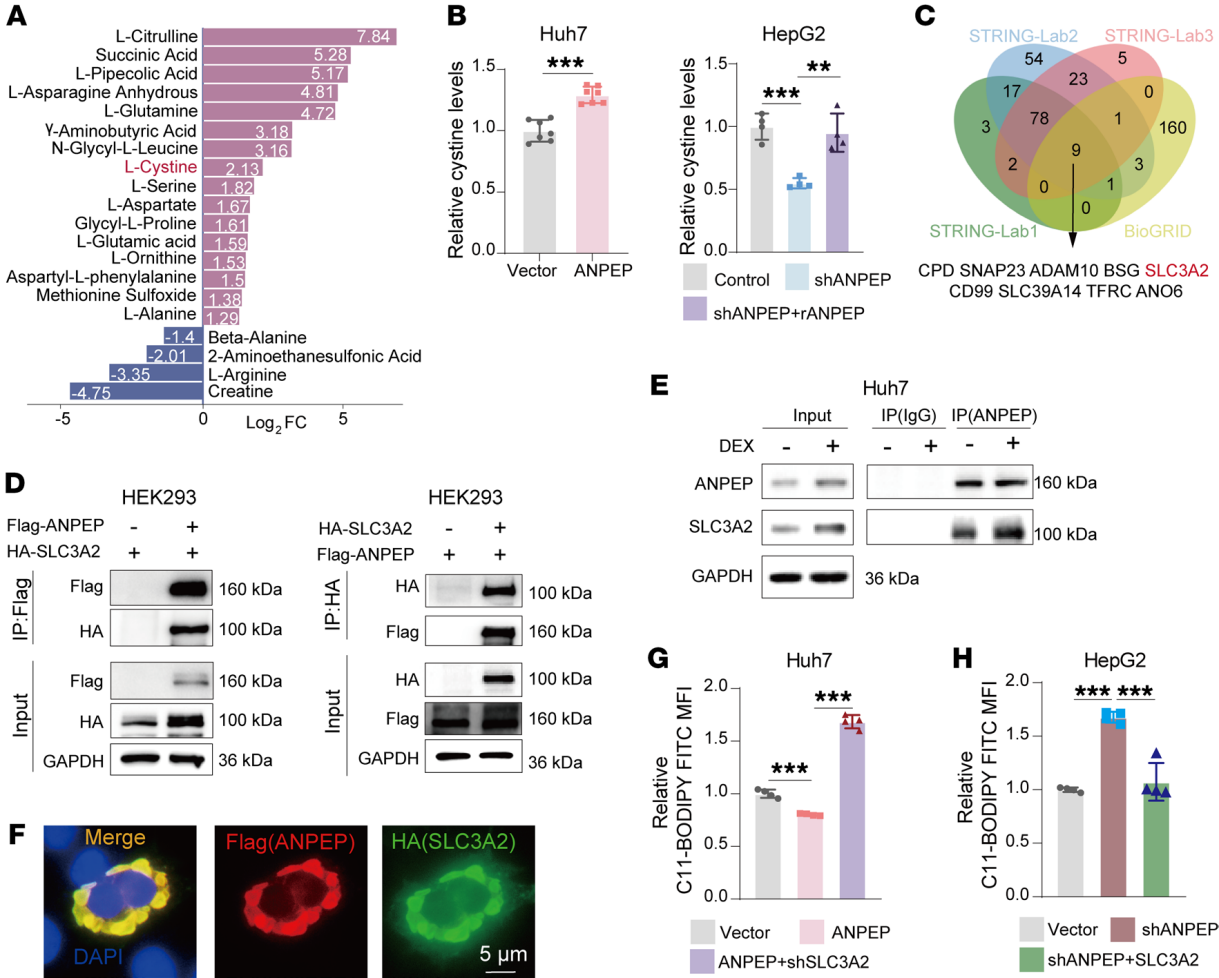
ANPEP interacts with SLC3A2 to upregulate intracellular l-cystine and inhibit lipid peroxidation. (**A**) The top fold-change (FC) metabolites between vector- and ANPEP-overexpressed Huh7 cells were identified through targeted amino acid metabolomic analysis. (**B**) l-cystine levels were examined in liver cancer cells with ANPEP manipulation (Huh7, *n* = 7; HepG2, *n* = 4). (**C**) The Venn diagram shows potential ANPEP-interacting proteins based on online databases (STRING and BioGRID). (**D**–**F**) ANPEP-SLC3A2 interactions were analyzed by co-IP/Western blotting in HEK293 (**D**) and Huh7 cells (**E**) and immunofluorescence in HEK293 (**F**). (**G** and **H**) C11-BODIPY fluorescence was measured in liver cancer cells with indicated manipulations (*n* = 4). Data are presented as mean ± SD. Statistical significance was determined by 1-way ANOVA with Tukey’s multiple comparisons test (**B**, **G**, and **H**). **P < 0.01; ***P < 0.001.

To identify the downstream targets responsible for ANPEP-mediated elevation of GSH levels, differential metabolite analysis was performed. As shown in Figure 6A, the levels of l-cystine were strongly increased in ANPEP-overexpressed Huh7 cells. Consistently, intracellular l-cystine levels were positively associated with ANPEP expression in liver cancer cells (Figure 6B). In addition, overexpression of ANPEP increased levels of l-glutamine and l-glutamic acid in Huh7 cells (Supplemental Figure 5A). l-cystine converts to l-cysteine, the precursor for GSH synthesis (19). Thus, intracellular l-cysteine levels were also positively associated with ANPEP expression (Supplemental Figure 5B). Notably, ANPEP silencing reduced l-cysteine in HepG2 cells with or without DEX treatment (Supplemental Figure 5C). Therefore, ANPEP regulates GSH precursor l-cysteine levels in liver cancer cells.

Since ANPEP is a transmembrane protein, we proposed that ANPEP might influence intracellular transport of l-cystine. SLC3A2, a known subunit of l-cystine transporter (13), was predicted as an ANPEP-interacted protein by using online tools (STRING and BioGRID) (Figure 6C). Exogenous and endogenous co-IP assays and immunofluorescence staining demonstrated that ANPEP interacted with SLC3A2 (Figure 6, D–F). Similarly, ANPEP-SLC3A2 interaction was observed in cells with DEX treatment (Figure 6E and Supplemental Figure 5D). Furthermore, co-IP assays performed with ANPEP truncations and SLC3A2 truncations showed that 66–287 aa and 636–967 aa of ANPEP exhibited interaction with SLC3A2, and SLC3A2 (205–630 aa) interacted with ANPEP (Supplemental Figure 5, E and F). In addition, ANPEP interacted with SLC7A11, forming a complex with SLC3A2 to mediate l-cystine transport into cells (Supplemental Figure 5G). This interaction is dependent on SLC3A2 (Supplemental Figure 5H). Importantly, manipulation of SLC3A2 eliminated ANPEP-mediated changes in intracellular l-cystine, GSH levels, and 2′,7′-dichlorodihydrofluorescein diacetate (DCFHDA; a cell-permeable fluorescent probe widely used to detect intracellular ROS) levels (Supplemental Figure 5I), displayed as silencing SLC3A2 abolished ANPEP overexpression–induced increase in l-cystine and GSH and decrease in DCFHDA (Supplemental Figure 5J), and overexpressing SLC3A2 rescued ANPEP knockdown–induced l-cystine and GSH reduction and DCFHDA elevation (Supplemental Figure 5K). Consequently, knockdown of SLC3A2 abolished ANPEP overexpression–induced reduction in C11-BODIPY levels in liver cancer cells (Figure 6G). Conversely, SLC3A2 overexpression rescued ANPEP knockdown–induced increase in C11-BODIPY levels in liver cancer cells (Figure 6H). All these data demonstrate that ANPEP interacts with SLC3A2 to mediate intracellular transport of l-cystine in liver cancer cells, increasing GSH levels to inhibit lipid peroxidation.

**Figure 7:**
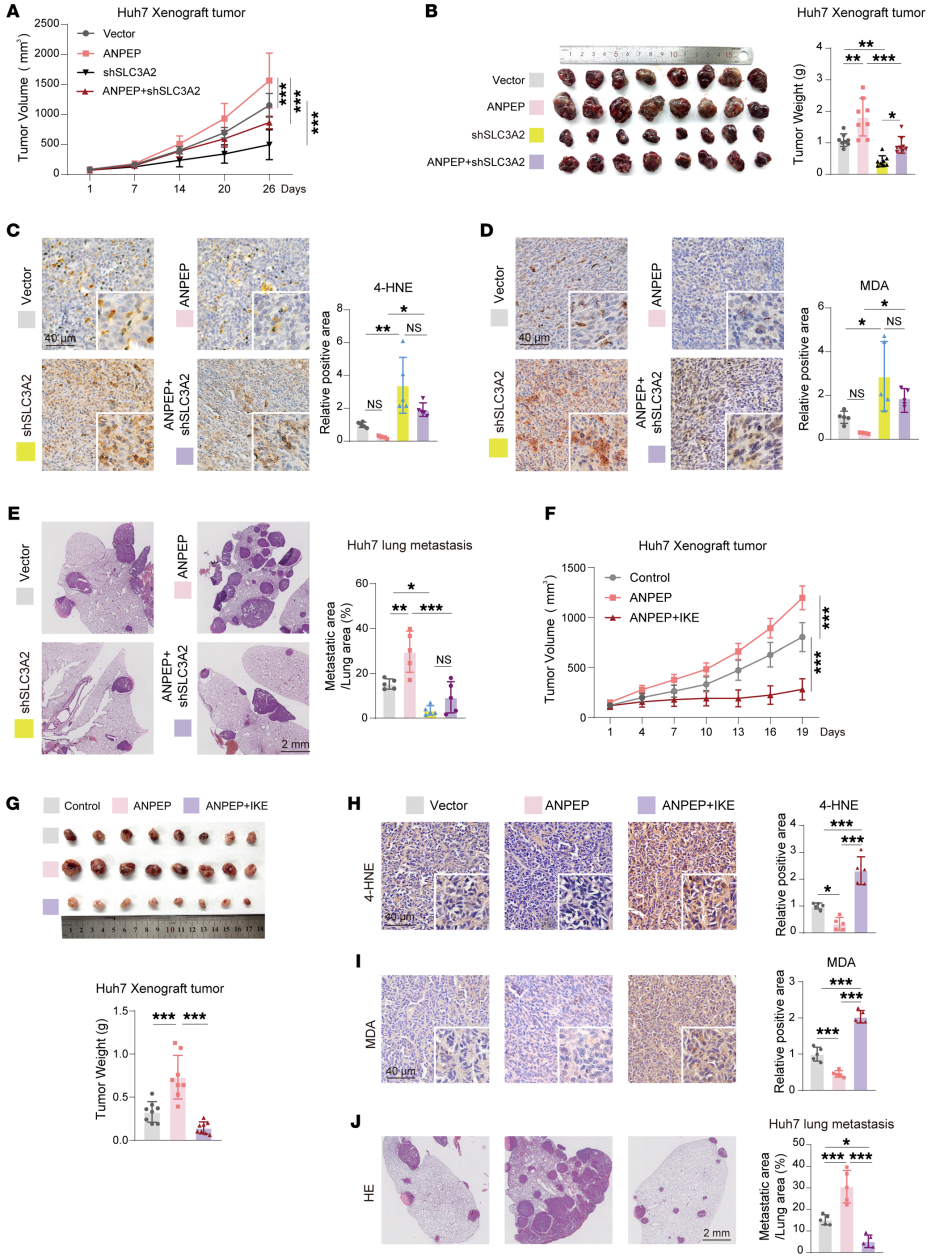
ANPEP promotes liver cancer progression via SLC3A2-mediated inhibition of lipid peroxidation–dependent ferroptosis. (**A**–**E**) Nude mice were injected with Huh7 cells with indicated manipulation either subcutaneously to establish xenograft tumors or via tail vein to establish lung metastases. Tumor growth curves (**A**), tumor images and weight (**B**), 4-HNE levels (**C**), MDA levels (**D**), and representative H&E images with quantification of lung metastases (**E**) were assessed (**A** and **B**, *n* = 8; **C**–**E**, *n* = 5). (**F**–**J**) Xenograft tumor model and lung metastatic model were established by injection of ANPEP-manipulated Huh7 subcutaneously or via tail vein, followed by treatment with IKE or vehicle. Tumor growth (**F**), tumor images and weight (**G**), 4-HNE (**H**) and MDA levels (**I**), and representative H&E images with quantification of lung metastases (**J**) were assessed (**F** and **G**, *n* = 8; **H**–**J**, *n* = 5). Data are presented as mean ± SD. Statistical significance was determined by 2-way ANOVA with Šidák’s multiple comparisons test (**A** and **F**) and 1-way ANOVA with Tukey’s multiple comparisons test (**B**–**E** and **G**–**J**). *P < 0.05; **P < 0.01; ***P < 0.001.

To explore the role of the ANPEP/SLC3A2 axis in tumor development, xenograft tumor and lung metastasis were performed using Huh7 with ANPEP and SLC3A2 manipulation. As shown in Figure 7A, ANPEP overexpression promoted xenograft tumor growth, and SLC3A2 silencing inhibited xenograft tumor growth. Notably, knockdown of SLC3A2 abolished the effect of ANPEP in promoting tumor growth. Furthermore, silencing SLC3A2 blocked ANPEP-induced increase in tumor weight and Ki67-positive cells and reduction of TUNEL-positive cells, 4-HNE levels, and MDA levels (Figure 7, B–D, and Supplemental Figure 5, L and M). Additionally, knockdown of SLC3A2 abolished ANPEP-induced increase in lung metastasis (Figure 7E). In addition, ANPEP-induced tumor growth and lung metastasis were blocked by ferroptosis inducer imidazole ketone erastin (IKE) (Figure 7, F–J, and Supplemental Figure 5, N and O). Therefore, SLC3A2 is a downstream target that regulates ANPEP-mediated liver cancer development through lipid peroxidation–induced ferroptosis.

### ANPEP promotes intracellular transport of l-cystine by stabilizing SLC3A2.

**Figure 8:**
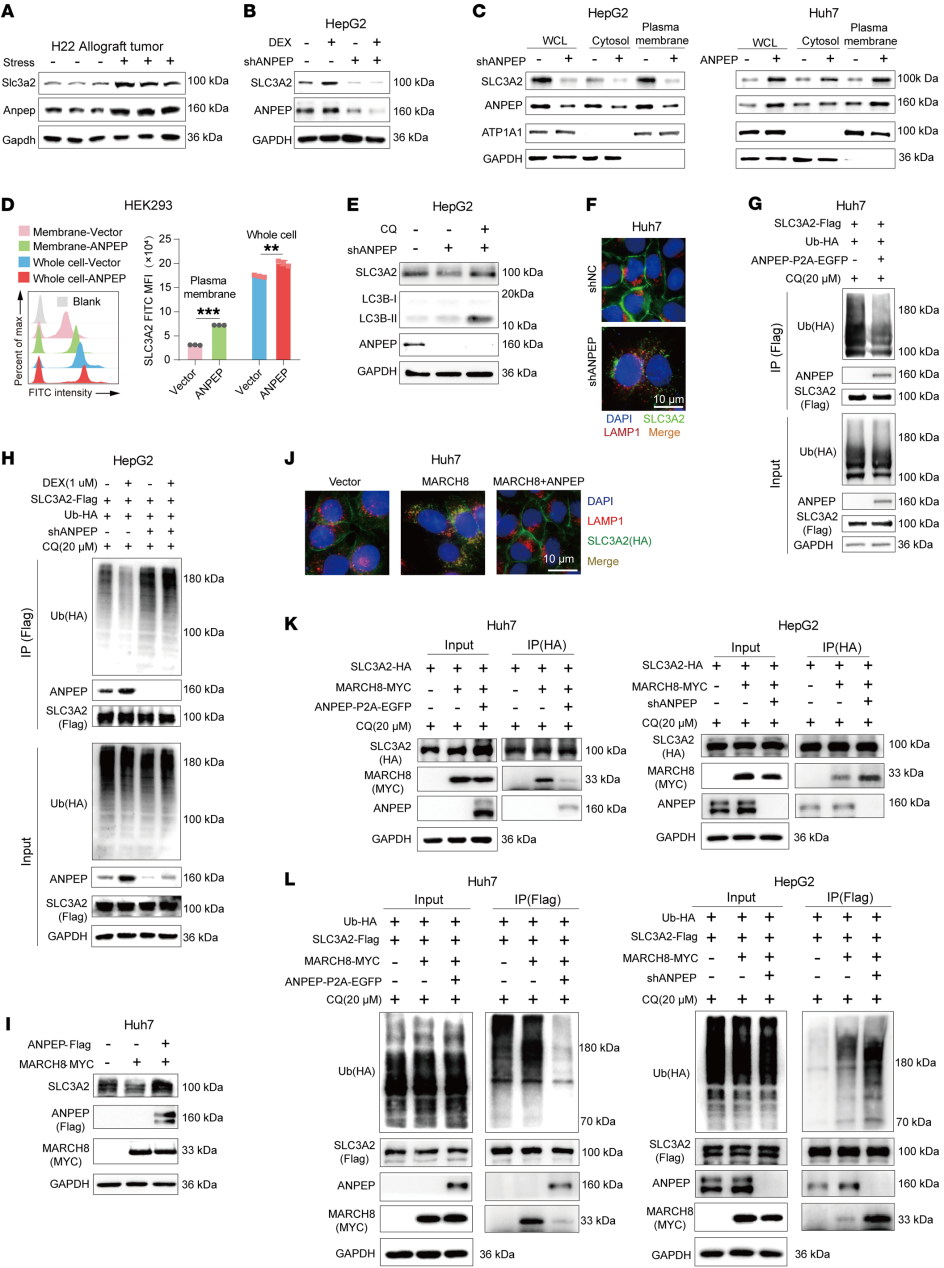
ANPEP stabilizes SLC3A2 by blocking MARCH8-mediated lysosomal dependent degradation of SLC3A2. (**A**) Protein levels of SLC3A2 and ANPEP were analyzed in H22 allograft tumors with or without stress. (**B**) Western blot analysis of SLC3A2 and ANPEP protein levels in shNC- or shANPEP-transduced HepG2 cells after 48 hours’ DEX treatment. (**C**) Immunoblots of cytoplasmic and membrane fractions extracted from the indicated liver cancer cells. (**D**) Whole-cell and membrane fluorescence intensity of SLC3A2 were examined in HEK293 cells with or without ANPEP overexpression. Representative histograms and statistical analysis were shown (*n* = 3). (**E**) Immunoblots of HepG2 cells with ANPEP manipulation and/or CQ administration. (**F**) SLC3A2 was labeled with primary antibody to trace its lysosomal trafficking in CQ-treated Huh7 cells with or without ANPEP knockdown. (**G**) Ubiquitination levels of SLC3A2 were determined in ANPEP-overexpressed Huh7 cells after CQ treatment by co-IP assay and followed by Western blotting. (**H**) Ubiquitination levels of SLC3A2 were determined in ANPEP-knockdown HepG2 cells with or without DEX treatment by IP assay and followed by Western blotting. (**I**) SLC3A2 protein levels were examined in MARCH8-transfected Huh7 cells with or without ANPEP overexpression. (**J**) SLC3A2 lysosomal trafficking was examined in MARCH8-transfected Huh7 cells with or without ANPEP overexpression. (**K**) Interaction between SLC3A2 and MARCH8 was assessed in CQ-treated liver cancer cells with or without ANPEP overexpression. (**L**) Ubiquitination levels of SLC3A2 were analyzed in liver cancer cells with or without ANPEP overexpression after CQ treatment. Data are presented as mean ± SD. Statistical significance was determined by 2-tailed unpaired Student’s *t* test (**D**). **P < 0.01; ***P < 0.001.

To decipher the underlying mechanism by which ANPEP promotes SLC3A2-mediated l-cystine intracellular transport, SLC3A2 protein levels were examined in tumor models and liver cancer cells. As shown in Figure 8A, stress induced upregulation of ANPEP and SLC3A2 in H22 allograft tumors. In HepG2 cells, DEX-induced upregulation of SLC3A2 was abolished by ANPEP knockdown (Figure 8B). Similarly, ANPEP knockdown abolished stress-induced increasing of SLC3A2 levels in Huh7 xenograft tumor (Supplemental Figure 6A). In addition, ANPEP overexpression upregulated SLC7A11 protein levels in Huh7 cells (Supplemental Figure 6B). Further examination verified that silencing of ANPEP decreased SLC3A2 protein levels, while overexpressing of ANPEP increased SLC3A2 protein levels in whole-cell lysates. Notably, these differences were also displayed in membrane fractions (Figure 8C). These findings were confirmed in flow cytometry by staining total and membrane SLC3A2, respectively (Figure 8D). Endocytosis, the late endosomal/lysosomal dependent protein degradation pathway, is the principal means by which plasma membrane proteins are degraded (34, 35). Treatment with CQ, a well-known lysosomal inhibitor, induced swelling of lysosomes (Supplemental Figure 6C), indicating lysosomal dysfunction. Importantly, CQ treatment blocked LC3B-II degradation and rescued ANPEP knockdown–induced reduction in SLC3A2 levels (Figure 8E). In Huh7 cells transfected with *shANPEP*, SLC3A2 accumulated inside lysosomes marked by Lysosomal-associated membrane protein 1 (LAMP-1; also known as lysosome-associated membrane glycoprotein 1) (Figure 8F), indicating knockdown of ANPEP facilitates SLC3A2 trafficking to lysosome. Ubiquitination of plasma membrane proteins is important for their trafficking to lysosome through clathrin-independent endocytosis (34, 36). Indeed, after CQ treatment, overexpression of ANPEP decreased ubiquitination of SLC3A2 in Huh7 cells (Figure 8G), whereas knockdown of ANPEP increased ubiquitination of SLC3A2 in HepG2 cells with or without DEX treatment (Figure 8H). Therefore, ANPEP regulates SLC3A2 stability through lysosomal dependent degradation.

MARCH8, an E3 ligase, mediates ubiquitination and lysosomal trafficking of SLC3A2 (34). Consistently, overexpression of MARCH8 reduced SLC3A2 protein levels and promoted its accumulation in lysosomes (Supplemental Figure 6, D and E). Interestingly, overexpression of ANPEP blocked MARCH8-mediated SLC3A2 protein level reduction and lysosomal trafficking (Figure 8, I and J). Further investigation showed that overexpression of ANPEP attenuated the interaction between SLC3A2 and MARCH8. Meanwhile, ANPEP knockdown enhanced the interaction between these 2 proteins (Figure 8K). Consequently, overexpression of ANPEP decreased MARCH8-mediated ubiquitination of SLC3A2, while ANPEP knockdown displayed opposite results (Figure 8L). Together, these data demonstrate that ANPEP blocks MARCH8-mediated SLC3A2 ubiquitination and lysosomal dependent degradation.

### Targeting SLC3A2 reverses chronic stress–induced tumor progression.

**Figure 9:**
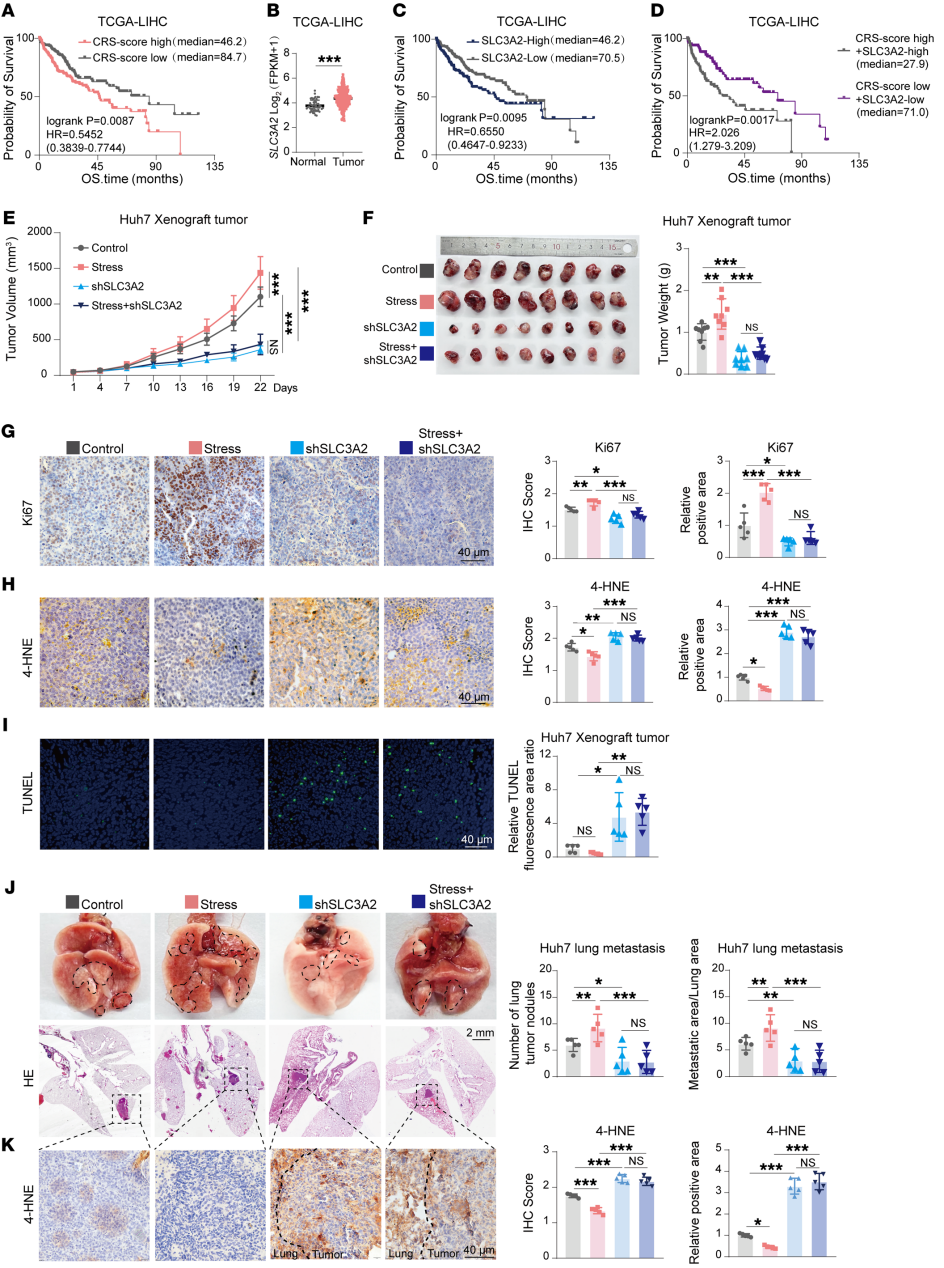
SLC3A2 as a therapeutic target to counteract the tumor-promoting effects of chronic stress. (**A**–**D**) Kaplan-Meier survival analysis and SLC3A2 expression were performed using specimens from the TCGA-LIHC cohort. Patients were stratified based on the median of chronic stress signature (CRS) scores (**A**). The expression of SLC3A2 in normal tissues (*n* = 50) and tumor tissues (*n* = 369) was compared (**B**). Patients were grouped according to SLC3A2 expression levels for survival analysis (**C**). Individuals with HCC were separated based on both CRS scores and SLC3A2 expression levels for survival analysis (**D**). (**E**–**I**) Nude mice were subjected to 1 week of restraint stress before subcutaneous injection of Huh7 cells (shNC or shSLC3A2) to establish xenograft tumors, with stress maintained throughout the experiment. Tumor growth (**E**), tumor weight and tumor images (**F**), Ki67 (**G**) and 4-HNE staining (**H**), and TUNEL staining (**I**) were assessed (**E** and **F**, *n* = 8; **G**–**I**, *n* = 5). (**J** and **K**) A lung metastasis model was established by tail vein injection of Huh7 cells (shNC or shSLC3A2) after 1 week of restraint stress. Representative lung images and H&E staining, as well as 4-HNE staining of tumor nodules, are shown with quantification (*n* = 5). Data are presented as mean ± SD. Statistical significance was determined by 2-tailed unpaired Student’s *t* test (**B**) and log-rank (Mantel-Cox) test (**A**, **C**, **D**), 2-way ANOVA with Šidák’s multiple comparisons test (**E**), or 1-way ANOVA with Tukey’s multiple comparisons test (**F**–**K**). *P < 0.05; **P < 0.01; ***P < 0.001.

To evaluate the clinical relevance of our findings, we established a chronic stress gene signature (CRS) using our RNA-Seq data following a previously described method (37). As shown in Figure 9A, the TCGA-LIHC patients were separated into 2 groups according to the median of CRS score. Notably, patients with low CRS scores were associated with better survival than individuals with high CRS scores, indicating this CRS could be used for predicting prognosis in liver cancer. Furthermore, the prognostic value of the CRS was validated across multiple tumor types in TCGA cohorts, including TCGA-ACC, TCGA-ESCA, TCGA-GBM, and others, demonstrating its broader applicability in diverse cancer types (Supplemental Figure 7). Meanwhile, SLC3A2 was highly expressed in the liver tumor tissues compared with normal liver specimens (Figure 9B). As expected, high levels of SLC3A2 were associated with poor prognosis in HCC individuals (Figure 9C). Interestingly, the CRS score–low and SLC3A2-low groups displayed a more favorable prognosis than the CRS score–high and SLC3A2-high groups (Figure 9D), supporting the substantial correlation between chronic stress and SLC3A2 expression.

To evaluate SLC3A2 as a target for patients undergoing chronic stress, we conducted xenograft tumor and lung metastasis mouse models. In the xenograft tumor model, chronic stress promoted the growth of xenograft tumors. Knockdown of SLC3A2 inhibited xenograft tumor growth. Notably, knockdown of SLC3A2 reversed chronic stress–induced acceleration of xenograft tumor growth (Figure 9, E and F). Further examinations demonstrated that SLC3A2 knockdown reversed chronic stress–induced increase in Ki67-positive cells and decrease in 4-HNE levels and TUNEL-positive cells (Figure 9, G–I). In the lung metastasis model, chronic stress increased the number of tumor nodules and the tumor spread area, both of which were reversed by SLC3A2 knockdown (Figure 9J). More importantly, the chronic stress–induced decrease in 4-HNE levels in lung metastatic tumors was also reversed by SLC3A2 knockdown (Figure 9K). Above all, chronic stress decreases lipid peroxidation and promotes tumor progression, which is reversed by SLC3A2 knockdown.

## Discussion

Chronic stress enhances production of glucocorticoids, which promotes tumor progression through different biological processes, including promoting angiogenesis, proliferation, and metastasis and suppressing immunosurveillance (4–6). In this study, chronic stress–induced glucocorticoids enhance ANPEP transcription via NR3C1. Further investigation showed that ANPEP interacts with SLC3A2 to block lysosome-dependent degradation of SLC3A2, leading to enhanced l-cystine intracellular transport and elevation of GSH levels, thereby suppressing lipid peroxidation–dependent ferroptosis. In vivo experiments revealed that the combination of sorafenib treatment and ANPEP silencing exerted a stronger tumor-inhibitory effect than sorafenib treatment alone. Knockdown of either ANPEP or SLC3A2 eliminates chronic stress–induced tumor progression. Clinical data validated SLC3A2 as a key regulator in the process of chronic stress–driven tumor progression. These findings reveal the involvement of psychological stress in reprogramming GSH metabolism in liver cancer cells.

These findings expand our understanding of the role of chronic stress in promoting tumorigenesis by identifying ANPEP-mediated GSH metabolic reprogramming in liver cancer under chronic stress. Metabolic reprogramming is a recognized hallmark of cancer. GSH metabolism, an important reprogrammed cancer metabolic hallmark, plays a pivotal role in tumor initiation, progression, and therapeutic resistance (12–16). GSH is synthesized in a 2-step, ATP-dependent process. GCL, a heterodimer of catalytic (GCLC) and modifier (GCLM) subunits, carries out a rate-limiting step in GSH synthesis (19). Genetic loss of *Gclm* has only 10%–25% of the GSH levels of wild-type mice and prevents tumors from undergoing malignant transformation (16, 38). In addition, GSH is the most abundant antioxidant, responsible for converting ROS to benign molecules, thereby maintaining redox balance and preventing ROS-caused lipid peroxidation and subsequent cell death (39–41). Therefore, administration of an engineered human cyst(e)inase enzyme mediates sustained depletion of the extracellular l-cysteine and l-cystine pool in mice and nonhuman primates and selectively causes cell cycle arrest and death in prostate cells due to depletion of intracellular GSH and ensuing elevated ROS (15). Our study using targeted amino acid metabolomics found that GSH metabolism is reprogrammed and intracellular l-cystine levels are elevated in ANPEP-overexpressed liver cancer cells. Further analyses demonstrated that ANPEP expression is positively correlated with intracellular GSH levels and inversely correlated with lipid peroxidation. Knockdown of ANPEP synergistically increases the tumor-inhibitory effect of sorafenib in liver cancer by enhancing lipid peroxidation–dependent ferroptosis. Our findings provide evidence for metabolic reprogramming in chronic stress–induced tumor progression. It is worth noting that amino acid profiling in serum or plasma can predict cancer progression and assist in early diagnosis (42, 43). Thus, our findings regarding amino acid changes under chronic stress may contribute to the identification of clinically useful metabolic biomarkers.

ANPEP, also known as CD13, is a type II transmembrane protein, mediating protein and peptide hydrolyzing, cell trafficking, signal transducing, antigen processing, and so on (44–47). Clinical examinations demonstrate that high ANPEP expression is associated with a poor prognosis, highly depending on tumor stage and type (24, 25). Our data revealed that ANPEP is elevated in liver cancer tissues, which is associated with worse overall survival. Interestingly, our data showed that ANPEP is transactivated by glucocorticoids, hormones that are induced by chronic stress in different types of tumors (11). Mechanistically, elevated glucocorticoids induce the nuclear translocation of NR3C1, which binds to the ANPEP promoter to drive its transactivation. Thus, we illustrated a molecular pathway for upregulation of ANPEP in liver cancer. A substantial body of literature has documented the effect of ANPEP on tumor progression. ANPEP-mediated phosphorylation of BCKDK promotes HCC metastasis and proliferation via the ERK signaling pathway (48). ANPEP is activated by angiogenic signals and is essential for capillary tube formation in human tumor xenografts (49). Bestatin, an inhibitor of ANPEP, is used as an immuno-enhancer in tumor therapy by enhancing T cell functions and improving NK cell activity (50, 51). Here, our data demonstrate that ANPEP upregulates GSH levels to block lipid peroxidation–dependent cell death in liver cancer. These findings further expand the mechanistic understanding of how ANPEP promotes tumor progression.

The other important finding of this study is that ANPEP stabilizes SLC3A2 by blocking MARCH8-mediated lysosomal trafficking and degradation of SLC3A2. SLC3A2, also known as 4F2hc, is involved in tumor progression through ferroptosis-dependent and -independent manners. SLC3A2/integrin interaction was required for adhesion-induced FAK phosphorylation and activation of downstream PI3K/AKT and MEK/ERK pathways, contributing to renal cancer cell proliferation and migration (52). SLC3A2 forms the glutamate-cystine antiporter system X_C_^–^ with SLC7A11 to mediate intracellular transport of l-cystine, blocking ferroptosis to promote tumor progression (41, 53, 54). Besides, SLC3A2 forms a complex with other amino acid transporters, such as SLC7A5-8 and SLC7A10, to regulate various amino acids in tumor cells (55, 56). Given that amino acids serve as critical metabolic fuels for tumor progression (57), SLC3A2 may exert broader oncogenic effects by stabilizing multiple amino acid transporters, thereby enhancing transportation of amino acids. In this study, ANPEP inhibits SLC3A2 lysosomal trafficking and degradation to inhibit ferroptosis in liver cancer cells. Further investigation illustrated that ANPEP disrupts the interaction between MARCH8 and SLC3A2. MARCH8, an E3 ligase, ubiquitinates SLC3A2 for lysosomal trafficking and degradation (34). These findings provide evidence for the role of SLC3A2 in ferroptosis. Additionally, clinical analysis revealed that liver cancer patients with high levels of chronic stress and SLC3A2 expression showed poorer overall survival. In vivo experiments verified that knockdown of SLC3A2 abolishes chronic stress–induced tumor progression by restoring lipid peroxidation–dependent ferroptosis. Ferroptosis, a form of programmed cell death driven by iron-dependent lipid peroxidation, is regulated by multiple cellular metabolic events, including redox homeostasis (58). Several studies implicate oxidative stress as a key driver of chronic stress–caused pathology in various organs (59–61). Our study provides evidence for direct connection of chronic stress with lipid peroxidation–dependent ferroptosis in tumor progression.

In summary, our study illustrates that ANPEP-mediated SLC3A2 stability and subsequent GSH metabolic reprogramming are critical drivers of chronic stress–induced tumor progression. Given that various stress paradigms, such as social defeat stress, unpredictable mild stress, and isolation stress, engage distinct neuroendocrine and metabolic pathways (62–64), future investigations using these complementary models will be valuable to determine whether the ANPEP/SLC3A2/GSH axis represents a generalizable mechanism linking stress to cancer progression. Nevertheless, our findings open avenues for developing metabolically targeted cancer therapies under physiological stress.

## Methods

Further information can be found in Supplemental Methods.

### Sex as a biological variable.

Male mice were used exclusively in all animal experiments. The restraint stress model is highly sensitive to hormonal fluctuations; therefore, male mice were selected to avoid variability introduced by the estrous cycle in females, which can substantially influence HPA axis reactivity, corticosterone secretion, and stress-related physiological responses. For clinical samples, sex was not analyzed as a biological variable because preliminary assessments indicated that sex did not influence the key phenotypic or molecular outcomes relevant to this study.

### Cell lines and generation of stable cell lines.

HepG2, Huh7, HCT116, Hepa1-6, and HEK293 cell lines were cultured in DMEM supplemented with 10% fetal bovine serum (FBS; Gibco, 10100147C) and 1% penicillin/streptomycin (Beyotime, C0222). The mouse colorectal cancer cell line CT26 and the mouse hepatoma cell line H22 were maintained in RPMI 1640 medium supplemented with 10% FBS and 1% penicillin/streptomycin. All cells were incubated at 37°C in a humidified atmosphere containing 5% CO_2_. All cell lines were obtained from the National Collection of Authenticated Cell Cultures (Shanghai, China).

HCC cells, stably overexpressing and/or knocking down ANPEP and/or SLC3A2, were infected with corresponding lentiviruses in the presence of 10 μg/mL polybrene (Beyotime, C0351). At 48 hours after the infection, the medium was changed, and cells were selected with puromycin (2 μg/mL, Sigma-Aldrich).

### Plasmids, shRNAs, siRNAs, and transfection.

Plasmids for the ectopic overexpression of target human proteins, including Flag-tagged ANPEP, Flag-tagged NR3C1, Flag-tagged SLC3A2, Myc-tagged MARCH8, HA-tagged SLC3A2, and HA-tagged ubiquitin, were purchased from MIAOLING BIOLOGY (Wuhan, China). Myc-tagged SLC7A11 was a gift from Bo Chu (Department of Cell Biology, Basic Medical Sciences, Shandong University). Flag-tagged shANPEP-resistant ANPEP-overexpression plasmid, PLko.1-egfp-puro-shANPEP and PLko.1-egfp-puro-shSLC3A2 plasmids, and their corresponding control were established in our lab. The shRNA sequences are listed in Supplemental Table 7.

siRNA targeting NR3C1 and the corresponding negative control were synthesized by Sangon Biotech, and the sequences are provided in Supplemental Table 7.

Plasmids or siRNAs were transfected into HEK293 cells or liver cancer cells using Lipofectamine 2000 (Thermo Fisher Scientific). After incubation at 37°C for the indicated durations, the transfected cells were harvested for subsequent experiments.

### Mouse work with chronic restraint stress.

Nude, BALB/c, and C57BL/6 mice were purchased from Vital River (Beijing, China) and housed under specific pathogen–free conditions at the Laboratory Animal Center of Shandong University.

For the chronic restraint model, 5-week-old male mice were housed in cages for 7 days to acclimate to the environment. Subsequently, the stressed group was subjected to horizontal restraint in an adjustable cylindrical plastic restrainer for 6 hours per day (10:00–16:00) over 21 consecutive days. Control mice remained undisturbed in their home cages but were similarly deprived of food and water during the restraint periods to match the conditions of the stress group.

For the open field test, 1 week after restraint, mice were assessed for locomotor activity. The test was performed in an open box (50 × 50 × 40 cm) under white light in a quiet room. Mice were habituated to the testing environment for 2 hours before the experiment. Each mouse was placed in the central zone of the open field and allowed to explore for 5 min. Mouse movements were recorded and analyzed using an automatic tracking system (Duoyi). The central area was defined as 50% of the total area, while the surrounding space was considered the peripheral zone. The apparatus was cleaned with 75% ethanol between each trial.

For the allograft tumor model, after 1 week of restraint, 2 × 10^6^ CT26 or H22 cells (100 μL) were subcutaneously injected into the axilla of 5-week-old BALB/c or C57BL/6 mice. Nude mice were subcutaneously injected with 1 × 10^7^ Huh7 cells with stably indicated gene overexpression or knockdown to establish xenograft tumors. To investigate the effect of chronic stress on sorafenib therapeutic efficacy, 7 days after tumor formation, mice received intraperitoneal injections of sorafenib at a dose of 50 mg/kg once every 3 days, for a total of 6 treatments. To investigate the effect of ANPEP on the therapeutic efficacy of IKE, mice were administered intraperitoneal injections of IKE (50 mg/kg) once daily for 6 consecutive days, starting 7 days after tumor establishment.

Tumor volumes were measured every 2 or 3 days using a sliding caliper and calculated with the formula volume = (length × width²)/2. The endpoint was defined as tumor growth reaching 2,000 mm³. Tumor tissues were collected and stored at –80°C or were processed into paraffin sections for further analysis.

For the lung metastatic model, 5-week-old BALB/c mice were subjected to 1 week of restraint stress, followed by intravenous injection of 1 × 10^6^ H22 cells (200 μL) via the tail vein. For studying the effect of ANPEP in chronic stress–induced lung metastasis, 6-week-old nude mice were intravenously injected with 4 × 10^6^ stably ANPEP-overexpressed or -knockdown and control Huh7 cells (200 μL) via the tail vein. The study was concluded at 2 months after injection or when mice exhibited signs of respiratory distress. Mouse lung tissues were harvested and fixed in 4% paraformaldehyde (PFA) for 48 hours before further analysis.

For the orthotopic Hepa1-6 HCC model, 6-week-old mice were subjected to daily restraint stress for 1 consecutive week prior to tumor implantation. For orthotopic liver tumor establishment, mice were anesthetized and placed on a sterile surgical field. After a midline laparotomy, the liver was gently exposed. A suspension of 3 × 10^6^ Hepa1-6–luciferase cells in 100 μL sterile PBS was slowly injected into the left lobe of the liver using a 30-gauge needle. Gentle pressure was applied to the injection site for approximately 1 minute to prevent bleeding and cell leakage, after which the abdominal wall and skin were sutured in layers. Mice were monitored until full recovery from anesthesia. Three weeks postimplantation, tumor growth was assessed by in vivo bioluminescence imaging, followed by euthanasia and tissue collection.

### Hormone level measurement.

Blood samples were collected at specified time points and stored at –80°C until analysis. Mouse plasma corticosterone levels were measured using ELISA kits from Sangon Biotech (KB13AD0358) according to the manufacturer’s instructions.

### Tissue microarray and immunostaining.

Tissue microarray slides containing 150 specimens from 76 patients with HCC were obtained from Outdo Biotech (Shanghai, China). The detailed information of tumor specimens can be found in their website (Supplemental Table 9). Immunostaining was performed using an anti-ANPEP antibody, as detailed in Supplemental Table 8. Images of stained sections were taken with a microscope (Olympus). To quantify immunostaining, the integral optical density and the positively stained area were analyzed using ImageJ software.

### NR3C1 binding site prediction.

NR3C1 binding sites in the ANPEP promoter region were predicted using JASPAR (https://jaspar.elixir.no/) and ENCODE (https://www.encodeproject.org/).

### Dual-luciferase reporter assays.

The human ANPEP promoter and its truncated variants were subcloned into the pGL3-basic vector. A Renilla luciferase expression plasmid was cotransfected with the indicated firefly luciferase reporter constructs into HEK293 cells using Lipofectamine 2000 to normalize transfection efficiency.

For functional analysis, HEK293 cells were cotransfected with the pGL3-ANPEP construct and either an NR3C1-overexpression plasmid or siRNA targeting NR3C1. After 48 hours of incubation at 37°C, the transfected cells were harvested, and firefly luciferase activity was measured using the Dual-Luciferase Reporter Assay System (Promega), following the manufacturer’s instructions. The firefly luciferase activity was normalized to Renilla luciferase activity.

### DNA pull-down and Western blotting.

To prepare streptavidin-labeled DNA probes, biotinylated DNA oligonucleotides were annealed according to standard protocols. Streptavidin magnetic beads (Beyotime, P2151) were washed once with wash buffer prior to use. A pull-down reaction mixture containing 20 μL of streptavidin magnetic beads, capture buffer, 1 mg of nuclear protein extract, and biotin-labeled DNA was incubated at 4°C for 8 hours with gentle rotation. The beads were then washed 3 times with wash buffer (5 min each wash). After washing, 40 μL of 1× Laemmli sample buffer was added to the beads, and the mixture was boiled at 100°C for 10 min. Eluted proteins were subjected to Western blot analysis. The biotin-labeled DNA probe was synthesized by Sangon Biotech, and the sequence is listed in Supplemental Table 7.

### Cytoplasmic, cell membrane, and nuclear protein extraction.

Cytoplasmic and nuclear proteins were extracted from liver cancer cells using the Nuclear and Cytoplasmic Protein Extraction Kit (Beyotime, P0028) according to the manufacturer’s instructions. Cell membrane and cytoplasm proteins were extracted from liver cancer cells using the cell membrane protein and cytoplasm protein extraction kit (Beyotime, P0033) according to the manufacturer’s instructions. The separated cell membrane, cytoplasmic, and nuclear protein fractions were then used for subsequent Western blot analysis or DNA pull-down experiments, respectively.

### Flow cytometry.

Lipid peroxides were assessed using C11-BODIPY 581/591 (HY-D1301, MedChemExpress [MCE]) according to our laboratory protocol (65). Cells were trypsinized, resuspended in 400 μL of serum-free medium containing 10 μM C11-BODIPY 581/591, and incubated at 37°C for 30 min before analysis by flow cytometer (CytoFLEX, Beckman Coulter).

For intracellular ROS levels, cells were incubated with DCFHDA (5 μM, HY-D0940, MCE) in PBS for 30 min at 37°C, harvested with trypsin, and analyzed using a flow cytometer.

To evaluate the expression of SLC3A2 on the cell surface, HEK293 cells were first incubated with anti-human SLC3A2 primary antibodies for 30 min at 4°C (Supplemental Table 8). After washing, the cells were incubated with Alexa Fluor 488–conjugated goat anti-rabbit secondary antibody for an additional 30 minutes at 4°C. The surface expression of SLC3A2 was analyzed by flow cytometry.

For total SLC3A2 expression, a portion of the cells was permeabilized, and the same SLC3A2 antibody staining procedure was applied to detect the total protein. Flow cytometry analysis was performed as described above.

The term percent of max refers to the normalization method used in flow cytometry, in which the frequency of the highest peak in each histogram is set to 100%, and all other values are expressed as a percentage relative to this maximum. This allows for a direct comparison of the overall signal distribution between samples, independent of total cell count.

### Metabolite measurement.

Cysteine content in indicated liver cancer cells was measured using ELISA kits (YS07420B, YaJi Biological) according to the manufacturer’s instructions. Cystine content was quantified using Cystine ELISA kits (Sangon Biotech) following the provided protocol.

The levels of GSH and GSSG in indicated liver cancer cells were measured using the Glutathione (GSH) and Glutathione Disulfide (GSSG) Detection Kit (Beyotime, P0028), following the manufacturer’s instructions.

### RNA-Seq.

RNA was extracted from subcutaneous allograft tumors of H22 cells in normal and stress mice using TRIzol reagent (Invitrogen) for transcriptome analysis. Three biological replicates were included for each group. Sequencing was performed on the BGISEQ platform by BGI, with an average of 1.16 GB of data generated per sample. The average mapping rate to the reference genome was 90.73%, and the average mapping rate to genes was 79.27%. A total of 18,960 genes were identified. DEGs between the control and stress groups were analyzed using an online platform for data analysis and visualization (https://www.bioinformatics.com.cn, last accessed on December 10, 2024), with |log_2_FC| > 1 and *P* < 0.05 as the screening criteria. Gene Ontology analysis and other analyses were conducted and visualized using the same platform.

### Metabolomics sequencing and analysis.

For nontargeted metabolomics, the serum and orthotopic Hepa1-6 HCC samples from the normal and stress group mice were separated and stored at –80°C. The samples were sent to Metware for liquid chromatography-tandem mass spectrometry (LC-MS/MS) analysis, using dry ice for transportation. Upon arrival, the samples were removed from the –80°C freezer and thawed on ice until no visible ice remained (all subsequent steps were performed on ice). After thawing, the samples were vortexed for 10 sec to ensure uniformity. A 50 μL aliquot of each sample was transferred to a corresponding labeled centrifuge tube. Then, 300 μL of a 20% acetonitrile/methanol extraction solution containing internal standards was added, followed by vortexing for 3 min. The samples were then centrifuged at 13,800 × *g* for 10 min at 4°C. After centrifugation, 200 μL of the supernatant was transferred to a new labeled centrifuge tube and incubated at –20°C for 30 min. The mixture was then centrifuged again at 13,800 × *g* for 3 min at 4°C. Finally, 180 μL of the supernatant was transferred to LC-MS/MS autosampler vials with inserts for subsequent analysis. LC conditions were column: Waters ACQUITY UPLC HSS T3 C18, 1.8 μm, 2.1 mm × 100 mm; mobile phase A: ultrapure water with 0.1% formic acid; mobile phase B: acetonitrile with 0.1% formic acid; column temperature: 40°C; flow rate: 0.40 mL/min; injection volume: 2 μL.

For targeted amino acid metabolomics, we gently scraped Huh7-Vector and Huh7-ANPEP stably transfected cells from the cell culture dish. Then cells were rinsed twice with precooled PBS, freeze-dried in liquid nitrogen for 15 min, and sent for LC-MS/MS analysis (Metware) using solid CO_2_. Upon receipt, samples were taken from the –80°C freezer and thawed on ice (all subsequent procedures were performed on ice unless otherwise specified). We resuspended the cell pellets in 100 μL of ultrapure water extraction buffer containing protease inhibitors, PMSF, and EDTA. To prepare the sample for analysis, we mixed a 50 μL aliquot of the cell suspension with 200 μL of prechilled methanol (–20°C). We vortexed the mixture at 600 × *g* for 2 min, then snap-froze it in liquid nitrogen for 5 min. After thawing the sample on ice for 5 min, we vortexed again for 2 min. This freeze-thaw-vortex cycle was repeated 3 times. After the final cycle, we centrifuged the samples at 12,000 rpm for 10 min at 4°C and transferred 200 μL of the supernatant to a new centrifuge tube and stored at –20°C for 30 min. Subsequently, we centrifuged the samples again at 13,800 × *g* for 10 min at 4°C. Finally, we transferred 180 μL of the supernatant through a protein precipitation plate and stored it for subsequent LC-MS/MS analysis. For protein quantification, the remaining 50 μL of the cell suspension underwent 3 freeze-thaw cycles using liquid nitrogen, followed by centrifugation at 13,800 × *g* for 10 min. We collected the supernatant and determined protein concentration using the BCA protein assay (Thermo Fisher Scientific).

Analysis was performed using an ultra-performance liquid chromatography system (ExionLC AD, SCiex website https://sciex.com.cn/), coupled with an MS/MS system (QTRAP 6500+, SCiex website https://sciex.com.cn/).

Prior to differential analysis, PCA was conducted using the PTM BioLab cloud platform (https://www.ptm-biolab-css.com.cn/) to evaluate the variability within and between sample groups. In targeted amino acid metabolomics, metabolites exhibiting a fold-change greater than 2 or less than 0.5 between the control and experimental groups were considered substantially different. For untargeted amino acid metabolomics, metabolites with a fold-change greater than 1.5 or less than 0.67 were deemed noticeably altered. The MSEA was performed using MetaboAnalyst 5.0 (https://www.metaboanalyst.ca) based on the markedly altered metabolites identified from the targeted metabolomic analysis. The markedly enriched metabolic pathways in orthotopic liver tumors and serum samples are listed in Supplemental Table 5.

### Bioinformatic analysis.

To determine whether there was an association between stress exposure and the survival of patients with liver cancer, we generated a CRS following a previously described method (37). The CRS consists of the most highly expressed upregulated genes in the allograft tumors of mice subjected to chronic restraint stress compared with those from control tumors. We identified the top 150 genes that were the most upregulated in tumors from the stress group compared with tumors from the control group. To exclude the artifact of lowly expressed genes, we then ranked these top 150 upregulated genes based on the average of their RNA-Seq counts in all the samples (both control and stress) and took the top 30 highly expressed candidate genes as the CRS. The CRS genes are listed in Supplemental Table 10.

For the survival analysis, TCGA clinical data were downloaded from the data portal of the Genomic Data Commons, and GSE1898 clinical data were downloaded from NCBI GEO (27). For example, individuals of these 2 cohorts were separated in 2 groups according to the median of CRS score, respectively. GraphPad Prism 8 software was used to analyze the difference of survival in individuals with indicated gene expression. Kaplan-Meier model and log-rank test were used to determine the *P* value.

### Statistics.

Statistical analyses were performed GraphPad Prism 8 software. For comparisons between 2 groups, 2-tailed Student’s *t* test for unpaired or paired data was used. For multiple groups’ comparisons,1-way ANOVA and 2-way ANOVA were used. A *P* value of less than 0.05 was considered significant. Statistical details can be found in the figure legends.

### Study approval.

All animal experiments were approved by the Institutional Animal Care and Use Committee of the School of Basic Medical Sciences, Shandong University (ECSBMSSDU2019-2-029) and were conducted in accordance with the provisions of the Declaration of Helsinki. Human tissue microarray slides containing specimens were purchased from Outdo Biotech (Shanghai, China). Ethical approval and informed consent were obtained by the supplier, and no additional approval was required for this study.

### Data availability.

(a) TCGA datasets: Clinical information and gene expression matrices for TCGA-LIHC, TCGA-ACC, TCGA-ESCA, TCGA-GBM, TCGA-LGG, TCGA-LUSC, TCGA-STAD, TCGA-THYM, and TCGA-UVM analyzed in this study were obtained from the UCSC Xena platform (https://xena.ucsc.edu/). (b) RNA-Seq data generated in this study have been deposited in the NCBI Sequence Read Archive database (https://www.ncbi.nlm.nih.gov/bioproject) under accession number PRJNA1366058. The matrix of DEGs is provided in Supplemental Table 6. (c) Untargeted metabolomics data: The untargeted metabolomics sequencing data have been deposited in the BioProject database at https://ngdc.cncb.ac.cn/bioproject/browse/PRJCA051793 and https://ngdc.cncb.ac.cn/bioproject/browse/PRJCA051669 Processed metabolite expression matrices are provided in Supplemental Tables 1–4. (d) Targeted amino acid metabolomics data: The targeted amino acid metabolomics data have been deposited in the BioProject database at https://ngdc.cncb.ac.cn/bioproject/browse/PRJCA051673 (e) ChIP-Seq datasets: Publicly available ChIP-Seq datasets used in this study were obtained from the ENCODE project (https://www.encodeproject.org/). (f) Protein-protein interaction analyses: Predicted protein interaction data were generated using the STRING (https://cn.string-db.org/) and BioGRID (https://thebiogrid.org/). Reagents and animal models used in this study are available from the corresponding author upon request. Numerical source data for all plotted values are provided in the Supporting Data Values file. All other data supporting the findings of this study are included in the article and its supplement.

## Author contributions

YW performed the experiments, analyzed the data, and wrote the manuscript; YZ, XS, MW, MS, YF, and WM helped conduct the experiments and read the manuscript; XJ, DF, and MZ helped analyze the data and read the manuscript; ZW, CL, LG, XL, and CM reviewed the manuscript; XY designed and supervised the experiments and wrote and reviewed the manuscript.

## Funding support

- National Science Foundation of China (NO. 82470609, NO. 81972819).
- Natural Science Foundation of Shandong Province (NO. ZR2020YQ57).

## Supplementary Material

Supplemental data

Unedited blot and gel images

Supplemental table 1

Supplemental table 10

Supplemental table 2

Supplemental table 3

Supplemental table 4

Supplemental table 5

Supplemental table 6

Supplemental table 7

Supplemental table 8

Supplemental table 9

Supporting data values

## Version 1 12/02/2025

In-Press Preview

## Version 2 02/16/2026

Electronic publication
