# Expression of Concern for Chronic stress–induced ANPEP drives liver cancer progression by increasing glutathione synthesis and inhibiting ferroptosis

**DOI:** 10.1172/JCI213290

**Published:** 2026-10-01

**Authors:** Yongkang Wu, Yankun Zhang, Xiaojia Shi, Mengting Wu, Min Sun, Ying Feng, Wenmeng Ma, Xiule Jiang, Dingqi Fei, Mingjian Zhao, Zhuanchang Wu, Chunyang Li, Xiaohong Liang, Lifen Gao, Chunhong Ma, Xuetian Yue

Original citation: *J Clin Invest.* 2026;136(4):e195685. https://doi.org/10.1172/JCI195685

Citation for this expression of concern: *J Clin Invest.* 2026;136(19):e213290. https://doi.org/10.1172/JCI213290

The authors recently alerted the Editors to errors pertaining to figures, supporting data values, and/or analysis of Figures 4I and 9K and Supplemental Figures 2H, 2I, 4H, and 5M that were identified in response to an institutional mandate for a post-publication audit of the data. Although the authors have indicated that the errors were due to inadvertent mistakes, the Editors are issuing this Expression of Concern to alert readers to the issues. We have requested institutional oversight into this matter and will alert readers to the outcome when the institutional evaluation is complete.

## Version 1 10/01/2026

Electronic publication

