# Supplementary material for: Chronic stress–induced ANPEP drives liver cancer progression by increasing glutathione synthesis and inhibiting ferroptosis
Source: J Clin Invest. 2025 Dec 2;136(4):e195685. doi: 10.1172/JCI195685 (PMC12904706; doi:10.1172/JCI195685)
Supplement: Supplemental data [file jci-136-195685-s306.pdf]

Supplemental Figures

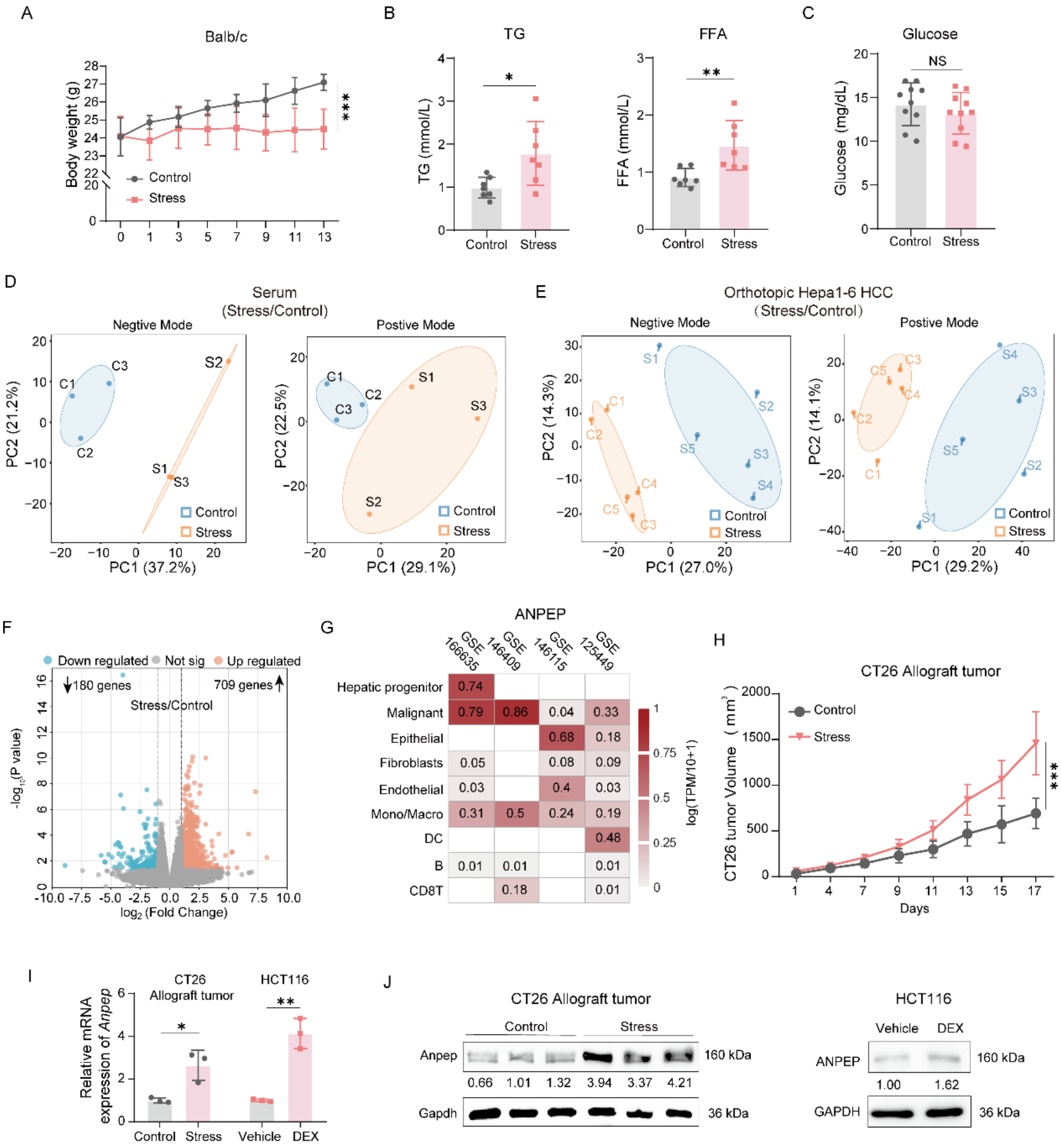

Figure S1. Chronic stress reprograms amino acid metabolism and upregulates ANPEP expression in liver cancer. (**A**) Body weight changes in control and stressed mice (n = 8). (**B**) Serum triglyceride (TG) and free fatty acid (FFA) levels in control and stressed mice after 7 days of stress (n = 7). (**C**) Serum glucose levels in control and stressed mice after 7 days of stress (n = 10). (**D**, **E**) Principal component analysis (PCA) of untargeted metabolomic profiles from serum and orthotopic Hepa1-6 tumor samples in control and stressed mice (D, n =3; E, n =5). (**F**) The volcano plot illustrated the distribution of differentially expressed genes identified by RNA-seq (Stress vs. Control). (**G**) The heatmap displayed relative ANPEP expression across various cell types according to publicly available single-cell RNA sequencing datasets. (**H**) CT26 murine colon cancer cells were subcutaneously implanted into control and stress mice. Tumor growth curves were monitored (n = 5). (**I**, **J**) ANPEP mRNA and protein levels in primary CT26 tumors from control and stressed mice, and in HCT116 cells cultured with or without 1 μM dexamethasone (DEX) for 48 h (n = 3). Data are presented as mean ± SD. Significance was

assessed by two-way ANOVA with Sidak's multiple comparisons test (**A**, **H**) or two-tailed unpaired Student's t test (**B**, **C**, **I**). NS, No significance; * $P < 0.05$; ** $P < 0.01$; *** $P < 0.001$.

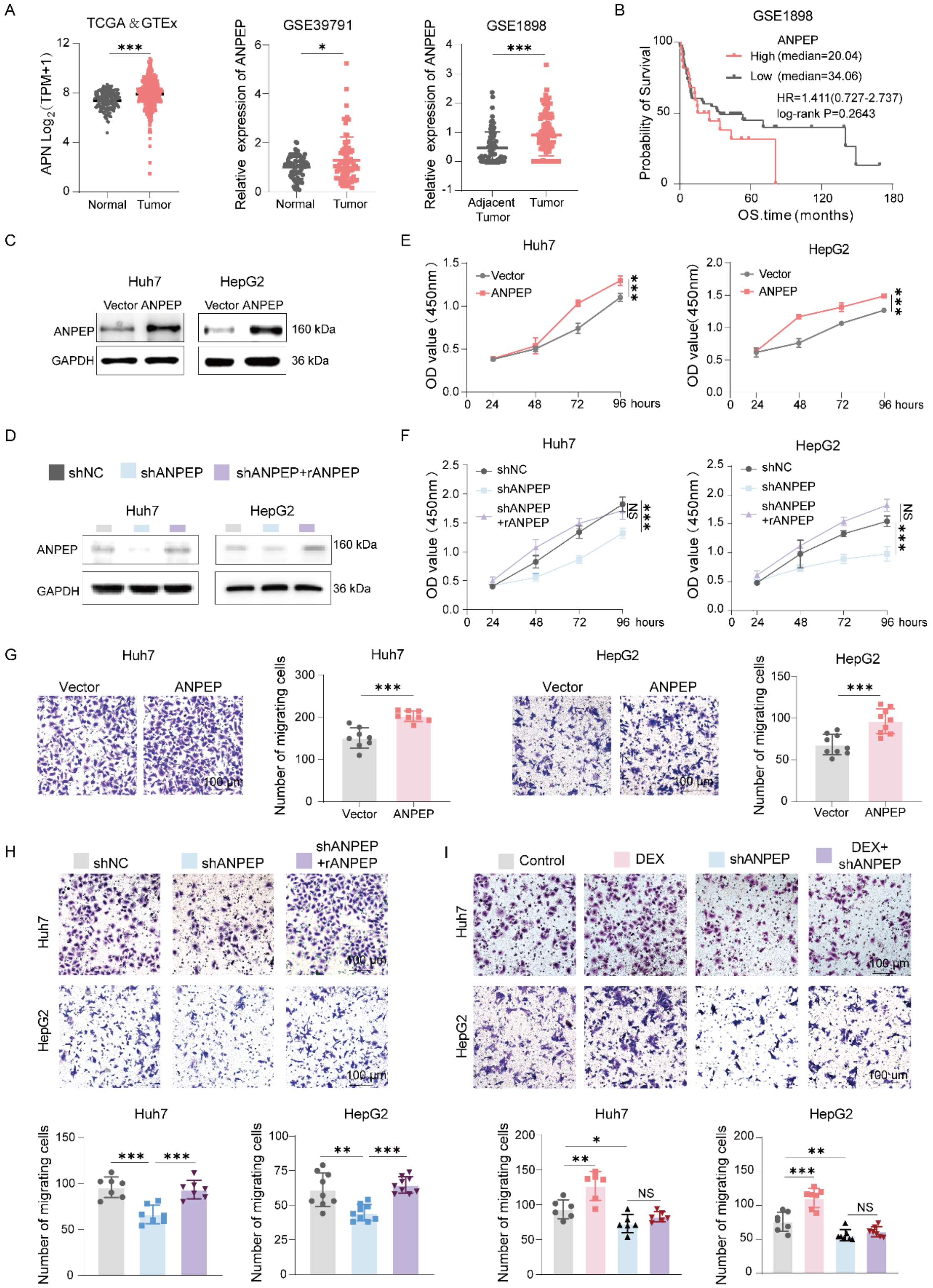

Figure S2. ANPEP plays a crucial role in chronic stress-induced liver cancer progression. (**A**) Relative ANPEP expression in normal liver and HCC tissues from TCGA and GTEx datasets (Normal, n = 160; Tumor, n = 369) and GEO cohorts (GSE39791: Normal, n = 71; Tumor, n = 71; GSE1898: Adjacent, n = 76; Tumor, n = 76). (**B**) Kaplan–Meier survival analysis of HCC patients from the GSE1898 cohort stratified by ANPEP expression (ANPEP-high, n = 22; ANPEP-low, n = 54). (**C**, **D**) Immunoblots of liver cancer cells with stable ANPEP manipulation. (**E**, **F**) Huh7 and

HepG2 cells with ANPEP manipulation were cultured in 96-well plates. Cell growth was monitored using the CCK-8 assay at the indicated time points (n= 3 ~ 5). (**G**, **H**) Transwell assays were performed to evaluate the effect of ANPEP manipulation on the migration of Huh7 and HepG2 cells (n = 9). (**I**) ANPEP-knockdown Huh7 and HepG2 cells were treated with 1 μM DEX for 48 h, followed by transwell assays (n = 7). Data are presented as mean ± SD. Significance was assessed by unpaired two-tailed Student's t test (**A**, **G**), Log-rank (Mantel–Cox) test (**B**), two-way ANOVA with Sidak's multiple comparisons test (**E**, **F**), and one-way ANOVA with Tukey's multiple comparisons test (**H**, **I**). NS, No significance; * $P < 0.05$; ** $P < 0.01$; *** $P < 0.001$.

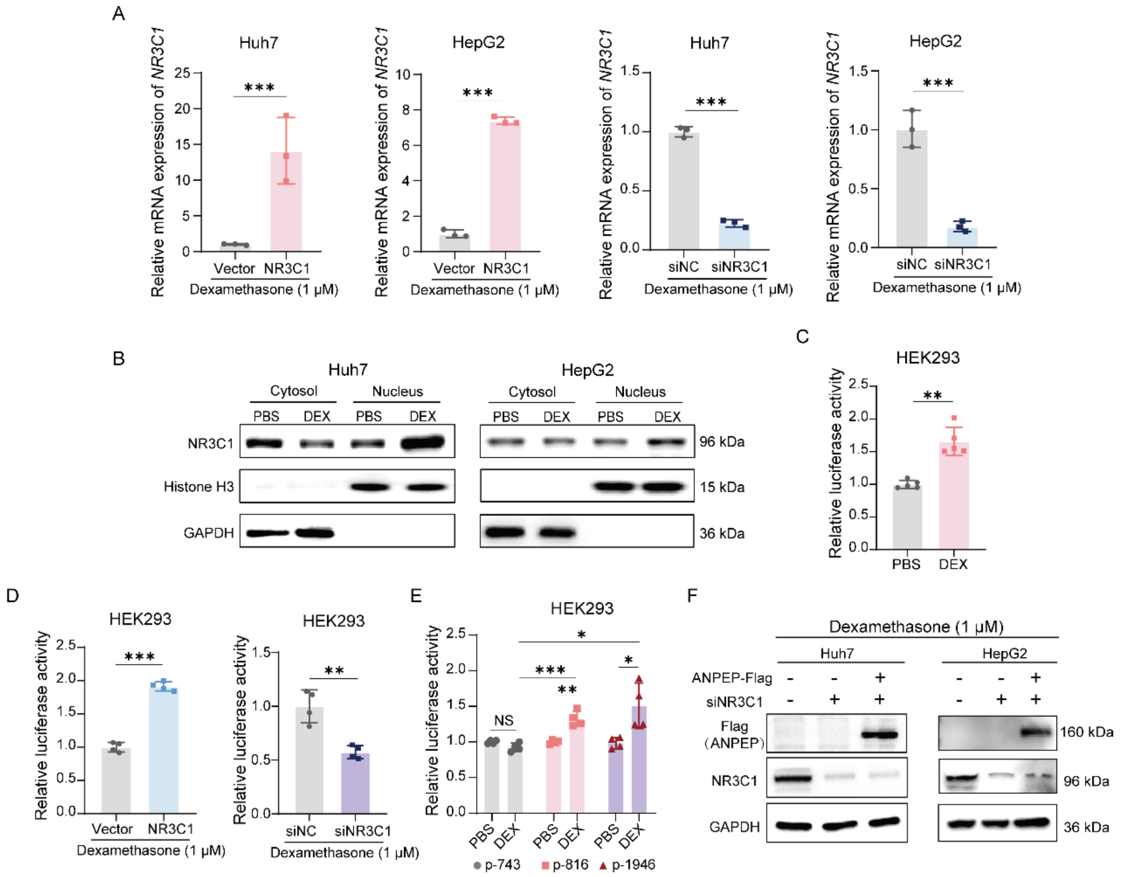

Figure S3. Glucocorticoids promote ANPEP transcription *via* NR3C1. (**A**) NR3C1 mRNA levels in Huh7 and HepG2 cells with NR3C1 overexpression or knockdown, followed by 48 h DEX treatment (n = 3). (**B**) Huh7 and HepG2 cells were treated with DEX to isolate cytoplasmic and nuclear fractions. NR3C1 protein levels were examined in these fractions by Western blotting. (**C**, **D**) ANPEP promoter activity in HEK293 cells were measured after 48 h DEX treatment (**C**) or NR3C1 manipulation plus DEX treatment (**D**) (**C**, n = 5; **D**, n = 4). **(E)** HEK293 cells were transfected with ANPEP-truncated reporters to measure luciferase activity after DEX treatment (n=4). (**F**) ANPEP and NR3C1 protein levels in NR3C1-knockdown Huh7 and HepG2 cells with or without ANPEP overexpression, after 48 h DEX treatment. Data are presented as mean ± SD. Statistical significance was determined by two-tailed unpaired Student's t test (**A**, **C-E**). NS, No significance; * $P < 0.05$; ** $P < 0.01$; *** $P < 0.001$.

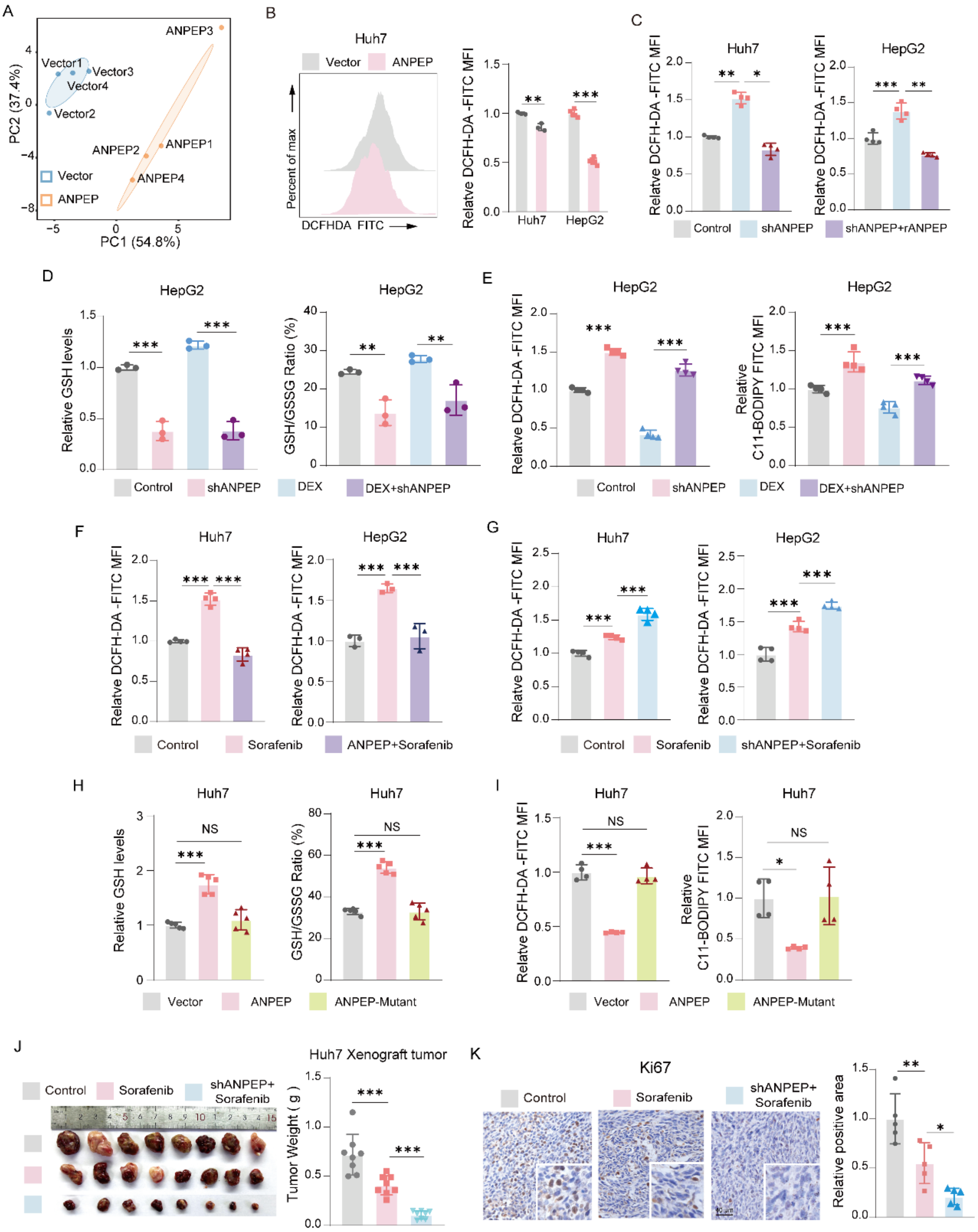

Figure S4. ANPEP reduces intracellular ROS levels and sorafenib tumor inhibitory effect. (**A**) Principal component analysis (PCA) was performed on the targeted amino acid metabolomic profiling data (n = 4). (**B**, **C**) Total ROS levels in ANPEP-manipulated Huh7 and HepG2 cells were measured by DCFH-DA staining and flow cytometry (**B**: Huh7, n = 3, HepG2, n = 5; **C**: n = 4). (**D**, **E**) GSH levels, GSH/GSSG ratios, and DCFH-DA and C11-BODIPY intensity in ANPEP-manipulated HepG2 cells after 48 h DEX treatment (**D**, n = 3; **E**, n = 4). (**F**, **G**) Total ROS levels in liver cancer cells treated with sorafenib alone or sorafenib plus ANPEP manipulation (**F**, n = 3; **G**, n = 4). (**H**, **I**) GSH levels and GSH/GSSG ratios, and DCFH-DA and C11-BODIPY intensity in Huh7 cells transfected with ANPEP or mutant ANPEP (**H**, n = 5; **I**, n = 4). (**J**, **K**) Xenograft tumor model established by subcutaneous injection of Huh7 cells with or without ANPEP manipulation.

Tumor weight (**J**) and Ki-67-positive cells (**K**) were assessed (J, n = 8; K, n = 5). Data are presented as mean ± SD. Statistical significance was determined by two-tailed unpaired Student's t test (B)and one-way ANOVA with Tukey's multiple comparisons test (C-K). NS, No significance; * $P < 0.05$; ** $P < 0.01$; *** $P < 0.001$.

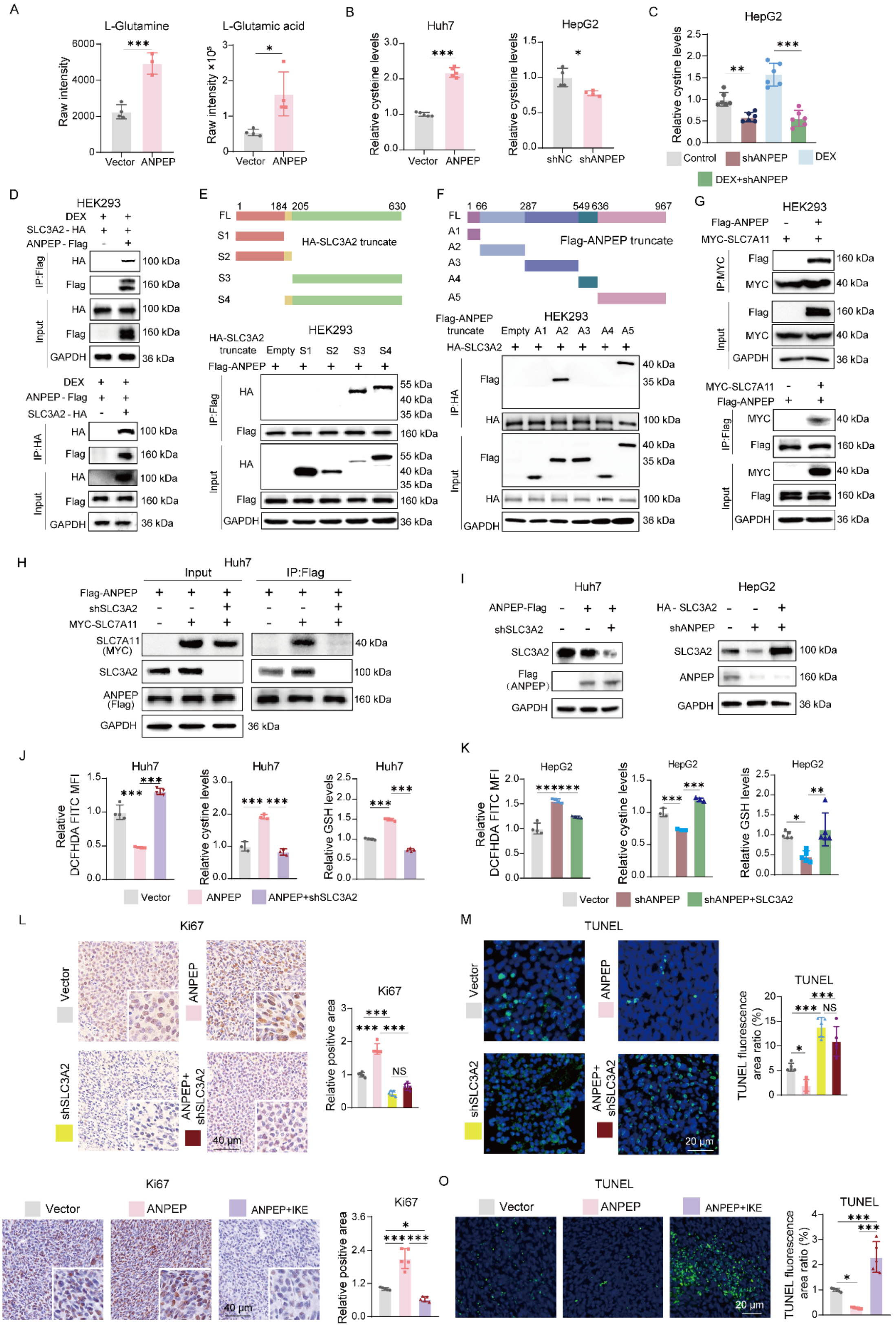

Figure S5. ANPEP interacts with SLC3A2 to reduce ROS levels and promote tumor growth. (**A**) L-Glutamine and L-Glutamic acid were identified as a differential metabolite by targeted amino acid metabolomics (n = 4). (**B**) L-cysteine levels were examined in liver cancer cells (Huh7, n=5; HepG2, n=4). (**C**) L-cystine levels were examined in ANPEP manipulation HepG2 cells with or without DEX treatment (n=6). (**D**) Exogenous ANPEP-SLC3A2 interactions were

detected in HEK293 cells transfected with HA-tagged SLC3A2 and/or Flag-tagged ANPEP after 48 h of DEX treatment, using Co-IP assays followed by Western blotting. (**E**, **F**) Co-IP assays were performed in HEK293 cells transfected with Flag-tagged ANPEP (truncates) and HA-tagged SLC3A2 (truncates). (**G**) HEK293 cells were transfected Flag-tagged ANPEP and/or Myc-tagged SLC7A11 to perform IP using anti-Flag or anti-Myc antibody, respectively. (**H**) ANPEP-SLC7A11 interaction was examined in Flag-tagged ANPEP and Myc-tagged SLC7A11 transfected Huh7 cells with or without SLC3A2 by IP assay and followed by Western blotting. (**I**) Western blot validation of SLC3A2 knockdown or overexpression efficiency in Huh7 and HepG2 cells with ANPEP knockdown or overexpression. (**J**, **K**) DCFH-DA intensity, GSH and L-cystine levels were measured in Huh7 or HepG2 cells (DCFH-DA, n=4; GSH, n=3; L-cystine, n=5). (**L-O**) Xenograft tumors were established by subcutaneous injection of Huh7 cells stably expressing ANPEP, ANPEP/shSLC3A2, or ANPEP-manipulated cells treated with IKE or vehicle. Ki67- and TUNEL-positive cells were assessed (n = 5). Data are presented as mean ± SD. Statistical significance was determined by two-tailed unpaired Student's t test (**A**, **B**) and one-way ANOVA with Tukey's multiple comparisons test (**C**, **J-O**). NS, No significance; * $P < 0.05$; ** $P < 0.01$; *** $P < 0.001$.

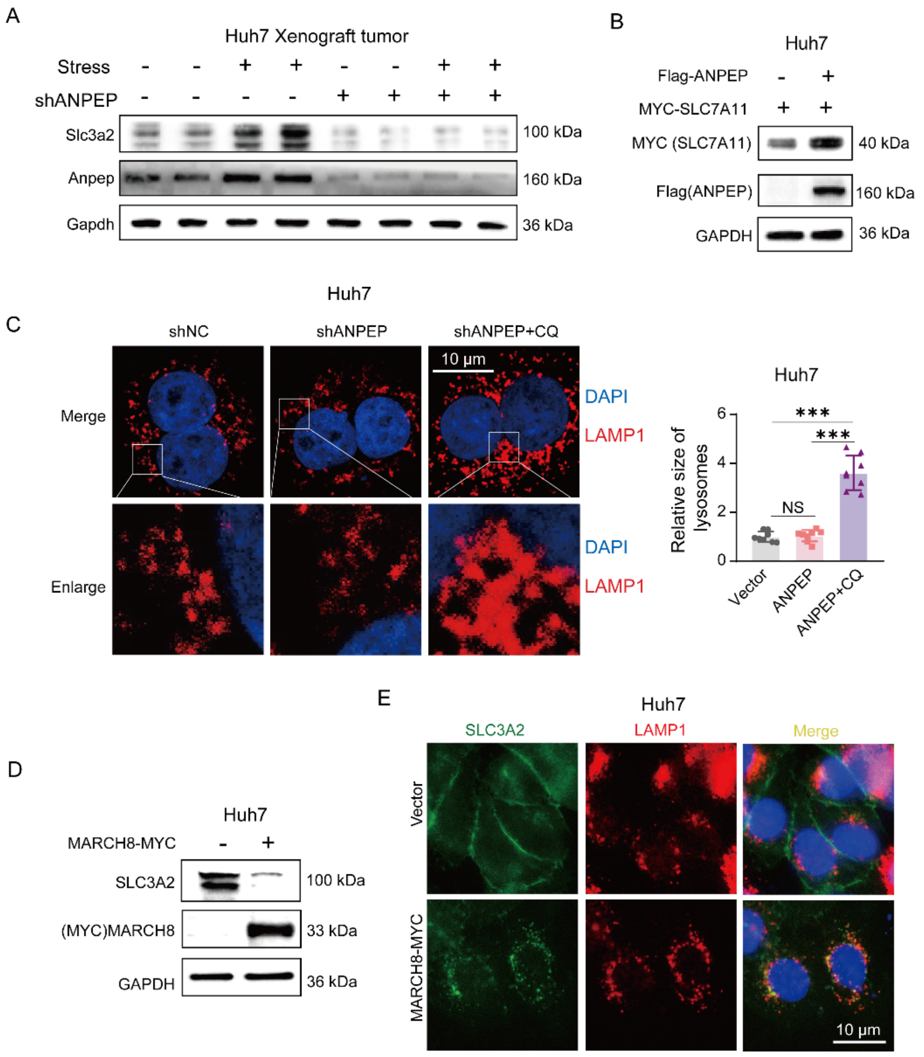

Figure S6. MARCH8 mediates SLC3A2 lysosomal trafficking and degradation. (**A**) Protein levels of SLC3A2 and ANPEP were analyzed in Huh7 xenograft tumors. **(B)** Western blot analysis of SLC7A11 levels in Huh7 cells with ANPEP overexpression. (**C**) Lysosomes were labeled with LAMP1 to assess morphological changes in CQ-treated Huh7 cells with or without ANPEP knockdown. Lysosome size was quantified (n = 7). (**D**) The SLC3A2 protein levels were analyzed in MARCH8-transfected Huh7 cells by Western blotting. (**E**) The SLC3A2 lysosomal trafficking was examined in Huh7 cells transfected with MARCH8 or control plasmid by immunofluorescence. Data are presented as mean ± SD. Statistical significance was determined by one-way ANOVA with Tukey's multiple comparisons test (**D**). NS, No significance; *** $P < 0.001$.

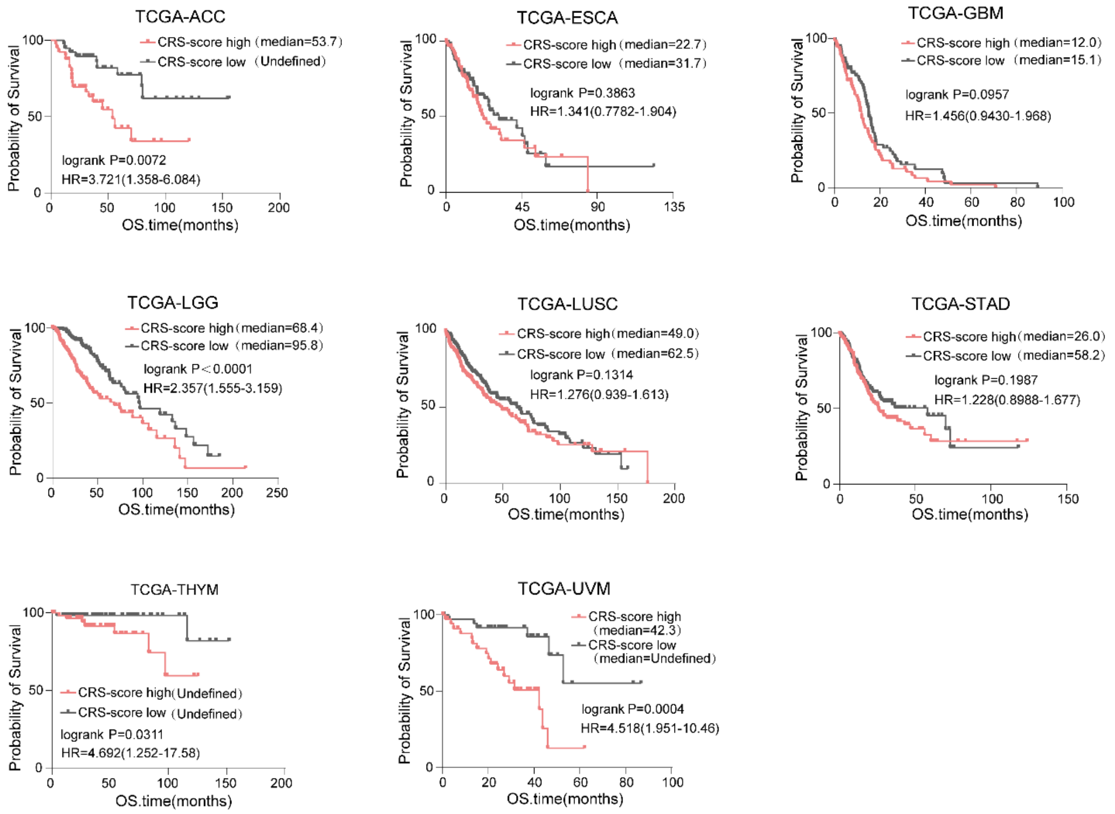

Figure S7. Validation of the prognostic value of chronic stress gene signature (CRS) across multiple tumor types based on TCGA cohorts. Kaplan-Meier survival analysis was performed using specimens from the TCGA-ACC, TCGA-ESCA, TCGA-GBM, TCGA-LGG, TCGA-LUSC, TCGA-STAD, TCGA-THYM, and TCGA-UVM cohorts. Patients were stratified based on the median of chronic stress signature (CRS) scores. Statistical significance was determined by Log-rank (Mantel–Cox) test.

Supplement methods

Quantitative real-time PCR. Total RNA was extracted from cells or mouse tissues using RNA-Quick Purification kits (ESscience, RN001) or TRIzol (Sangon Biotech, B511311), respectively. RNA concentration was measured by NanoDrop One Analyzer (Thermo Scientific). Complementary DNA was synthesized by HiScript III RT SuperMix with 500 ng total RNA according to manufacturer's protocol (Vazyme, R323). Quantitative RT-PCR reactions were composed with cDNA, SYBR Green Mix (Vazyme, Q711) and primers. Reactions were conducted on a quantitative thermal cycler (Vazyme) and genes of interest were normalized by GAPDH or β-Actin. The primer sequences used are listed in Supplemental Table 7.

Western Blot. Cells and mouse tissues were lysed in NP-40 lysis buffer (Beyotime, P0013F) containing 1 mM protease inhibitor phenylmethanesulfonyl fluoride (PMSF) (Beyotime, ST505) and phosphatase inhibitor mini tablets (Thermo Scientific, A32957). Protein supernatant was obtained to measure protein concentration using Bradford Assay Kit (Beyotime, P0006). Equal amounts of protein (20 μg) were separated by 8–12% SDS-PAGE. After electrophoresis, proteins were transferred onto PVDF membrane (Merck-millipore, IPVH00010) and blocked with 5% skim milk in TBST. Membranes were incubated with the indicated primary antibodies, followed by HRP-conjugated secondary antibodies. Signals were detected using an enhanced chemiluminescence (ECL) kit (Thermo Scientific, 32132) and visualized with a chemiluminescence imaging system (Tanon). Primary antibodies used for blotting are listed in Supplemental Table 8.

Immunohistochemistry (IHC). Formalin-fixed, paraffin-embedded tumor tissue sections (5 μm) were dewaxed, rehydrated, and incubated with primary antibodies (Supplementary Table 8) overnight at 4 ℃, followed by secondary antibodies for 2 h at room temperature. Immunoreactivity

was visualized using 3,3′-diaminobenzidine (DAB, Zsbio, ZLI-9018). Images were captured by microscope (Olympus DP80, Japan). Positively stained areas were quantified using ImageJ software. The staining intensity and the percentage score were quantified using ImageJ software. The intensity score multiplied by the percentage score was used as the immunohistochemistry score.

TUNEL staining. TUNEL staining was performed using a TUNEL assay kit (C1086, Beyotime Biotechnology, China) according to the manufacturer's instructions. Fluorescence images were acquired using a microscope (Olympus DP80, Japan). Positively stained cells were quantified using ImageJ software.

Hematoxylin and eosin staining. Mouse livers and subcutaneous tumors were fixed overnight in a 4% paraformaldehyde and processed for paraffin embedding according to standard protocols. Tissue sections were dewaxed with xylene and rehydrated through a graded ethanol series. Tissue sections were stained with hematoxylin and eosin using standard protocols.

Transwell assays. HCC cells were starved overnight, detached with trypsin, and resuspended in serum-free medium (SFM). Cells (5×10^4) were diluted in 200 μL SFM and were placed in the upper chamber of a transwell insert (8 μm pore size, Corning), with 800 μL cultured medium in the lower chamber. After indicated times, non-migrated cells were removed. Migrated cells were fixed with methanol, stained with crystal violet (1%). Migrated cell numbers were counted under a Nikon ECLIPSE 90i microscope (Japan).

Cell proliferation assays. For the cell proliferation assay, cells were seeded in quintuplicate in a 96-well plate (2,000 cells/well) and were incubated at 37 °C with 5% CO_2 for 72 h. Every 24 h, cell intensity was assessed using the Cell Counting Kit-8 (CCK-8; Beyotime Biotech, C0038). Briefly, 10 μl of CCK-8 solution was added to each well and incubated for 1h at 37°C, followed by

recording results using microplate reader (BMG LABTECH) to measure the absorbance of CCK-8 at 450 nm. The highest and lowest values were excluded from the analysis.

IC50 measurement. HCC cells (3000 cells) were plated in 96-well plates and treated with various concentrations of sorafenib. After 48 h, CCK-8 (10 μL) was added to incubate for 45 min at 37°C. The absorbance was measured at 450 nm using a microplate reader. The value of IC50 was calculated using GraphPad Prism 8 software.

Immunoprecipitation. HCC and HEK293 cells, transfected with the indicated plasmids, were lysed in buffer (50 mM Tris-HCl, pH 7.4; 150 mM NaCl; 1 mM EDTA; and 1% NP-40) supplemented with protease inhibitors. One milligram of total protein were incubated overnight at 4°C with anti-Flag (M2, Sigma-Aldrich, M8823) or anti-HA magnetic beads (Thermo Scientific, 88837) to pull down the corresponding tagged proteins. For co-immunoprecipitation, anti-ANPEP or anti-SLC3A2 antibodies were pre-incubated with protein A/G magnetic beads (Pierce, Rockford, IL, USA) for 4 h. Normal rabbit IgG (Proteintech Group, SA00001-2) and normal mouse IgG (Proteintech Group, SA00001-1) were used as controls. After overnight incubation on a rotator at 4° C, the beads were washed four times with lysis buffer. Proteins were eluted by adding 100 μL of 1 ×Laemmli buffer and boiling at 100°C for 10 min, followed by Western Blot analysis. Primary antibodies used for immunoprecipitation are listed in Supplemental Table 8.

Immunofluorescence. Cells were transfected with the indicated vectors and cultured under specified conditions. These cells were fixed with 4% paraformaldehyde (PFA) and permeabilized with 0.1% Triton X-100. After blocking with 10% BSA for 30 min at room temperature, cells were incubated with primary antibodies overnight at 4°C. After washing with PBS, cells were incubated with secondary antibodies for 1 h at room temperature. Nuclei were counterstained with DAPI

(Beyotime Biotech, P0131). Fluorescence images were acquired using a microscope.
