# Supplementary material for: Chronic stress–induced ANPEP drives liver cancer progression by increasing glutathione synthesis and inhibiting ferroptosis
Source: J Clin Invest. 2025 Dec 2;136(4):e195685. doi: 10.1172/JCI195685 (PMC12904706; doi:10.1172/JCI195685)

Figure2.E

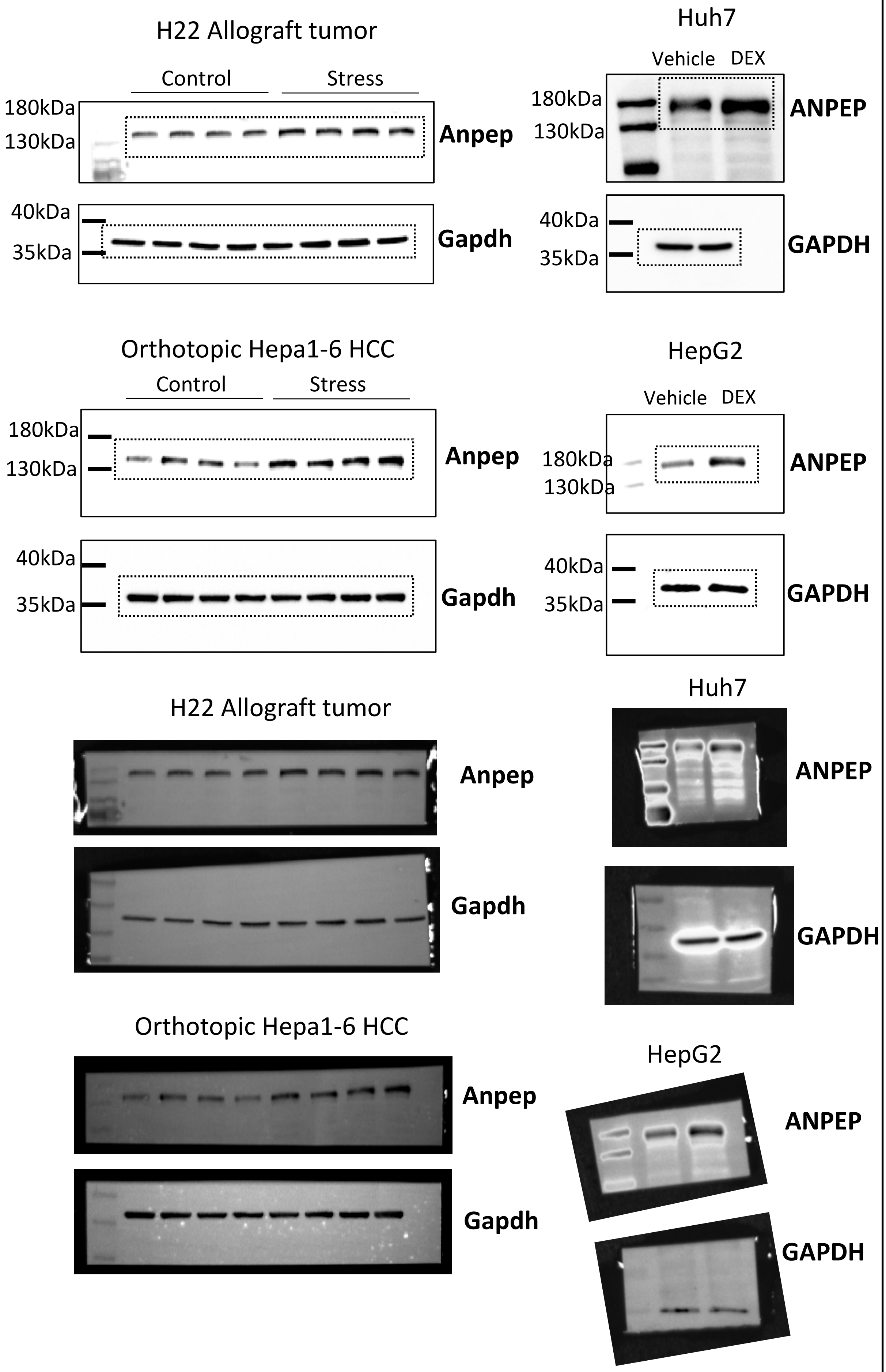
H22 Allograft tumor
Control
Stress
180kDa
130kDa
Anpep
40kDa
35kDa
Gapdh
Huh7
Vehicle
DEX
180kDa
130kDa
ANPEP
40kDa
35kDa
GAPDH
Orthotopic Hepa1-6 HCC
Control
Stress
180kDa
130kDa
Anpep
40kDa
35kDa
Gapdh
HepG2
Vehicle
DEX
180kDa
130kDa
ANPEP
40kDa
35kDa
GAPDH
H22 Allograft tumor
Anpep
Gapdh
Huh7
ANPEP
GAPDH
Orthotopic Hepa1-6 HCC
Anpep
Gapdh
HepG2
ANPEP
GAPDH

Figure 4.B

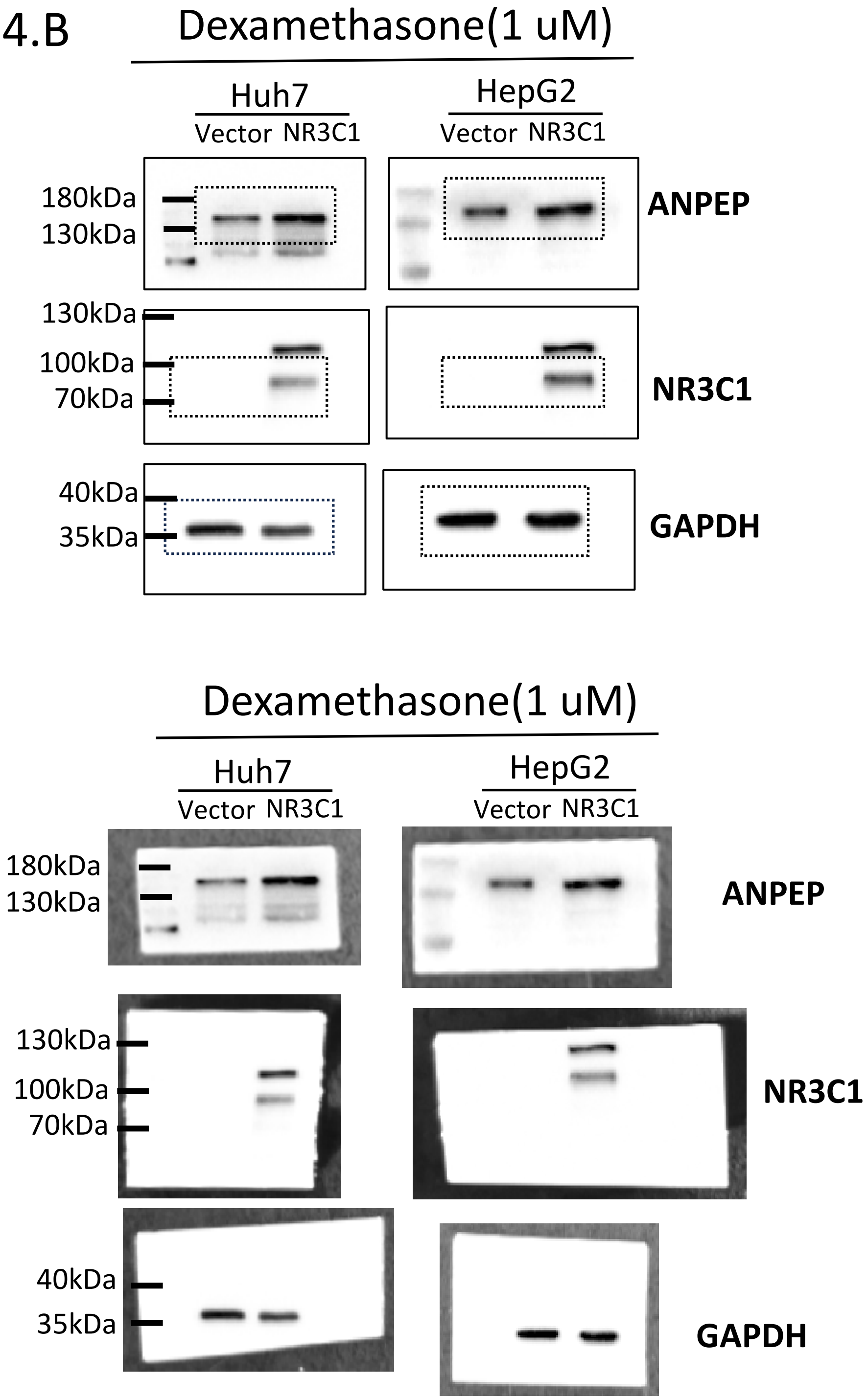

Dexamethasone(1 uM)
Huh7
HepG2
Vector NR3C1
Vector NR3C1
180kDa
130kDa
ANPEP
130kDa
100kDa
70kDa
NR3C1
40kDa
35kDa
GAPDH
Dexamethasone(1 uM)
Huh7
HepG2
Vector NR3C1
Vector NR3C1
180kDa
130kDa
ANPEP
130kDa
100kDa
70kDa
NR3C1
40kDa
35kDa
GAPDH

Figure 4.B

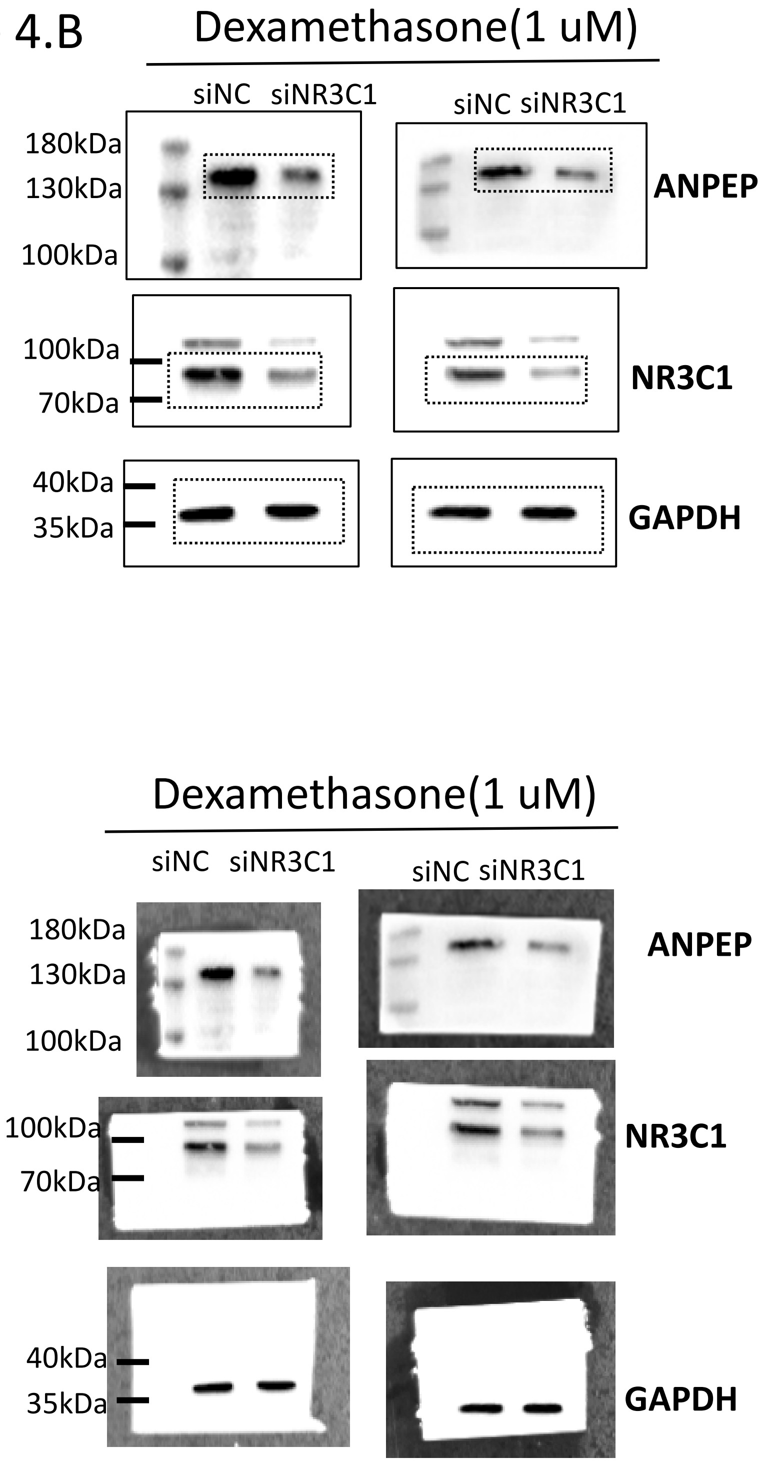

Dexamethasone(1 uM)
siNC
siNR3C1
siNC siNR3C1
180kDa
130kDa
100kDa
ANPEP
100kDa
70kDa
NR3C1
40kDa
35kDa
GAPDH
Dexamethasone(1 uM)
siNC
siNR3C1
siNC siNR3C1
180kDa
130kDa
100kDa
ANPEP
100kDa
70kDa
NR3C1
40kDa
35kDa
GAPDH

Figure 4.D

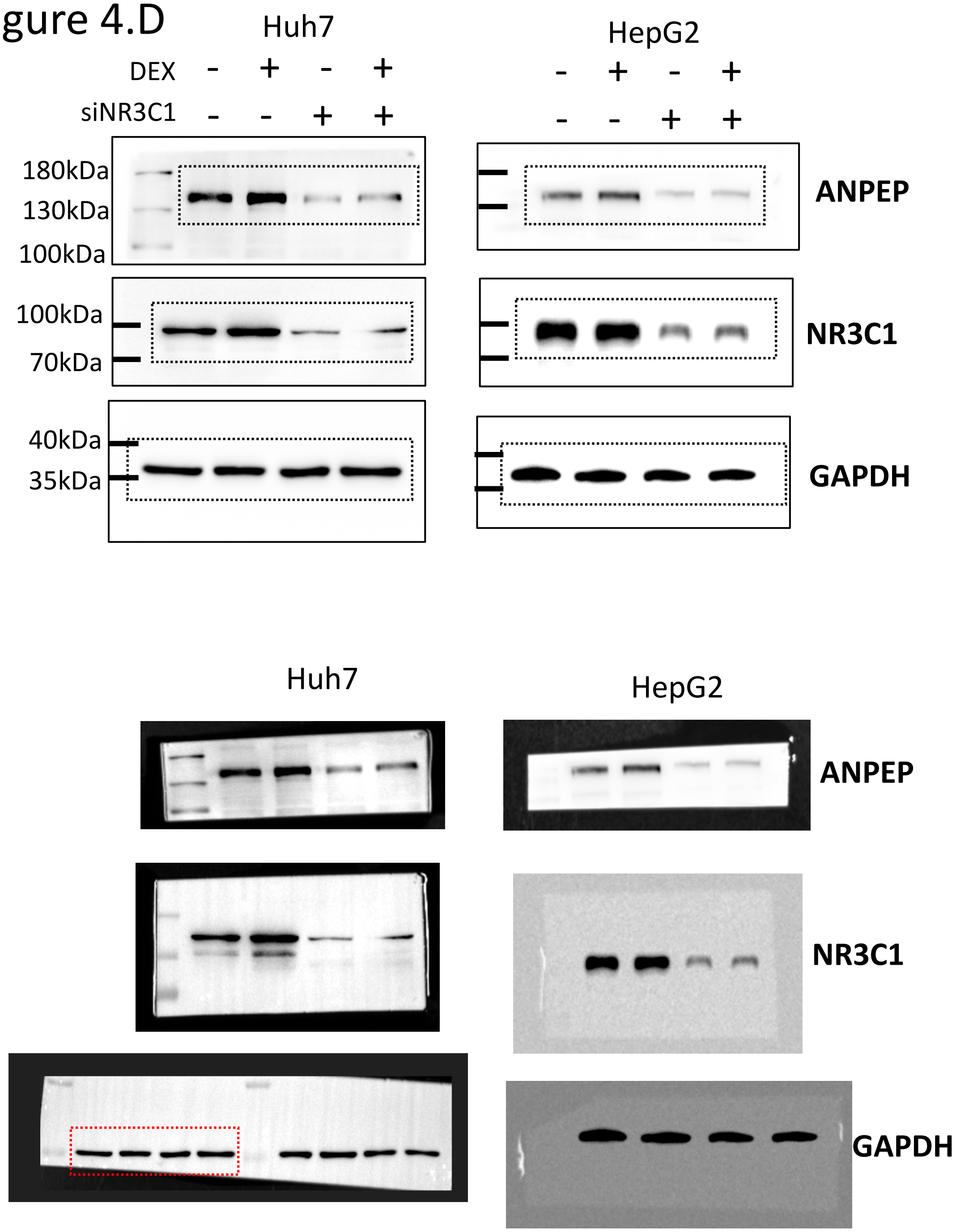

Huh7
HepG2
DEX - + - +
siNR3C1 - - + +
180kDa
130kDa
100kDa
100kDa
70kDa
40kDa
35kDa
ANPEP
NR3C1
GAPDH
Huh7
HepG2
ANPEP
NR3C1
GAPDH

Figure 4.H

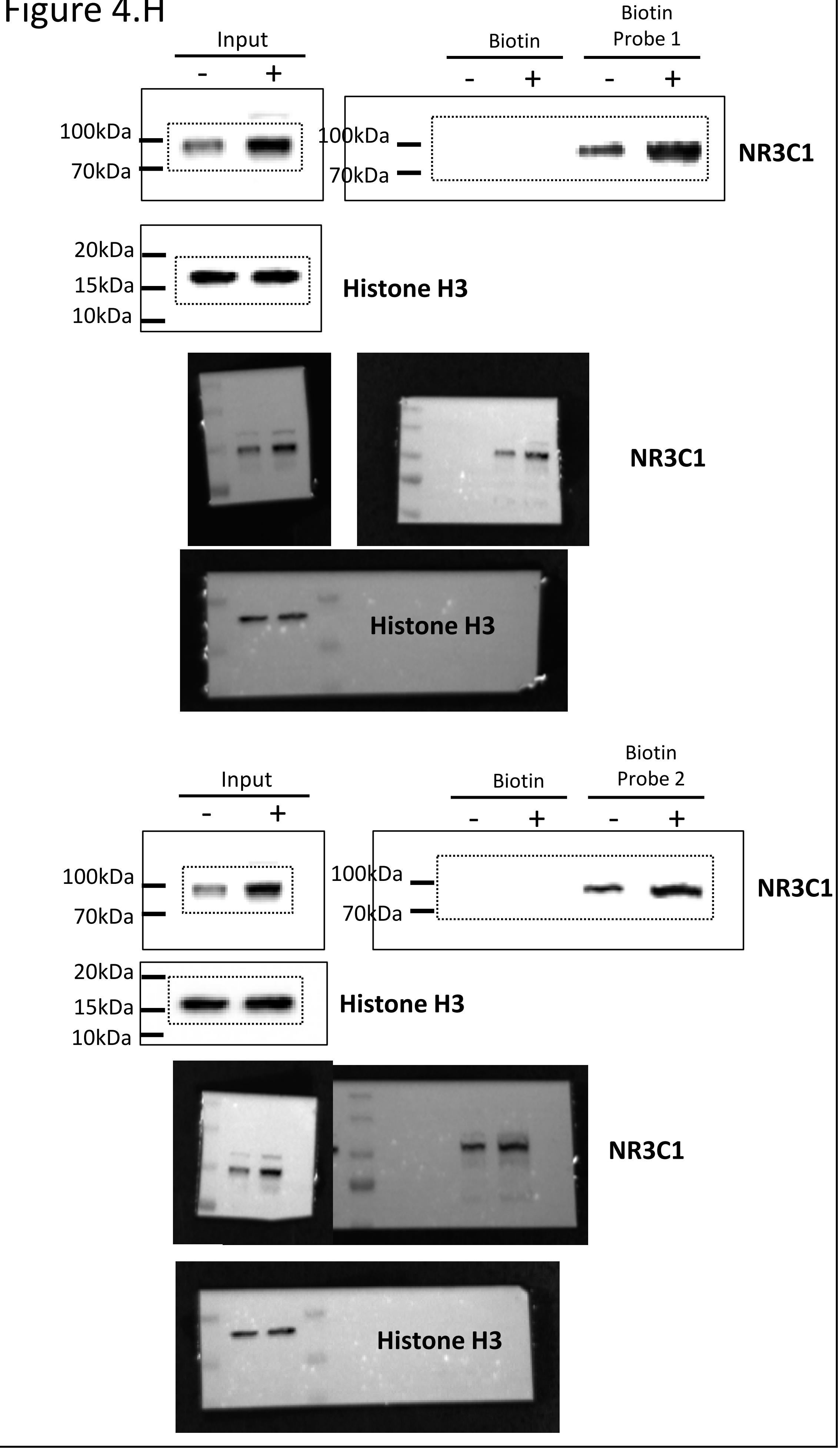
Input
-
+
Biotin
Biotin Probe 1
100kDa
70kDa
NR3C1
20kDa
15kDa
10kDa
Histone H3
NR3C1
Histone H3
Input
Biotin
Biotin Probe 2
100kDa
70kDa
NR3C1
20kDa
15kDa
10kDa
Histone H3
NR3C1
Histone H3

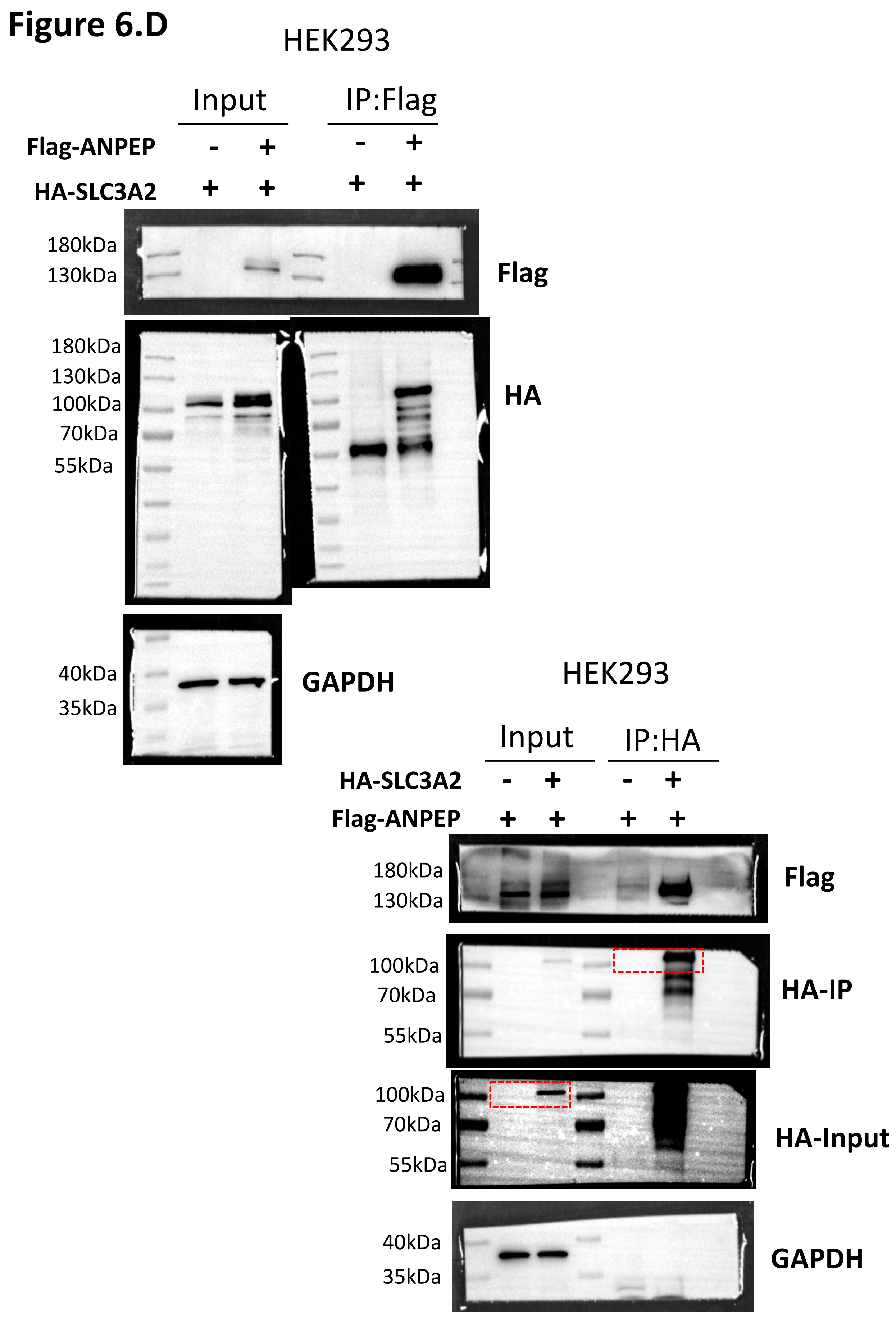
Figure 6.D
HEK293
Input
IP:Flag
Flag-ANPEP - + - +
HA-SLC3A2 + + + +
180kDa
130kDa
Flag
180kDa
130kDa
100kDa
70kDa
55kDa
HA
40kDa
35kDa
GAPDH
HEK293
Input
IP:HA
HA-SLC3A2 - + - +
Flag-ANPEP + + + +
180kDa
130kDa
Flag
100kDa
70kDa
55kDa
HA-IP
100kDa
70kDa
55kDa
HA-Input
40kDa
35kDa
GAPDH

Figure 6.E

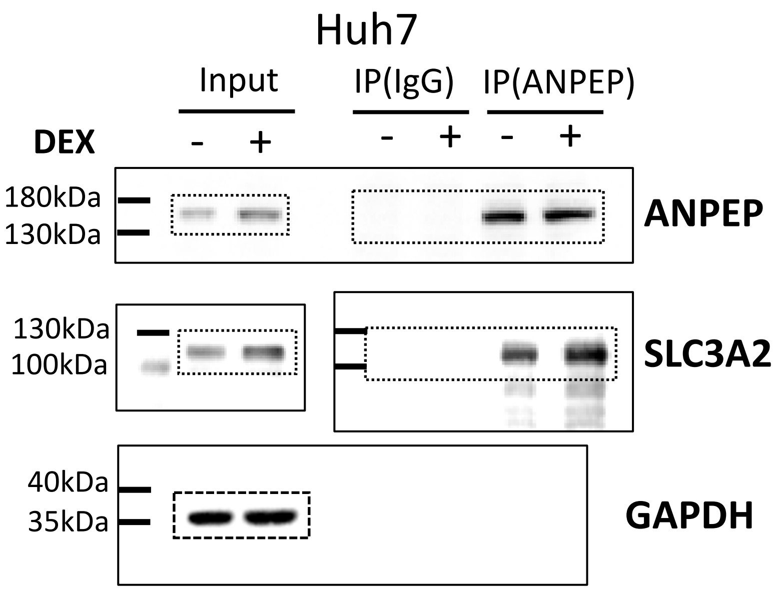

Huh7
Input
IP(IgG)
IP(ANPEP)
DEX
- + - + - +
180kDa
130kDa
ANPEP
130kDa
100kDa
SLC3A2
40kDa
35kDa
GAPDH

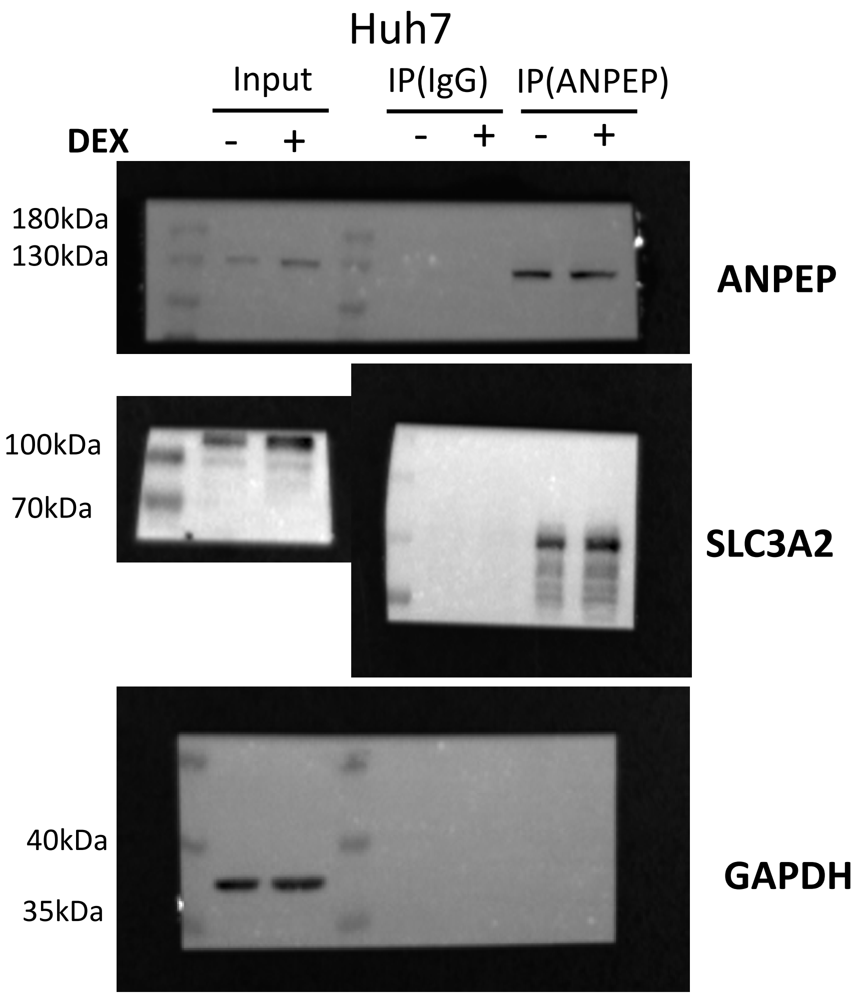

Huh7
Input
IP(IgG)
IP(ANPEP)
DEX
- + - + - +
180kDa
130kDa
ANPEP
100kDa
70kDa
SLC3A2
40kDa
35kDa
GAPDH

Figure 8.A

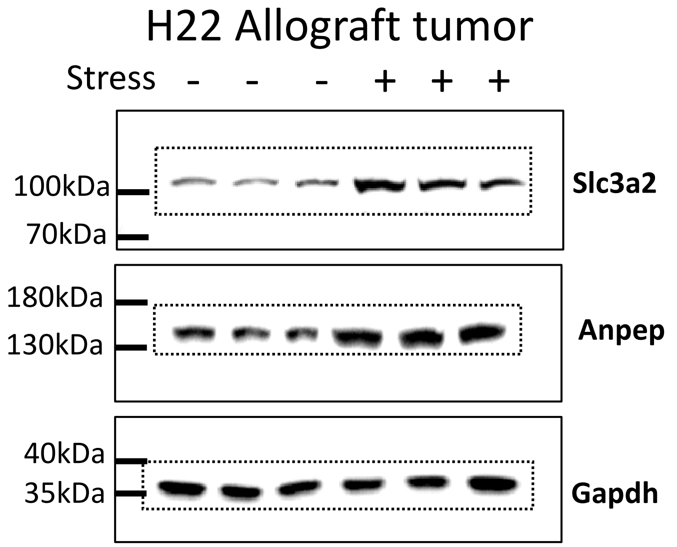

H22 Allograft tumor
Stress - - - + + +
100kDa
70kDa
Slc3a2
180kDa
130kDa
Anpep
40kDa
35kDa
Gapdh

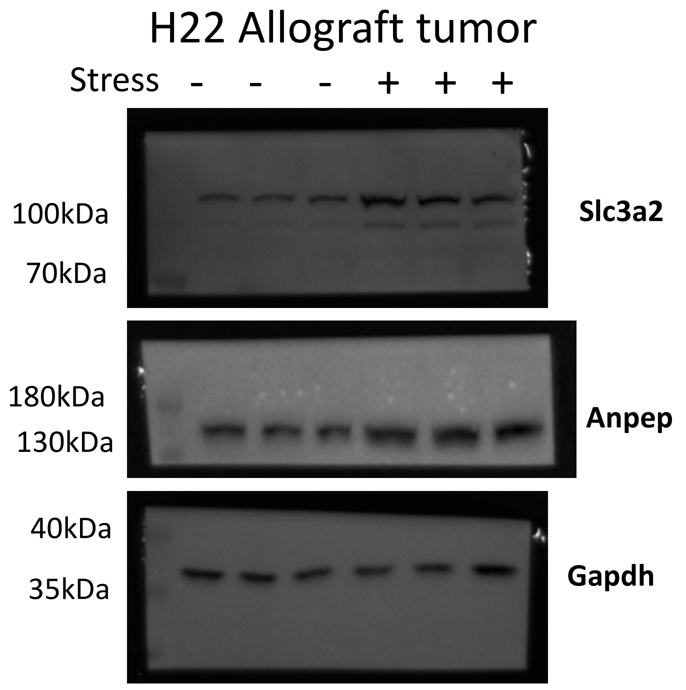

H22 Allograft tumor
Stress - - - + + +
100kDa
70kDa
Slc3a2
180kDa
130kDa
Anpep
40kDa
35kDa
Gapdh

Figure 8.B

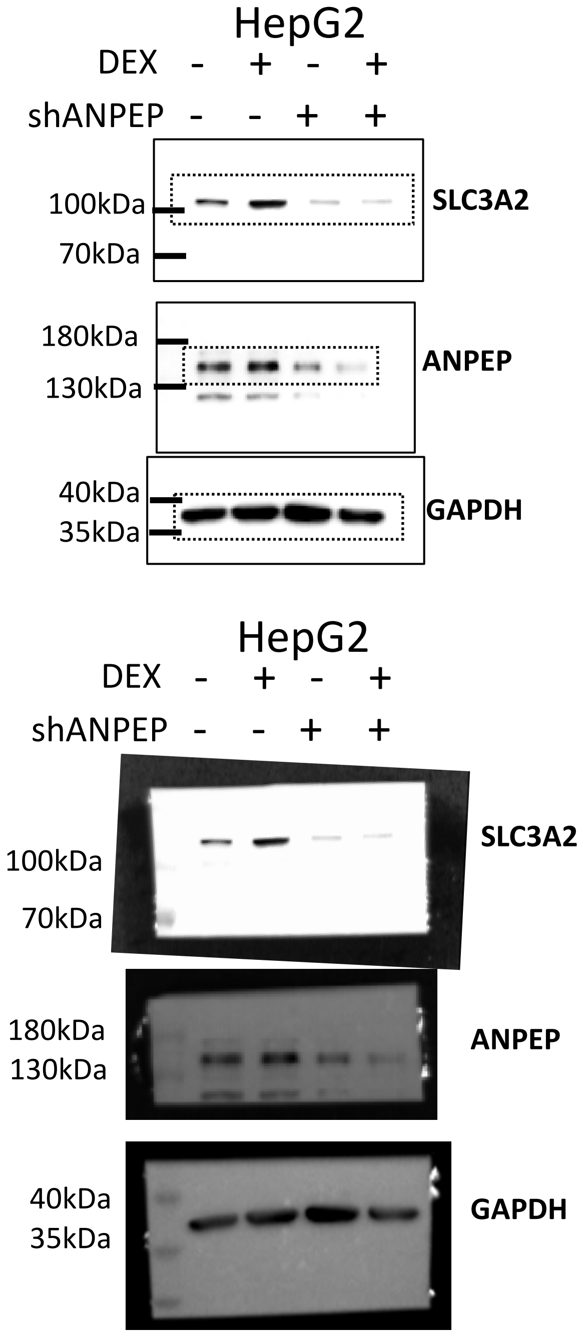
HepG2
DEX - + - +
shANPEP - - + +
100kDa
70kDa
SLC3A2
180kDa
130kDa
ANPEP
40kDa
35kDa
GAPDH
HepG2
DEX - + - +
shANPEP - - + +
100kDa
70kDa
SLC3A2
180kDa
130kDa
ANPEP
40kDa
35kDa
GAPDH

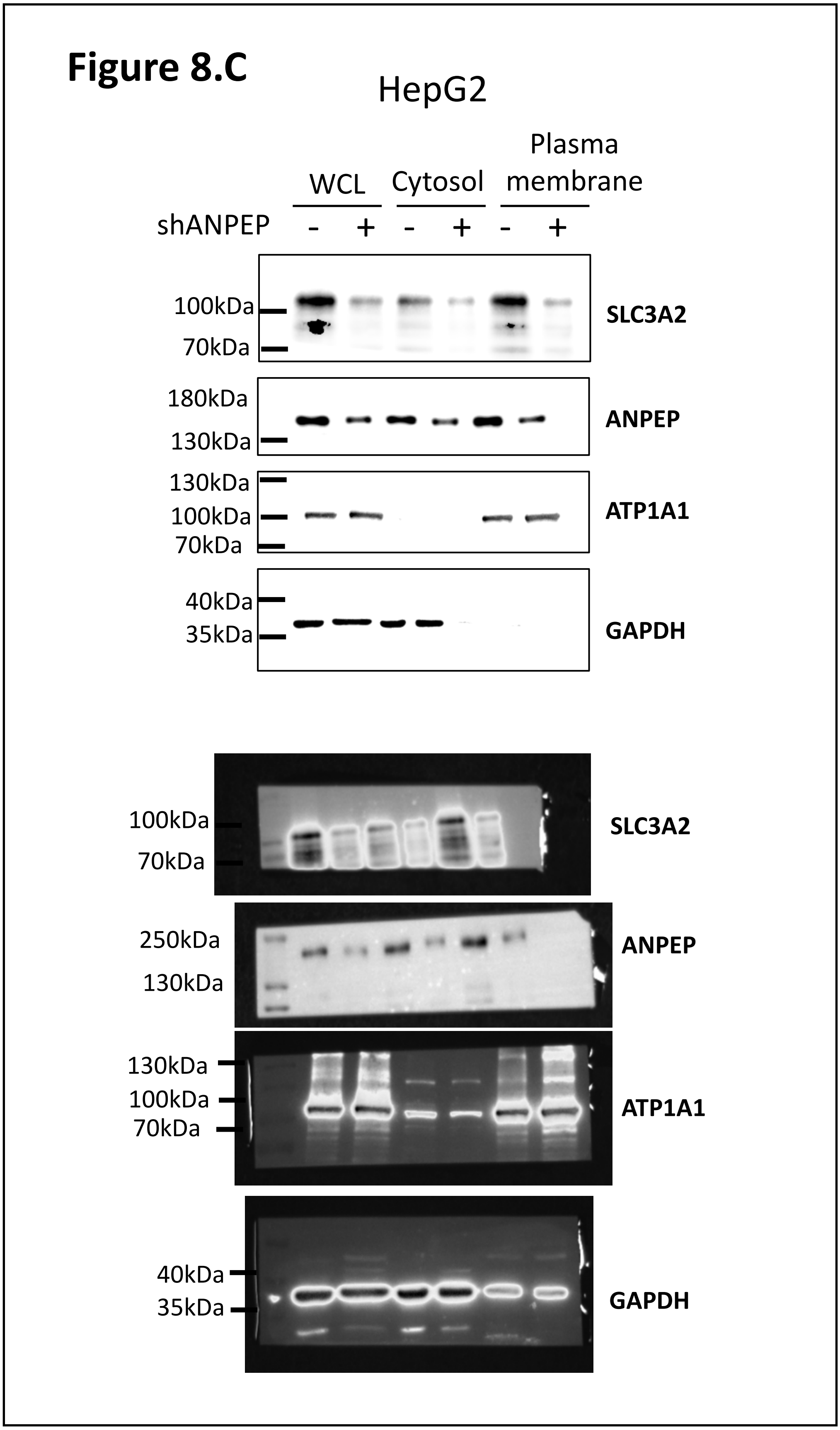
Figure 8.C
HepG2
WCL
Cytosol
Plasma membrane
shANPEP
- + - + - +
100kDa
70kDa
SLC3A2
180kDa
130kDa
ANPEP
130kDa
100kDa
70kDa
ATP1A1
40kDa
35kDa
GAPDH
100kDa
70kDa
SLC3A2
250kDa
130kDa
ANPEP
130kDa
100kDa
70kDa
ATP1A1
40kDa
35kDa
GAPDH

Figure 8.C

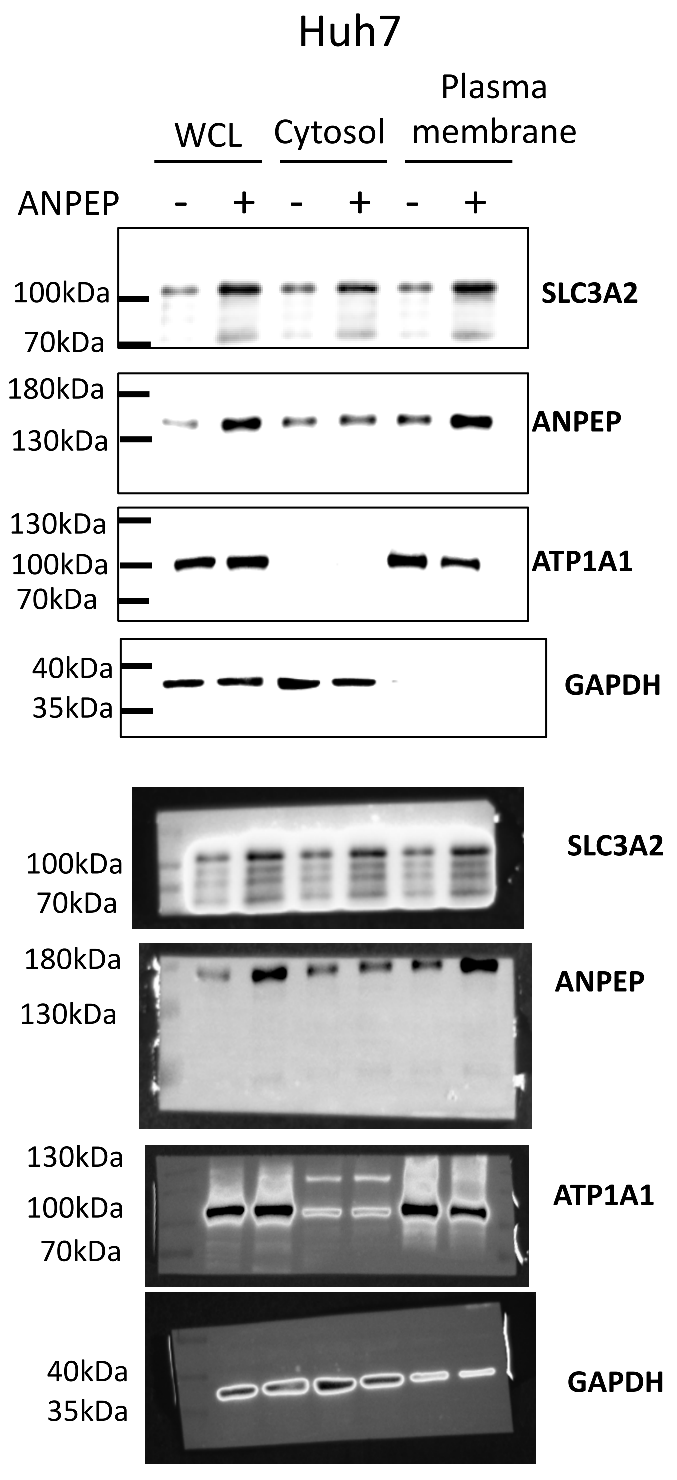
Huh7
WCL
Cytosol
Plasma membrane
ANPEP
- + - + - +
100kDa
70kDa
SLC3A2
180kDa
130kDa
ANPEP
130kDa
100kDa
70kDa
ATP1A1
40kDa
35kDa
GAPDH
100kDa
70kDa
SLC3A2
180kDa
130kDa
ANPEP
130kDa
100kDa
70kDa
ATP1A1
40kDa
35kDa
GAPDH

Figure 8.E

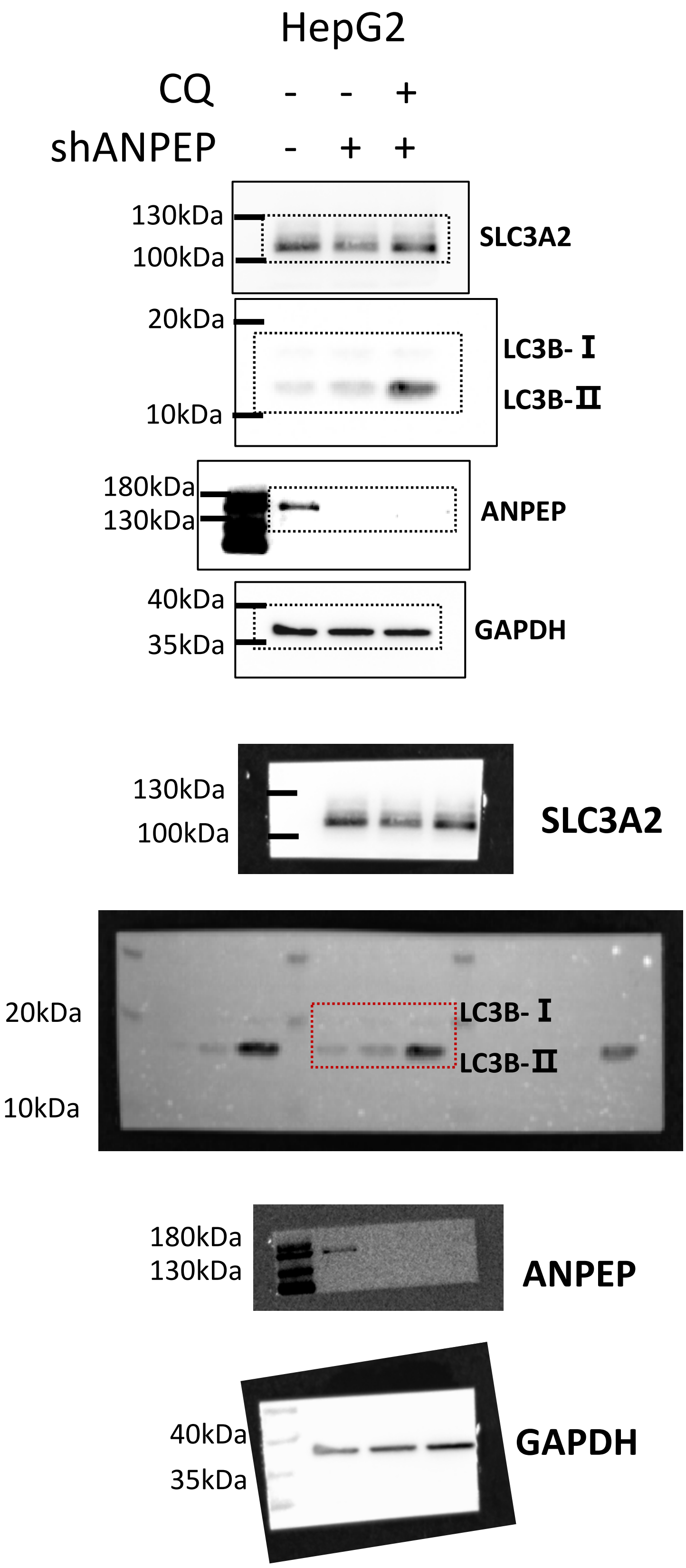

HepG2
CQ - - +
shANPEP - + +
130kDa
100kDa
SLC3A2
20kDa
10kDa
LC3B- Ⅰ
LC3B-Ⅱ
180kDa
130kDa
ANPEP
40kDa
35kDa
GAPDH
130kDa
100kDa
SLC3A2
20kDa
10kDa
LC3B- Ⅰ
LC3B-Ⅱ
180kDa
130kDa
ANPEP
40kDa
35kDa
GAPDH

Figure 8.G

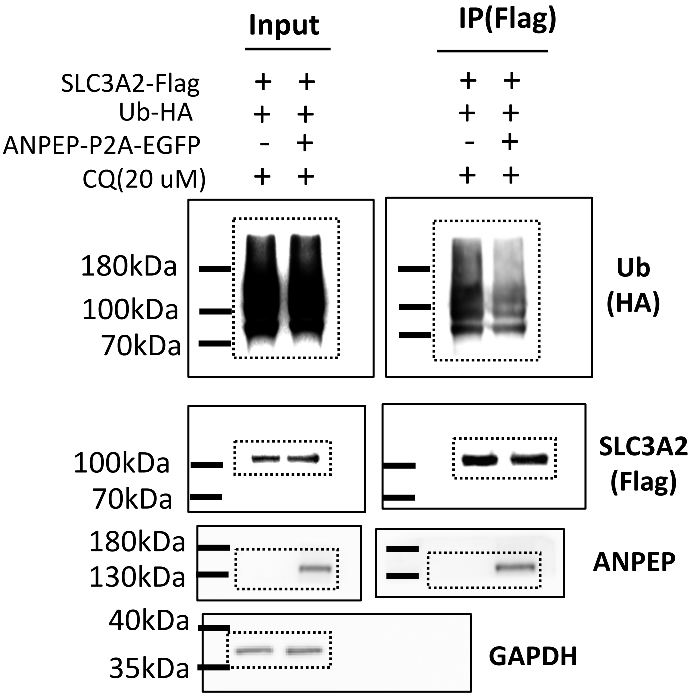
Input
IP(Flag)
SLC3A2-Flag + + + +
Ub-HA + + + +
ANPEP-P2A-EGFP - + - +
CQ(20 uM) + + + +
180kDa
100kDa
70kDa
Ub
(HA)
100kDa
70kDa
SLC3A2
(Flag)
180kDa
130kDa
ANPEP
40kDa
35kDa
GAPDH

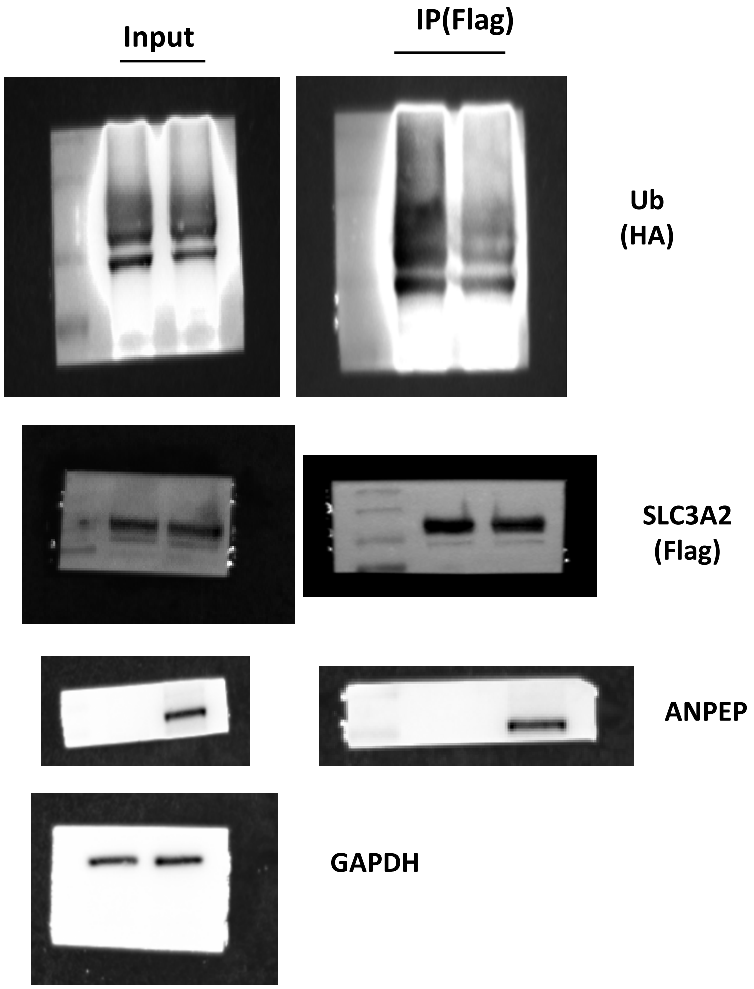
Input
IP(Flag)
Ub
(HA)
SLC3A2
(Flag)
ANPEP
GAPDH

Figure 8.H

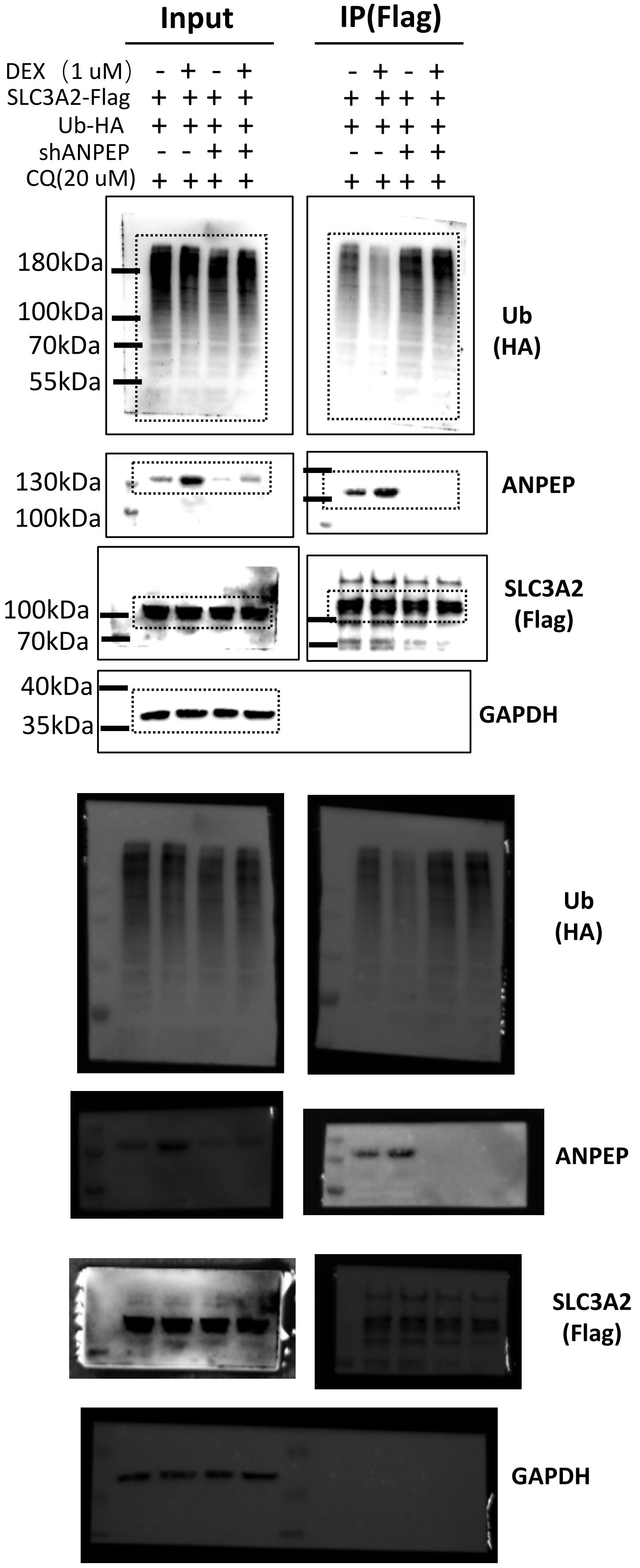
Input
IP(Flag)
DEX（1 uM） - + - + - + - +
SLC3A2-Flag + + + + + + + +
Ub-HA + + + + + + + +
shANPEP - - + + - - + +
CQ(20 uM) + + + + + + + +
180kDa
100kDa
70kDa
55kDa
Ub
(HA)
130kDa
100kDa
ANPEP
100kDa
70kDa
SLC3A2
(Flag)
40kDa
35kDa
GAPDH
Ub
(HA)
ANPEP
SLC3A2
(Flag)
GAPDH

Figure 8.K

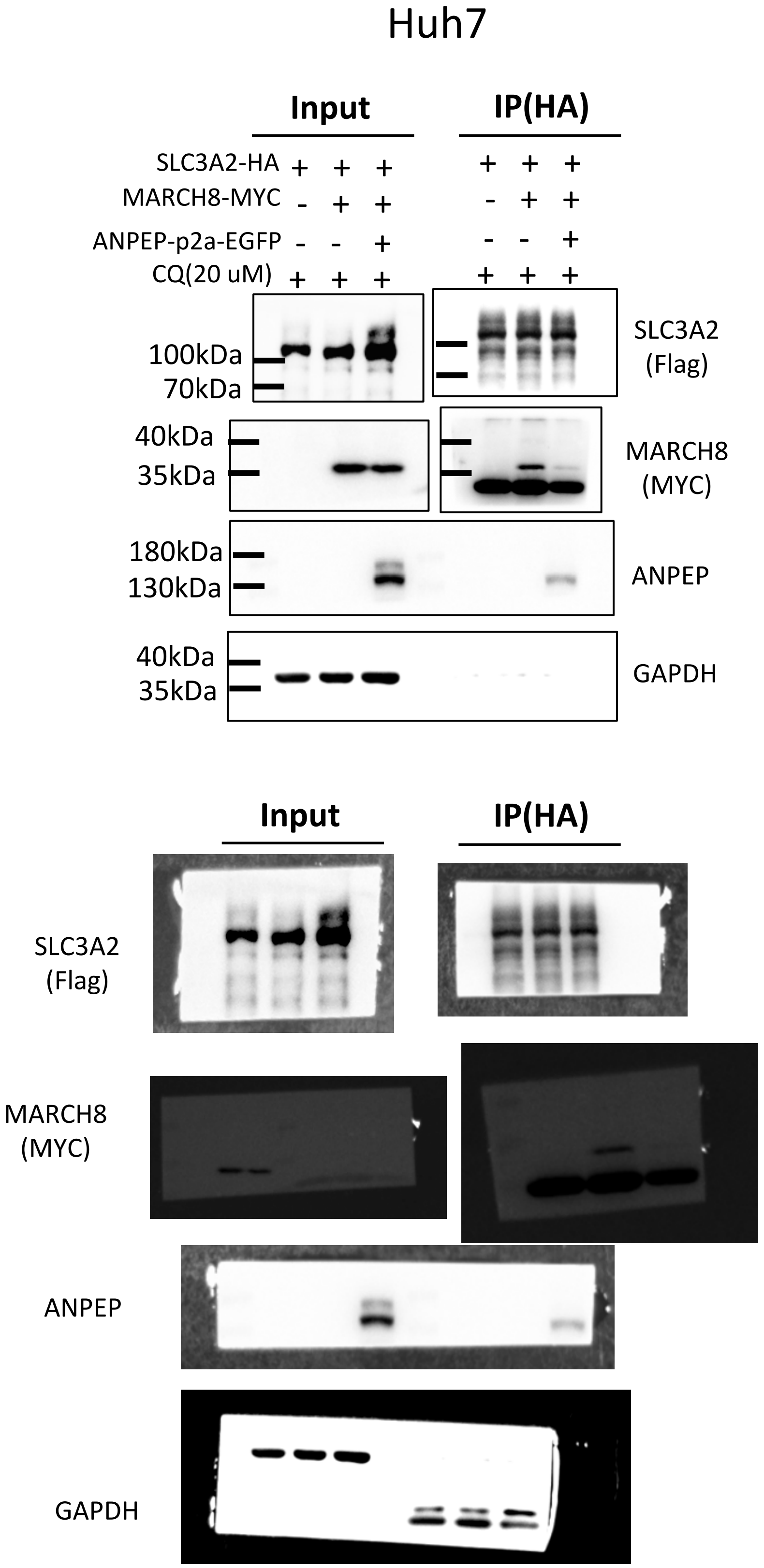
Huh7
Input
IP(HA)
SLC3A2-HA + + + + + +
MARCH8-MYC - + + - + +
ANPEP-p2a-EGFP - - + - - +
CQ(20 uM) + + + + + +
100kDa
70kDa
SLC3A2 (Flag)
40kDa
35kDa
MARCH8 (MYC)
180kDa
130kDa
ANPEP
40kDa
35kDa
GAPDH
Input
IP(HA)
SLC3A2 (Flag)
MARCH8 (MYC)
ANPEP
GAPDH

Figure 8.K

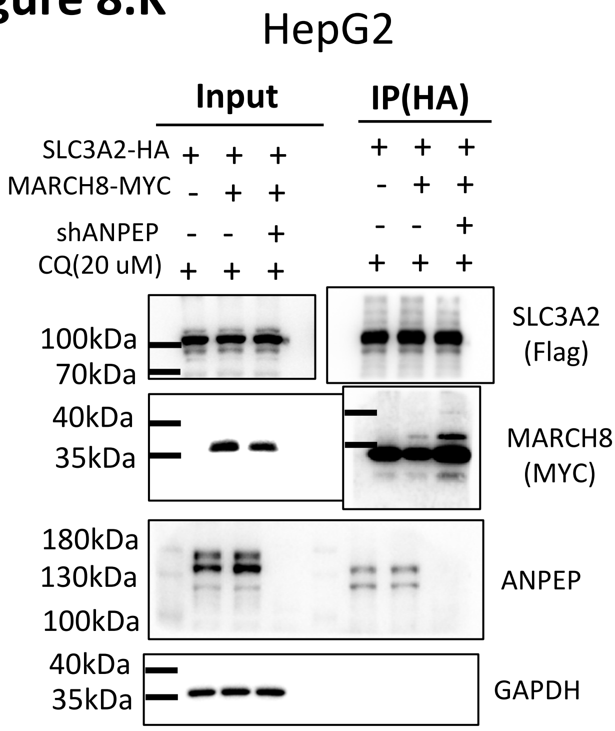

HepG2
Input
IP(HA)
SLC3A2-HA + + + + + +
MARCH8-MYC - + + - + +
shANPEP - - + - - +
CQ(20 uM) + + + + + +
100kDa
70kDa
SLC3A2 (Flag)
40kDa
35kDa
MARCH8 (MYC)
180kDa
130kDa
100kDa
ANPEP
40kDa
35kDa
GAPDH

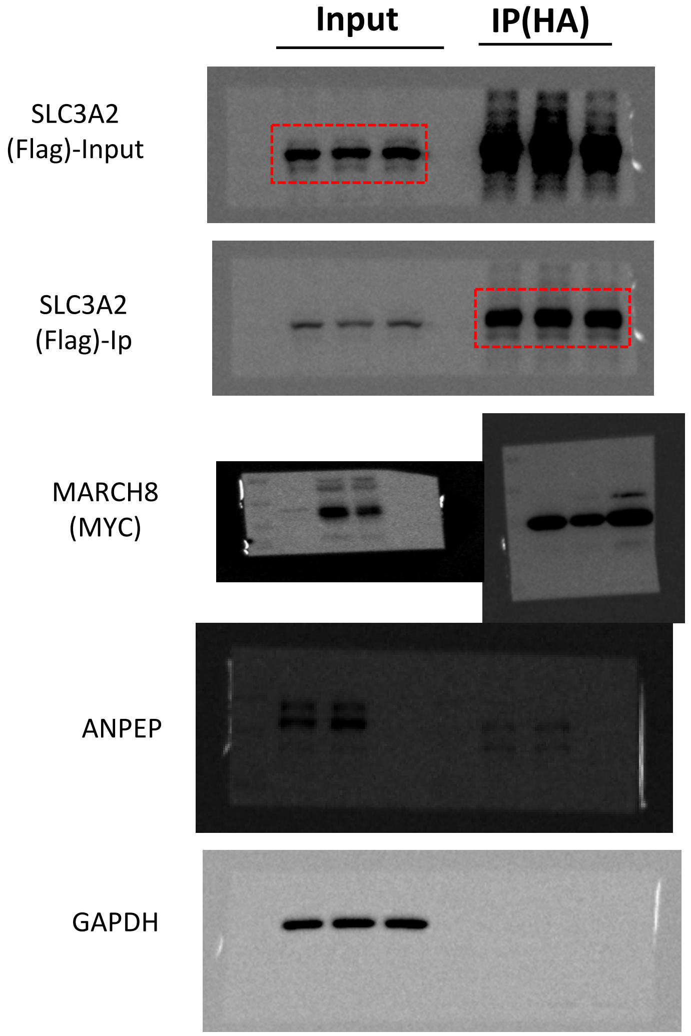

Input
IP(HA)
SLC3A2 (Flag)-Input
SLC3A2 (Flag)-Ip
MARCH8 (MYC)
ANPEP
GAPDH

Figure 8.L

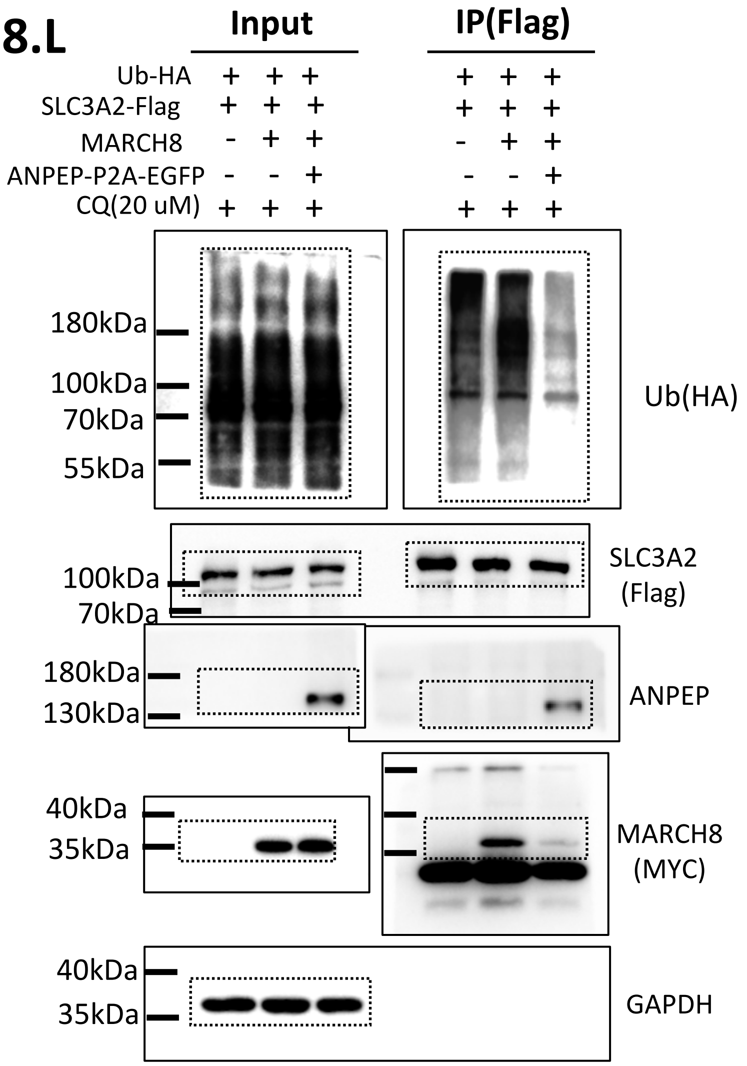
Input
IP(Flag)
Ub-HA
SLC3A2-Flag
MARCH8
ANPEP-P2A-EGFP
CQ(20 uM)
180kDa
100kDa
70kDa
55kDa
Ub(HA)
SLC3A2
(Flag)
180kDa
130kDa
ANPEP
40kDa
35kDa
MARCH8
(MYC)
GAPDH

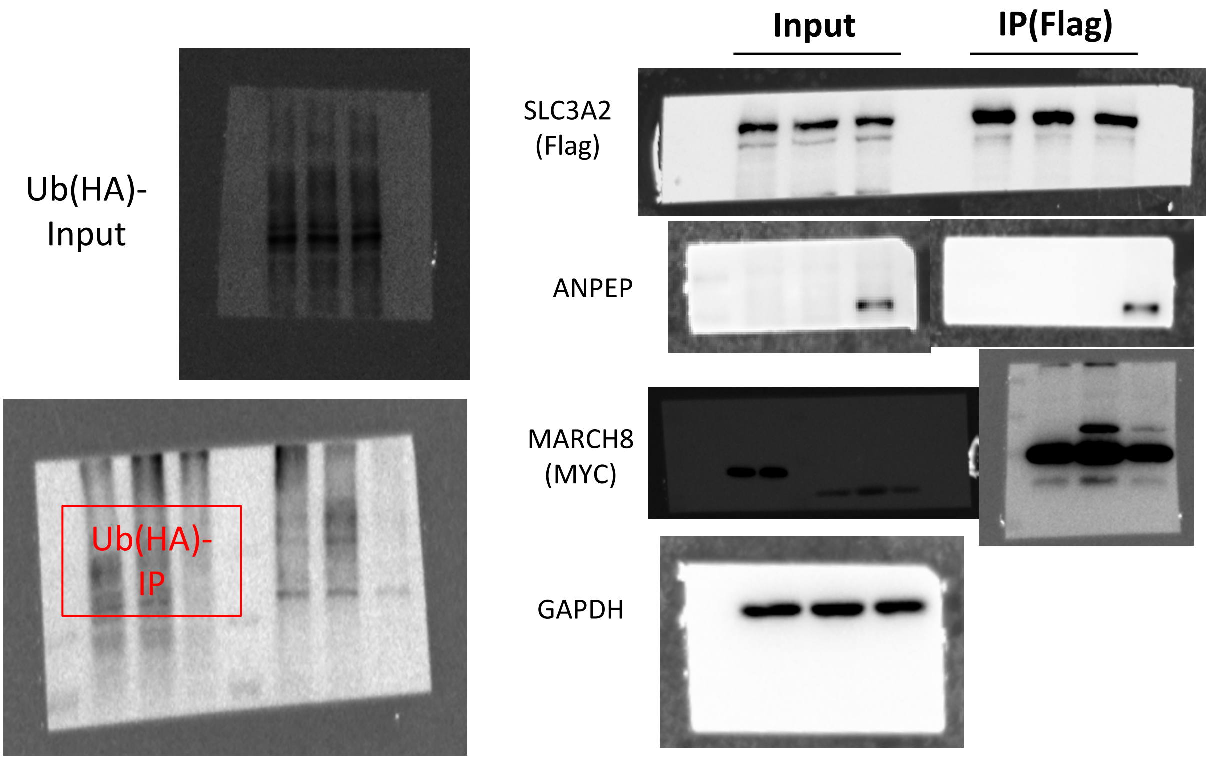
Input
IP(Flag)
Ub(HA)-
Input
Ub(HA)-
IP
SLC3A2
(Flag)
ANPEP
MARCH8
(MYC)
GAPDH

Figure 8.L

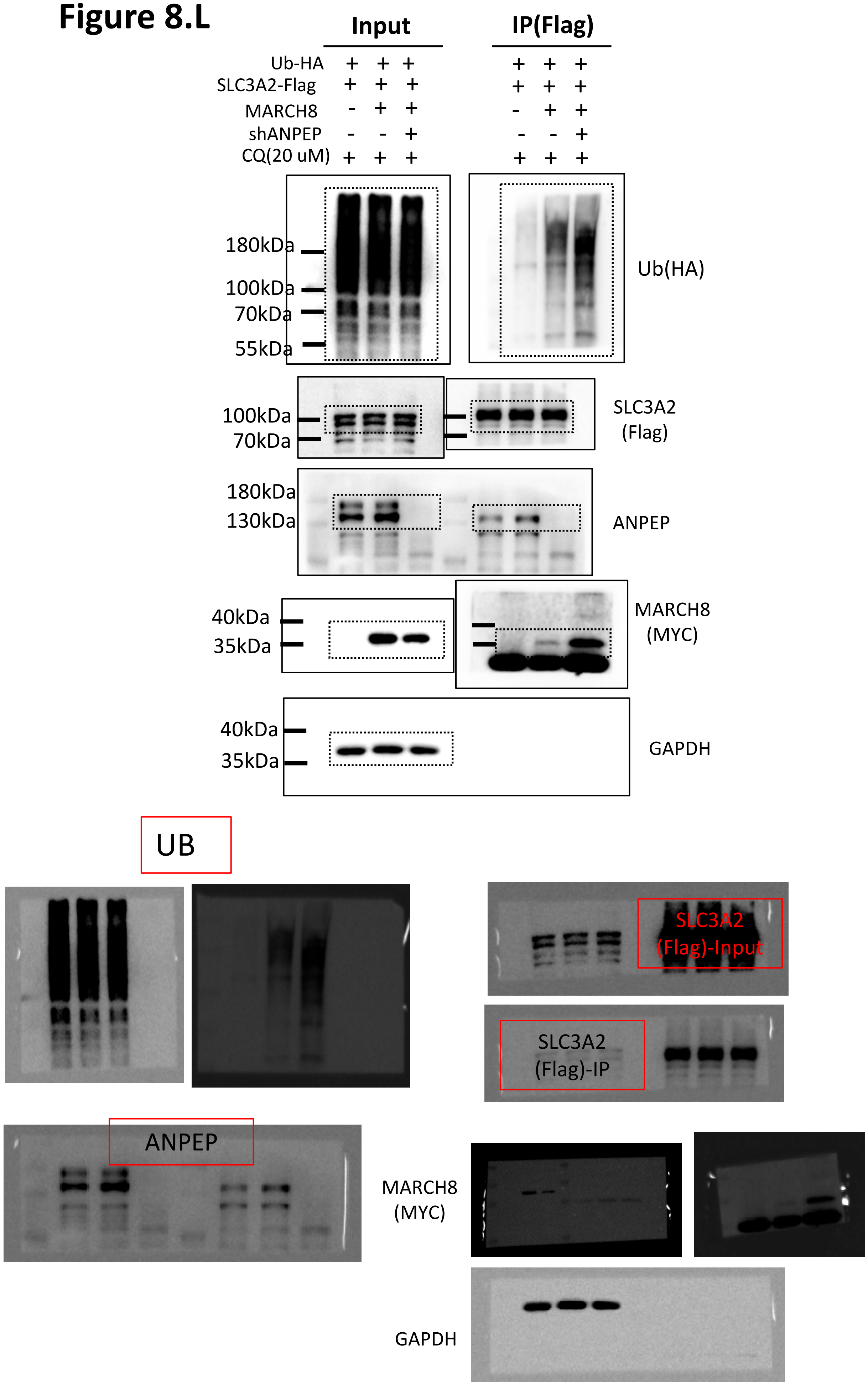

Input
IP(Flag)
Ub-HA + + + + + +
SLC3A2-Flag + + + + + +
MARCH8 - + + - + +
shANPEP - - + - - +
CQ(20 uM) + + + + + +
180kDa
100kDa
70kDa
55kDa
Ub(HA)
100kDa
70kDa
SLC3A2
(Flag)
180kDa
130kDa
ANPEP
40kDa
35kDa
MARCH8
(MYC)
40kDa
35kDa
GAPDH
UB
SLC3A2
(Flag)-Input
SLC3A2
(Flag)-IP
ANPEP
MARCH8
(MYC)
GAPDH

Figure S1.J

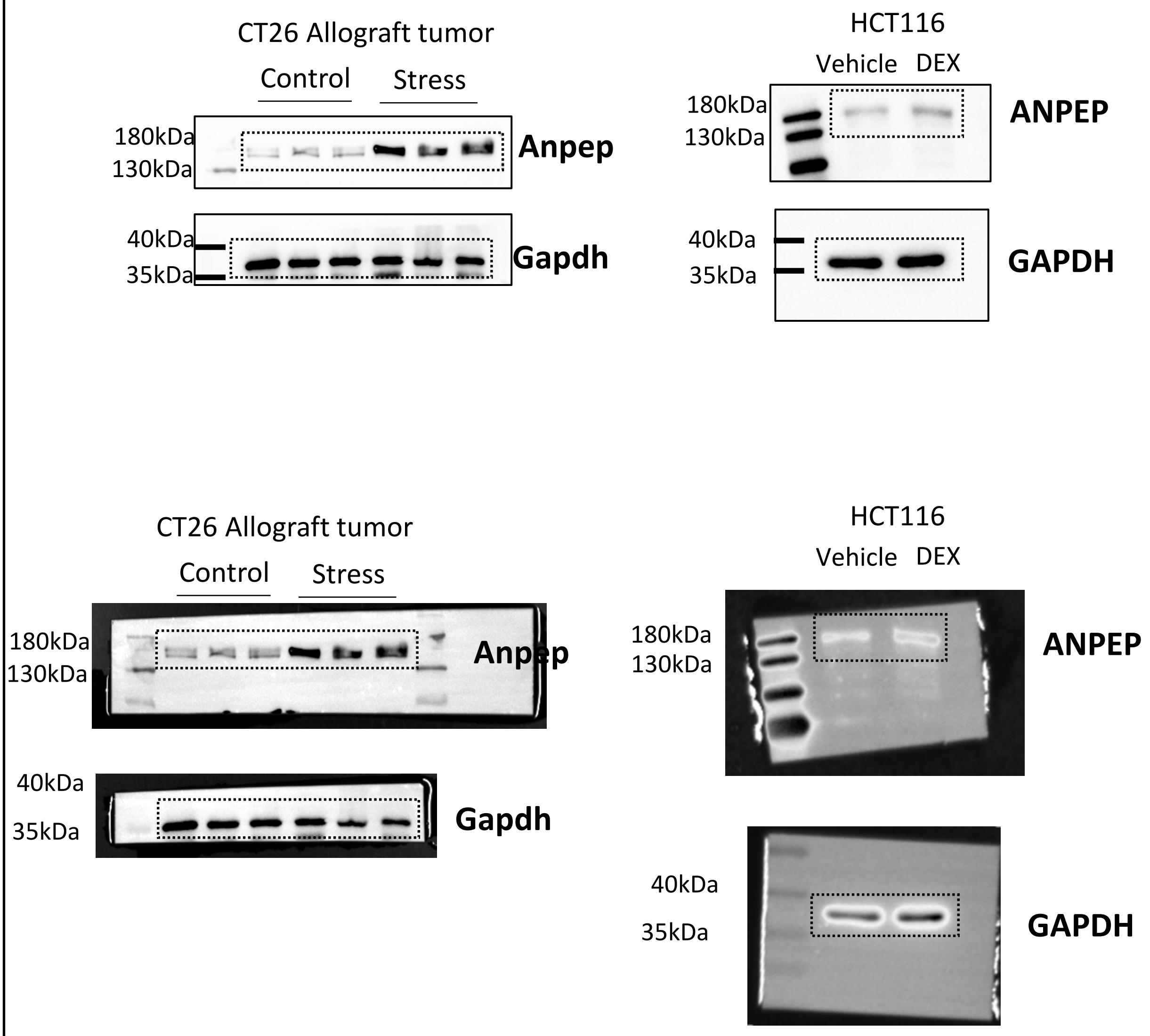

CT26 Allograft tumor
Control
Stress
180kDa
130kDa
Anpep
40kDa
35kDa
Gapdh
HCT116
Vehicle
DEX
180kDa
130kDa
ANPEP
40kDa
35kDa
GAPDH
CT26 Allograft tumor
Control
Stress
180kDa
130kDa
Anpep
40kDa
35kDa
Gapdh
HCT116
Vehicle
DEX
180kDa
130kDa
ANPEP
40kDa
35kDa
GAPDH

Figure S2.C

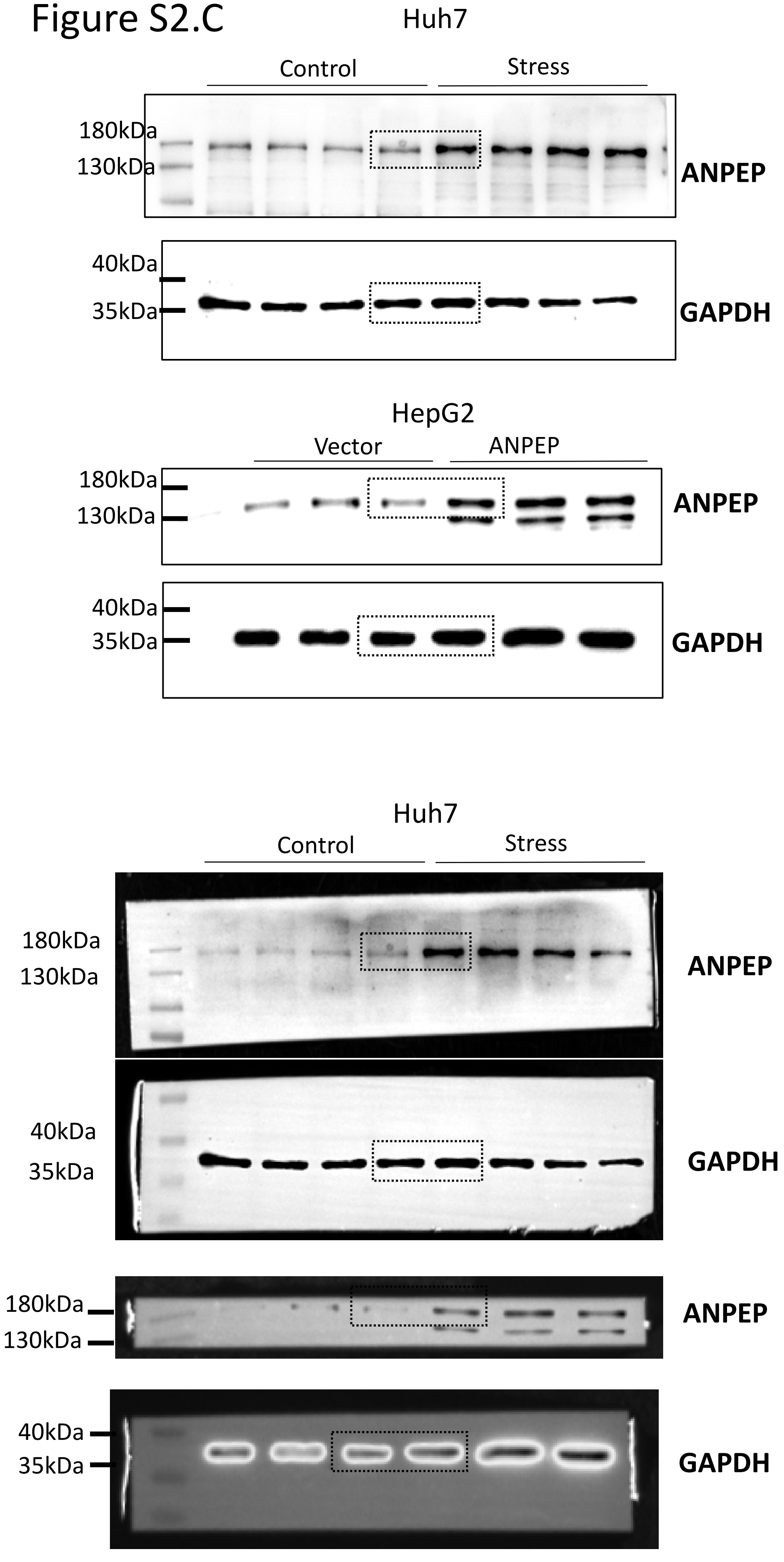
Huh7
Control
Stress
180kDa
130kDa
ANPEP
40kDa
35kDa
GAPDH
HepG2
Vector
ANPEP
180kDa
130kDa
ANPEP
40kDa
35kDa
GAPDH
Huh7
Control
Stress
180kDa
130kDa
ANPEP
40kDa
35kDa
GAPDH
180kDa
130kDa
ANPEP
40kDa
35kDa
GAPDH

Figure S2.D

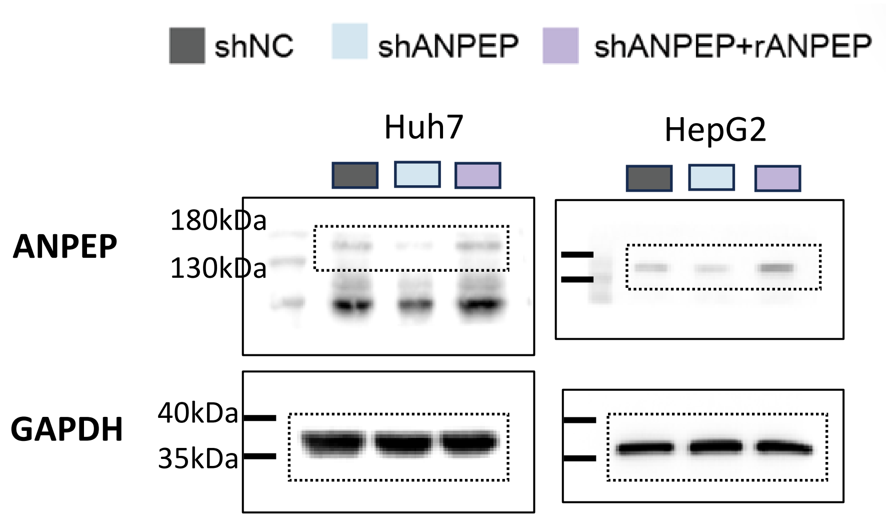

Figure S2.D

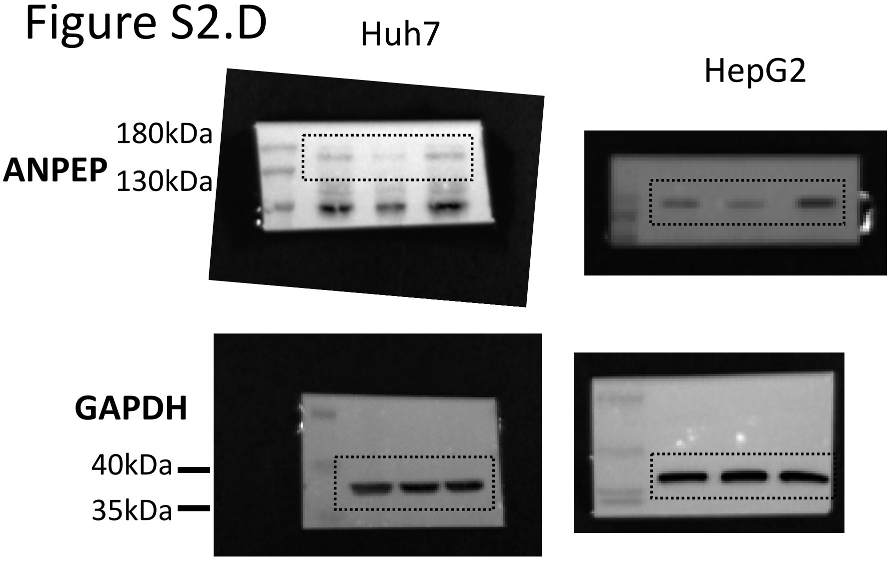

Figure S3.B

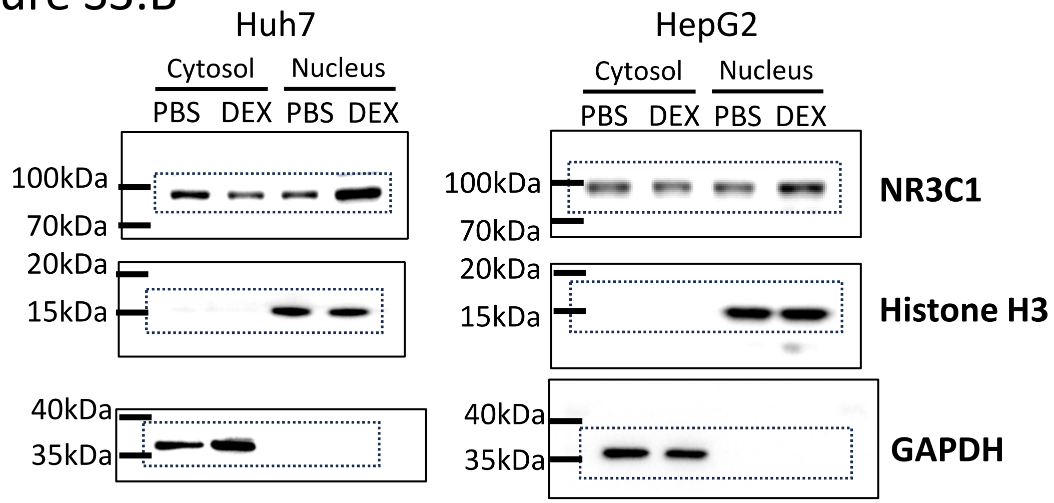

Figure S3.B

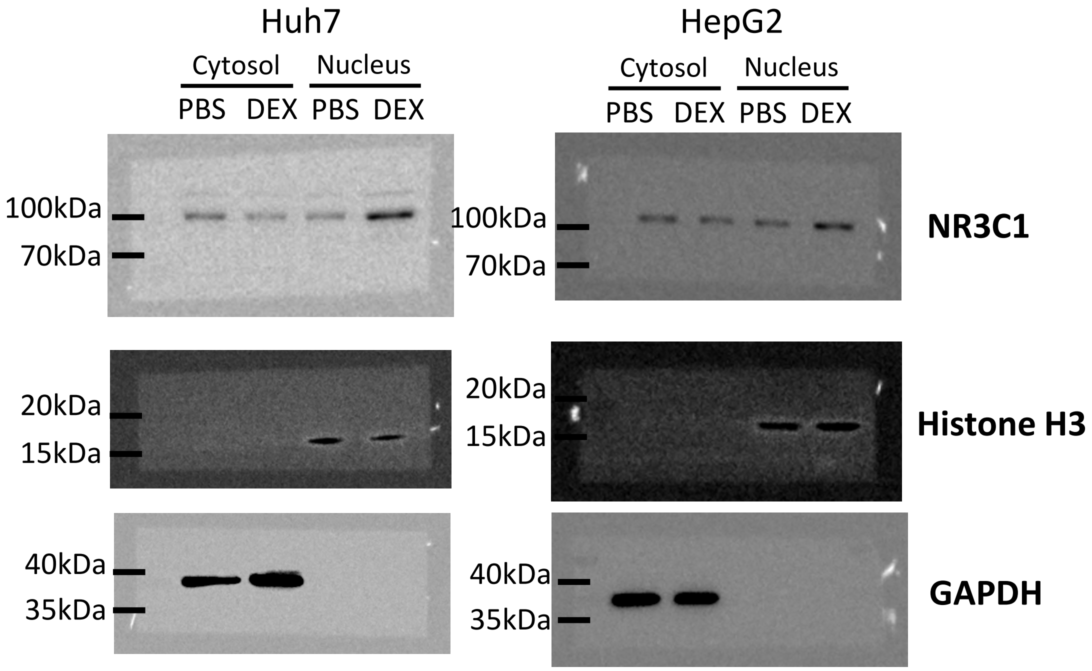

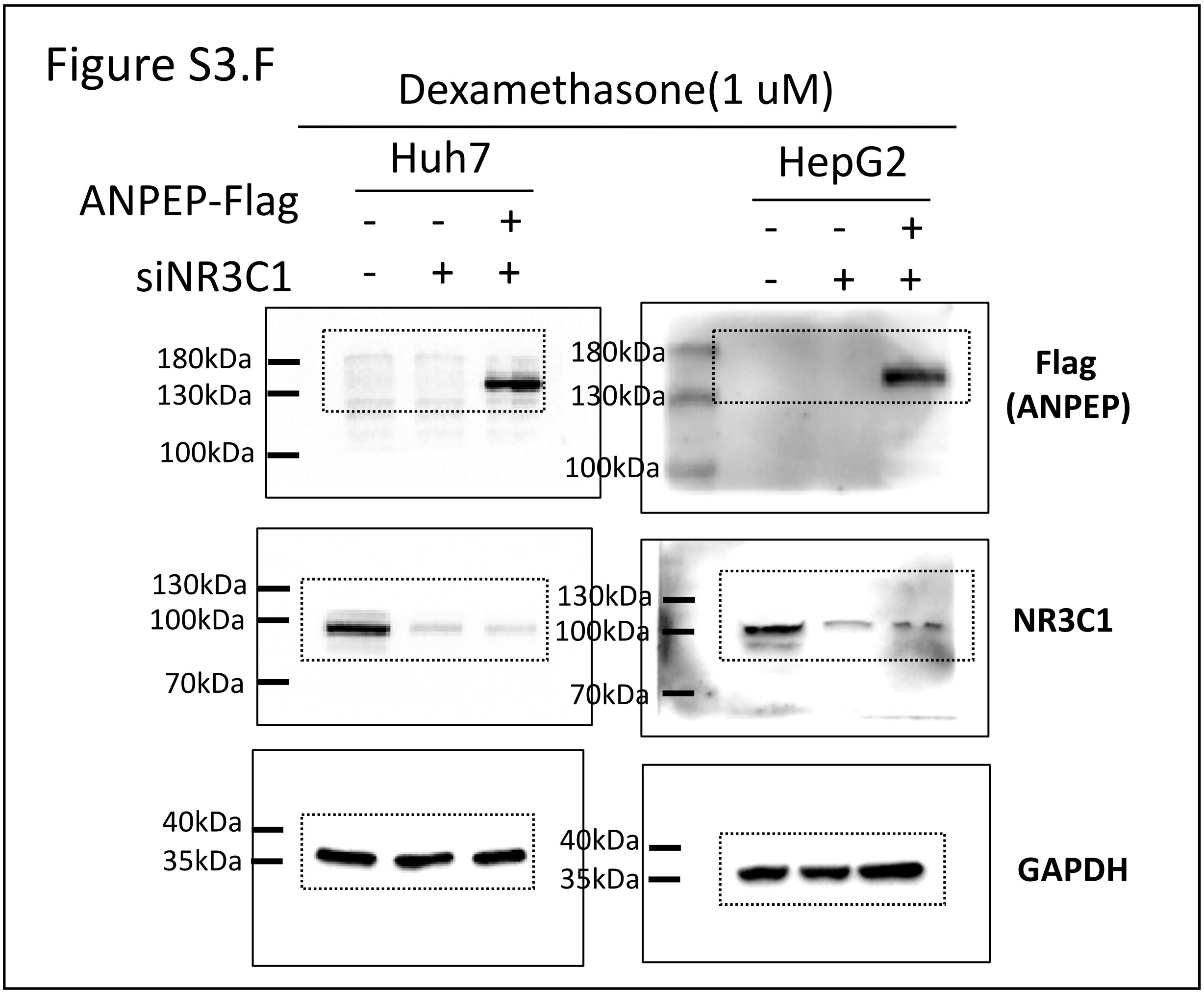
Figure S3.F
Dexamethasone(1 uM)
Huh7
HepG2
ANPEP-Flag
- - +
- - +
siNR3C1
- + +
- + +
180kDa
130kDa
100kDa
180kDa
130kDa
100kDa
Flag (ANPEP)
130kDa
100kDa
70kDa
130kDa
100kDa
70kDa
NR3C1
40kDa
35kDa
40kDa
35kDa
GAPDH

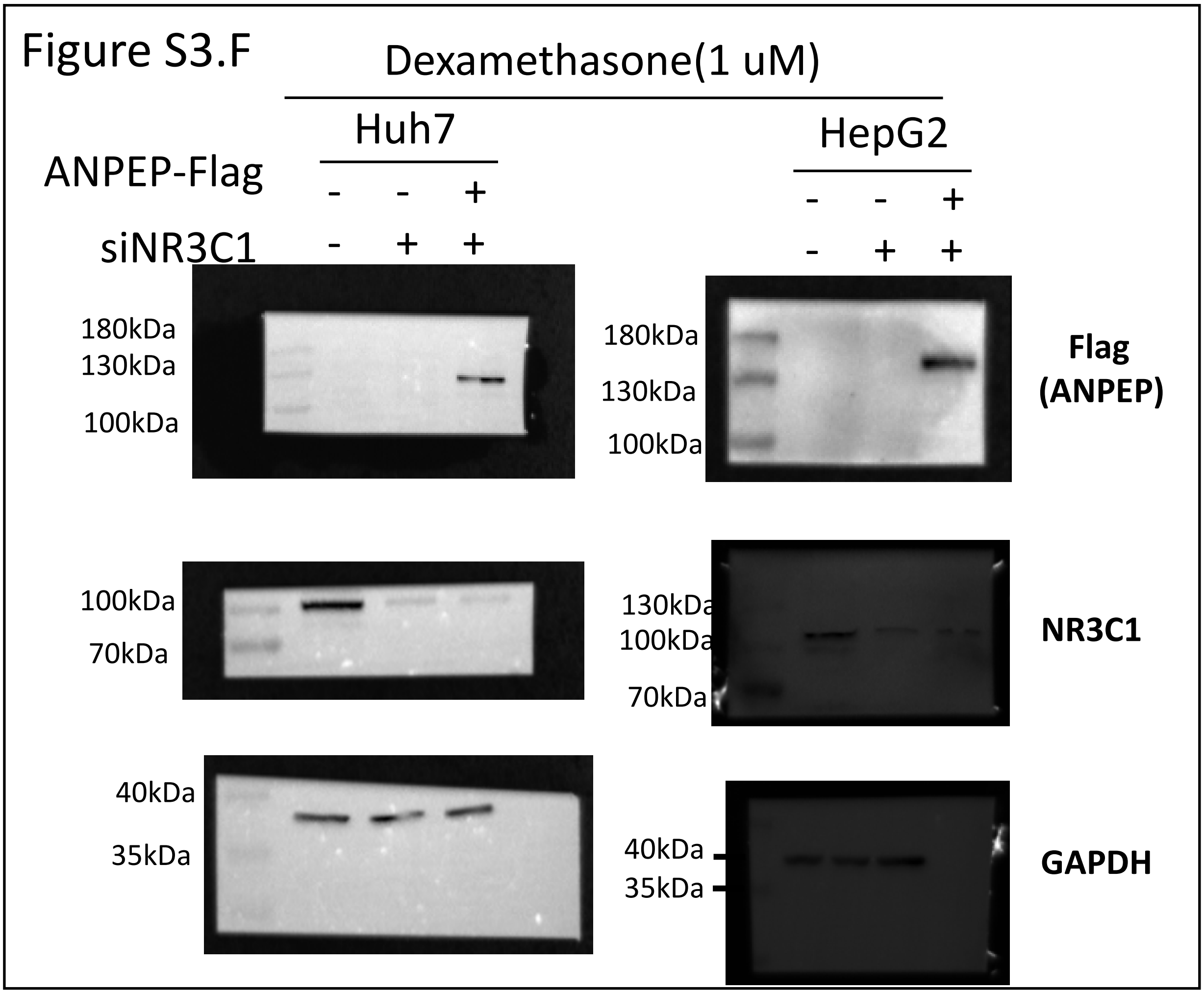
Figure S3.F
Dexamethasone(1 uM)
Huh7
HepG2
ANPEP-Flag
- - +
- - +
siNR3C1
- + +
- + +
180kDa
130kDa
100kDa
180kDa
130kDa
100kDa
Flag (ANPEP)
100kDa
70kDa
130kDa
100kDa
70kDa
NR3C1
40kDa
35kDa
40kDa
35kDa
GAPDH

Figure S5.D

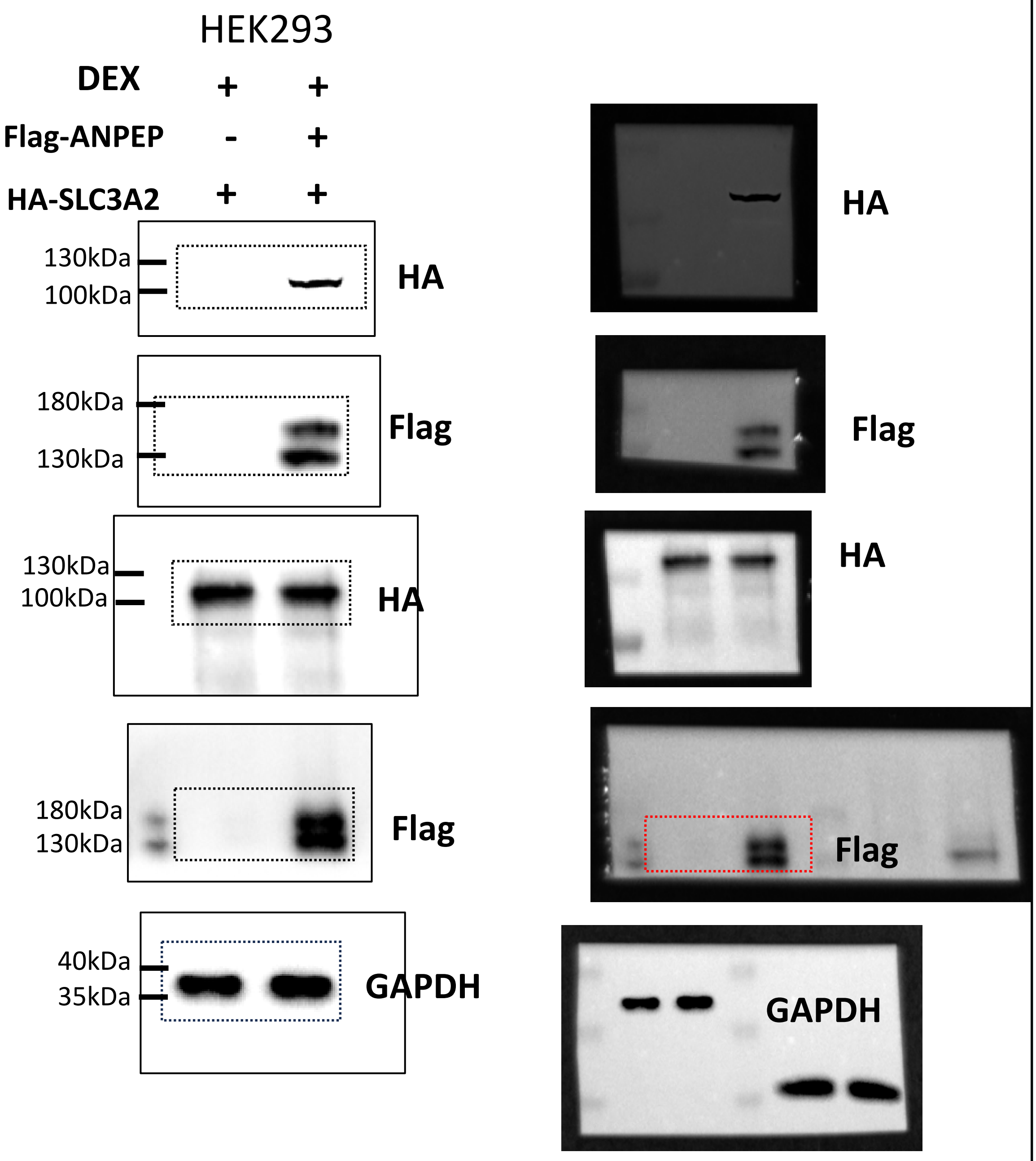

HEK293
DEX + +
Flag-ANPEP - +
HA-SLC3A2 + +
130kDa
100kDa
HA
180kDa
130kDa
Flag
130kDa
100kDa
HA
180kDa
130kDa
Flag
40kDa
35kDa
GAPDH
HA
Flag
HA
Flag
GAPDH

Figure S5.D

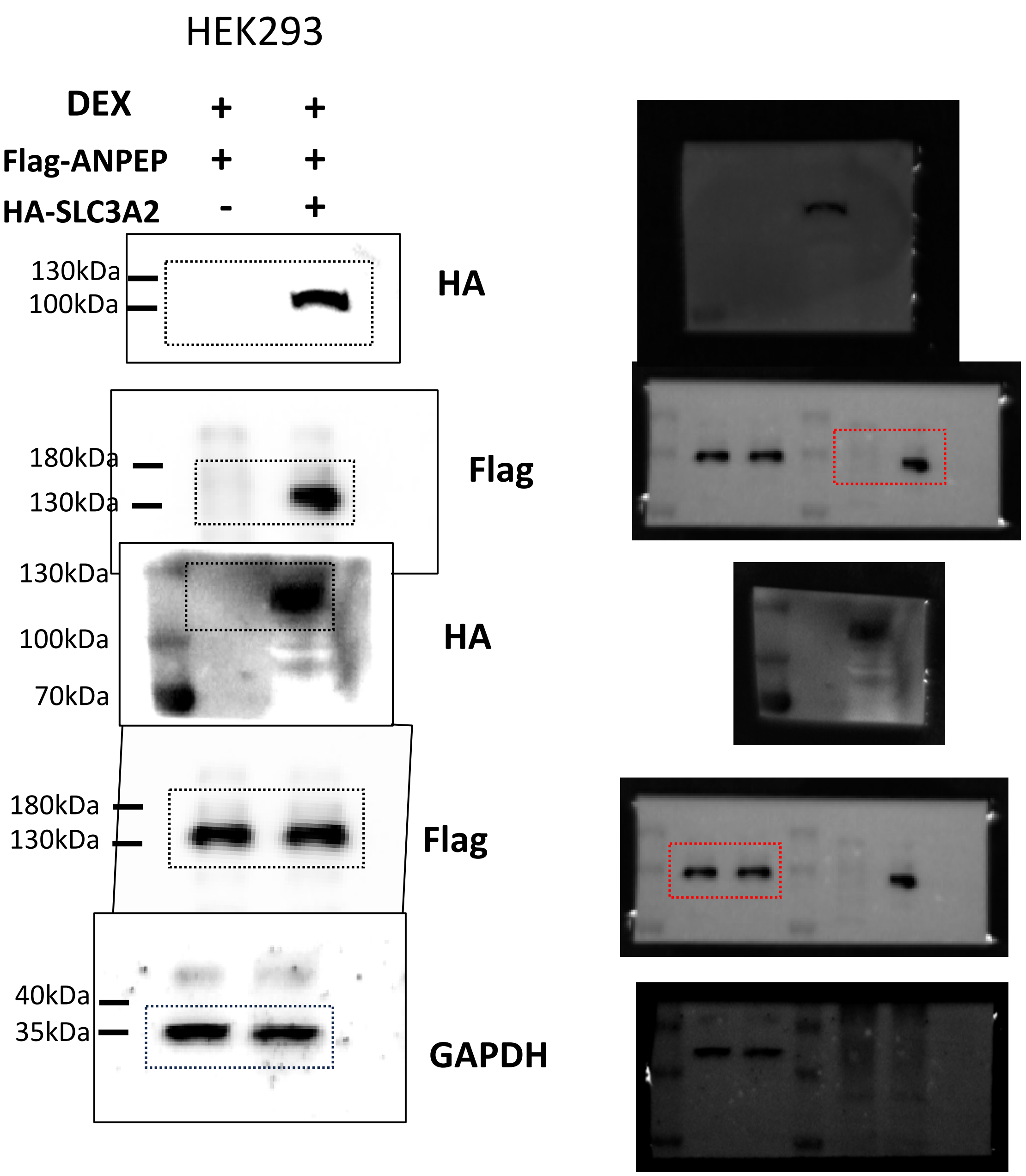

HEK293
DEX + +
Flag-ANPEP + +
HA-SLC3A2 - +
130kDa
100kDa
HA
180kDa
130kDa
Flag
130kDa
100kDa
70kDa
HA
180kDa
130kDa
Flag
40kDa
35kDa
GAPDH

Figure S5.E

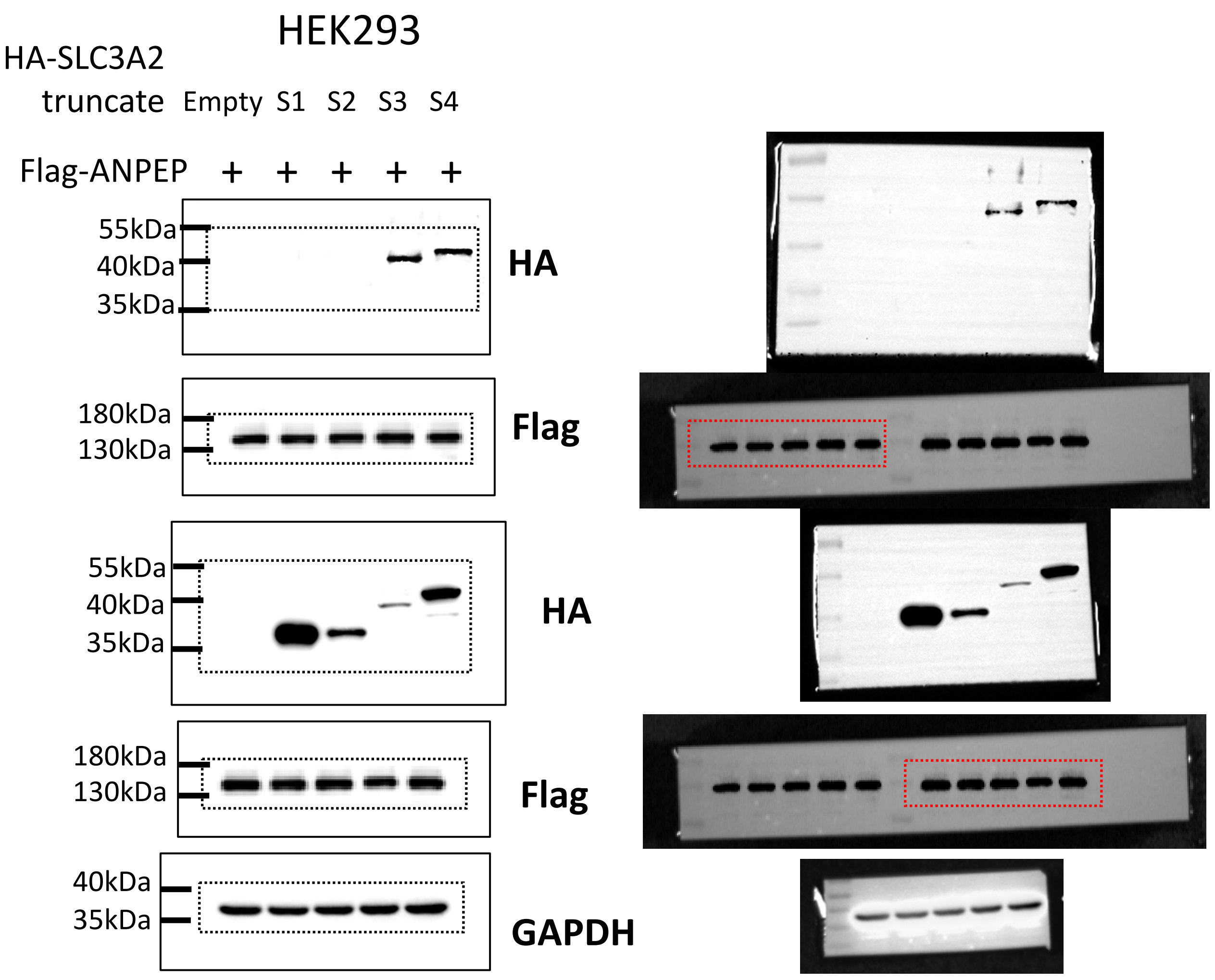
HEK293
HA-SLC3A2
truncate
Empty
S1
S2
S3
S4
Flag-ANPEP
+ + + + +
55kDa
40kDa
35kDa
HA
180kDa
130kDa
Flag
55kDa
40kDa
35kDa
HA
180kDa
130kDa
Flag
40kDa
35kDa
GAPDH

Figure S5.E

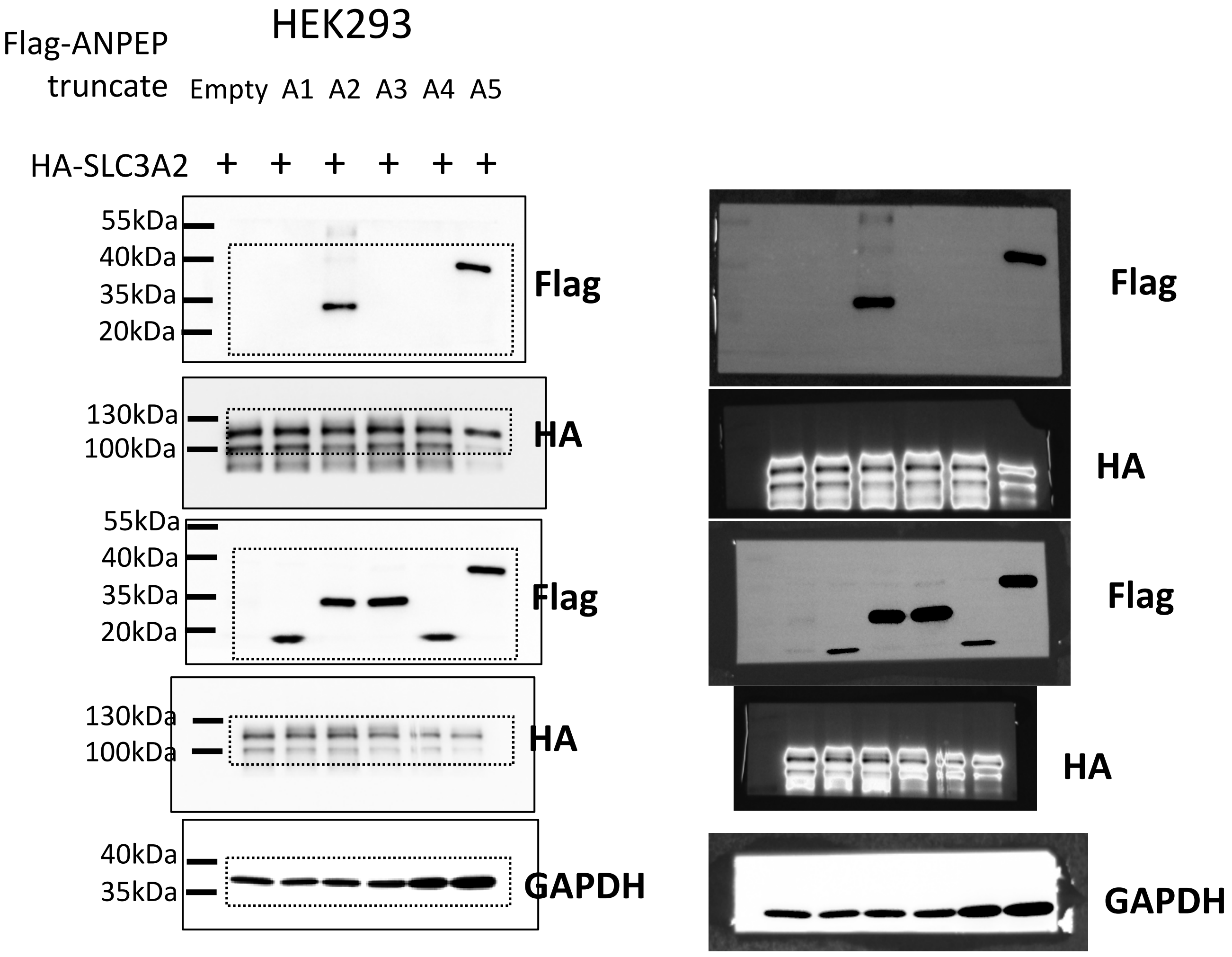
HEK293
Flag-ANPEP
truncate
Empty A1 A2 A3 A4 A5
HA-SLC3A2 + + + + + +
55kDa
40kDa
35kDa
20kDa
Flag
130kDa
100kDa
HA
55kDa
40kDa
35kDa
20kDa
Flag
130kDa
100kDa
HA
40kDa
35kDa
GAPDH
Flag
HA
Flag
HA
GAPDH

Figure S5.G

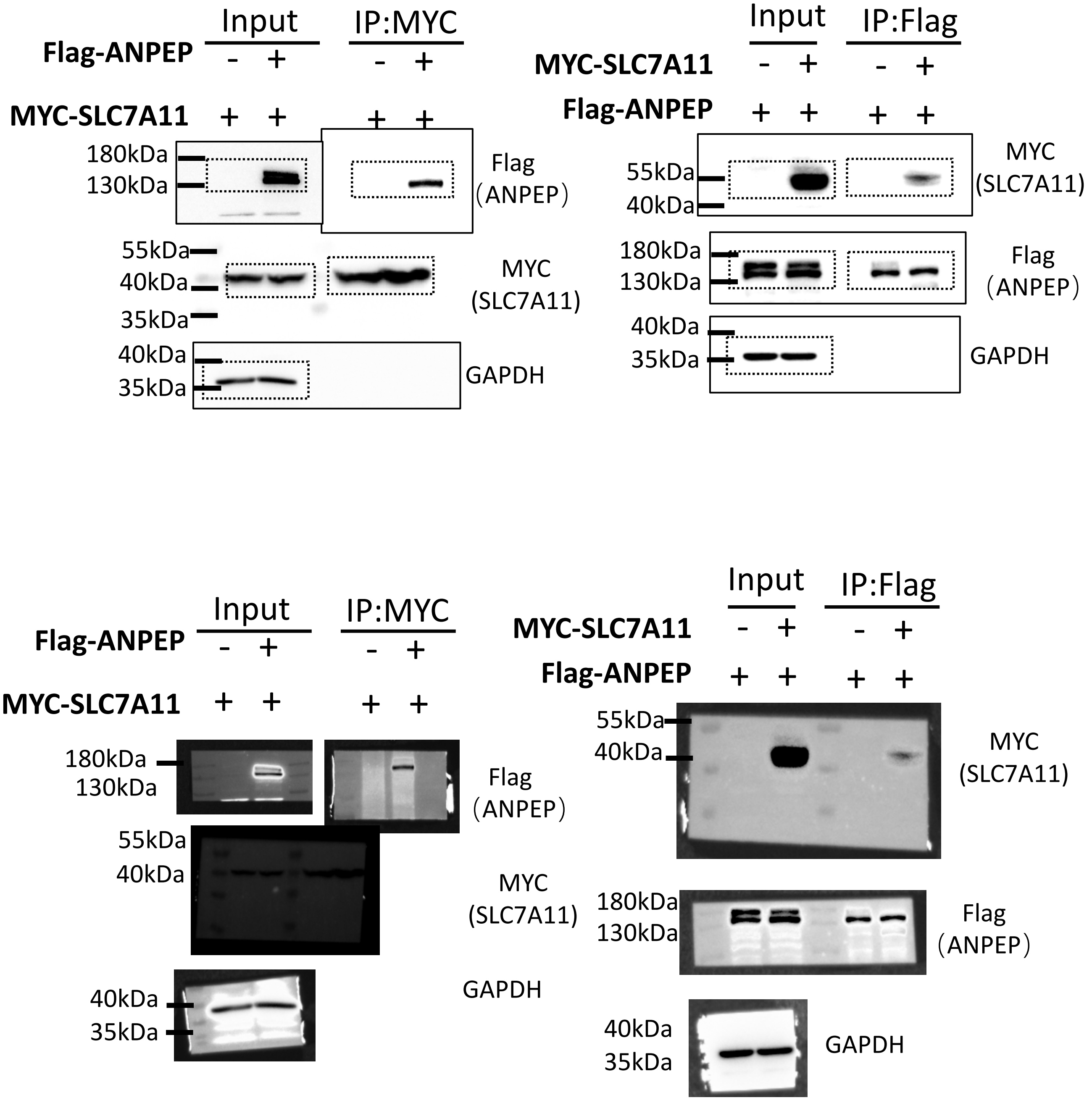
Input
IP:MYC
Flag-ANPEP
- +
- +
MYC-SLC7A11
+ +
+ +
180kDa
130kDa
Flag
(ANPEP)
55kDa
40kDa
35kDa
MYC
(SLC7A11)
40kDa
35kDa
GAPDH
Input
IP:Flag
MYC-SLC7A11
- +
- +
Flag-ANPEP
+ +
+ +
55kDa
40kDa
MYC
(SLC7A11)
180kDa
130kDa
Flag
(ANPEP)
40kDa
35kDa
GAPDH
Input
IP:MYC
Flag-ANPEP
- +
- +
MYC-SLC7A11
+ +
+ +
180kDa
130kDa
Flag
(ANPEP)
55kDa
40kDa
MYC
(SLC7A11)
40kDa
35kDa
GAPDH
Input
IP:Flag
MYC-SLC7A11
- +
- +
Flag-ANPEP
+ +
+ +
55kDa
40kDa
MYC
(SLC7A11)
180kDa
130kDa
Flag
(ANPEP)
40kDa
35kDa
GAPDH

Figure S5.H

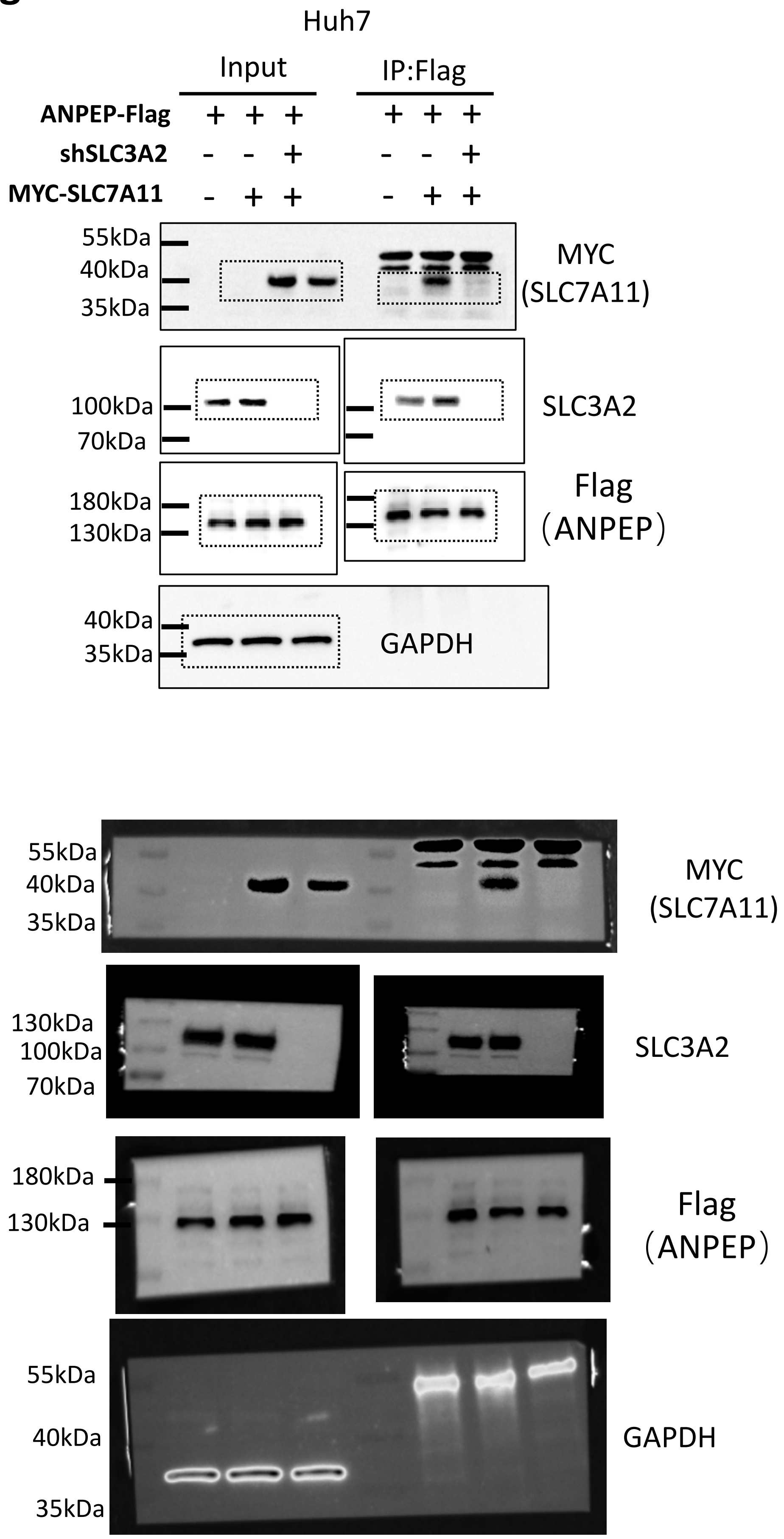
Huh7
Input
IP:Flag
ANPEP-Flag
shSLC3A2
MYC-SLC7A11
55kDa
40kDa
35kDa
MYC
(SLC7A11)
100kDa
70kDa
SLC3A2
180kDa
130kDa
Flag
(ANPEP)
GAPDH
130kDa

Figure S5.I

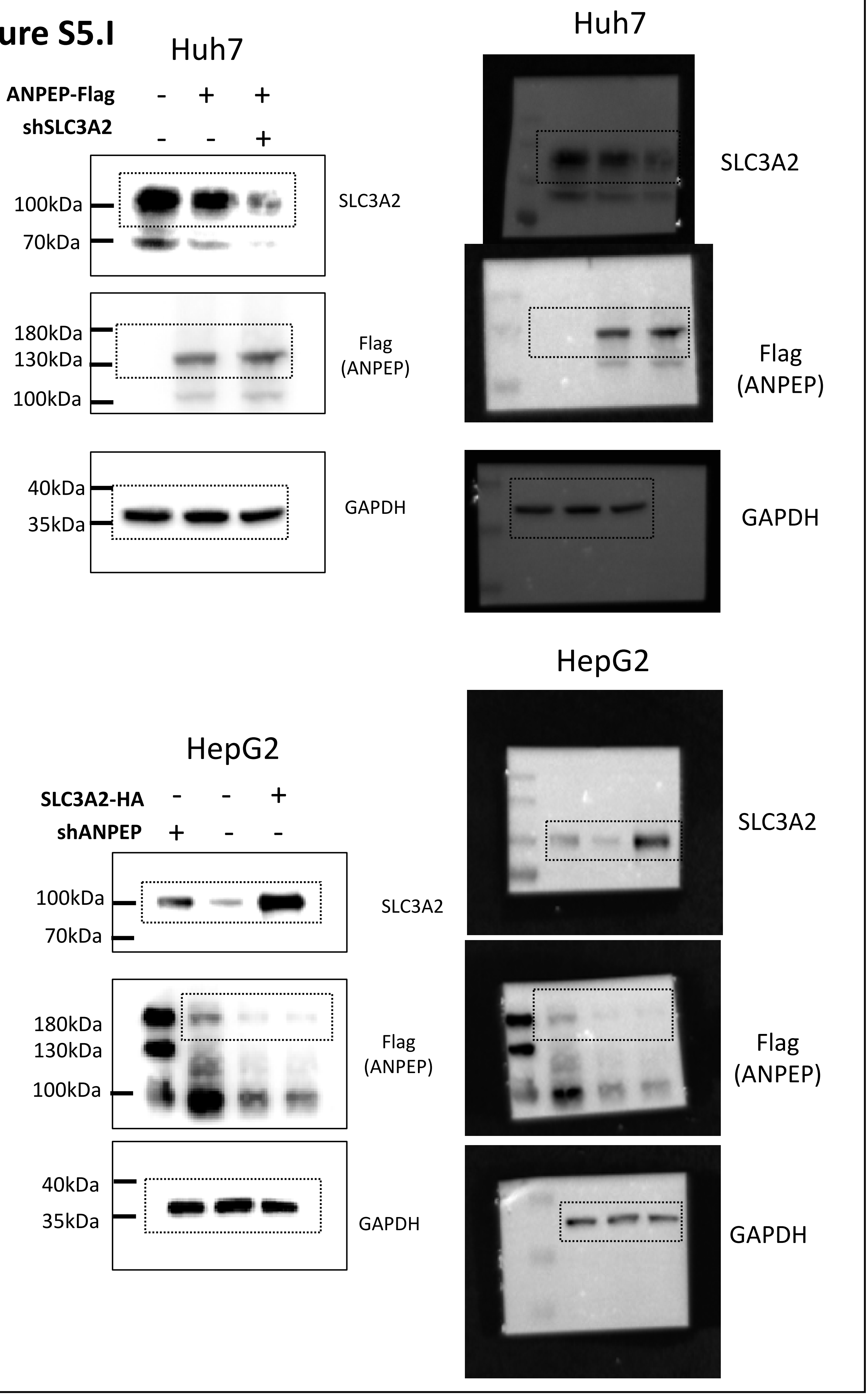

Huh7
ANPEP-Flag - + +
shSLC3A2 - - +
100kDa
70kDa
SLC3A2
180kDa
130kDa
100kDa
Flag (ANPEP)
40kDa
35kDa
GAPDH
Huh7
SLC3A2
Flag (ANPEP)
GAPDH
HepG2
SLC3A2-HA - - +
shANPEP + - -
100kDa
70kDa
SLC3A2
180kDa
130kDa
100kDa
Flag (ANPEP)
40kDa
35kDa
GAPDH
HepG2
SLC3A2
Flag (ANPEP)
GAPDH

Figure S6.A

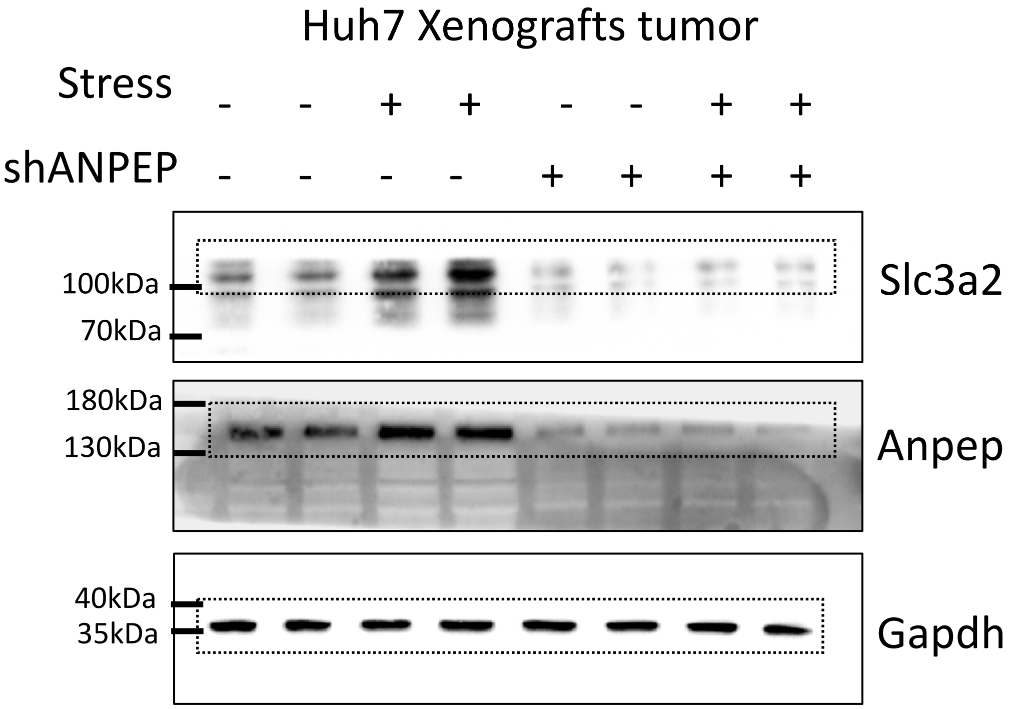

Figure S6.A

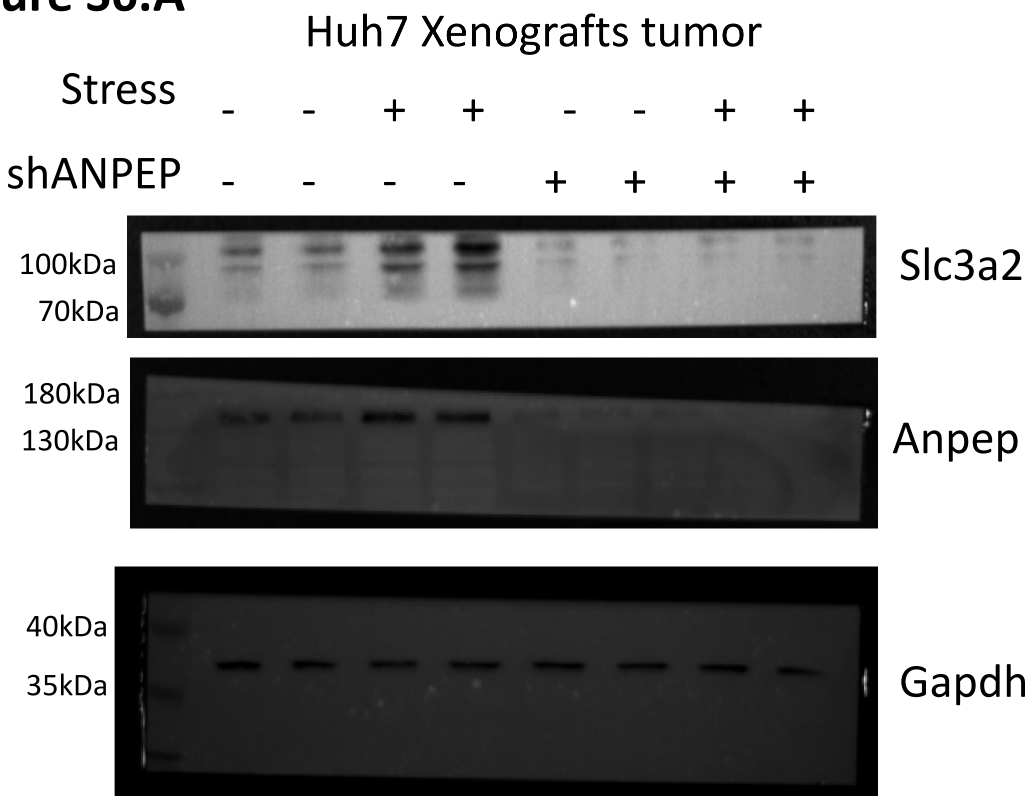

Figure S6.B

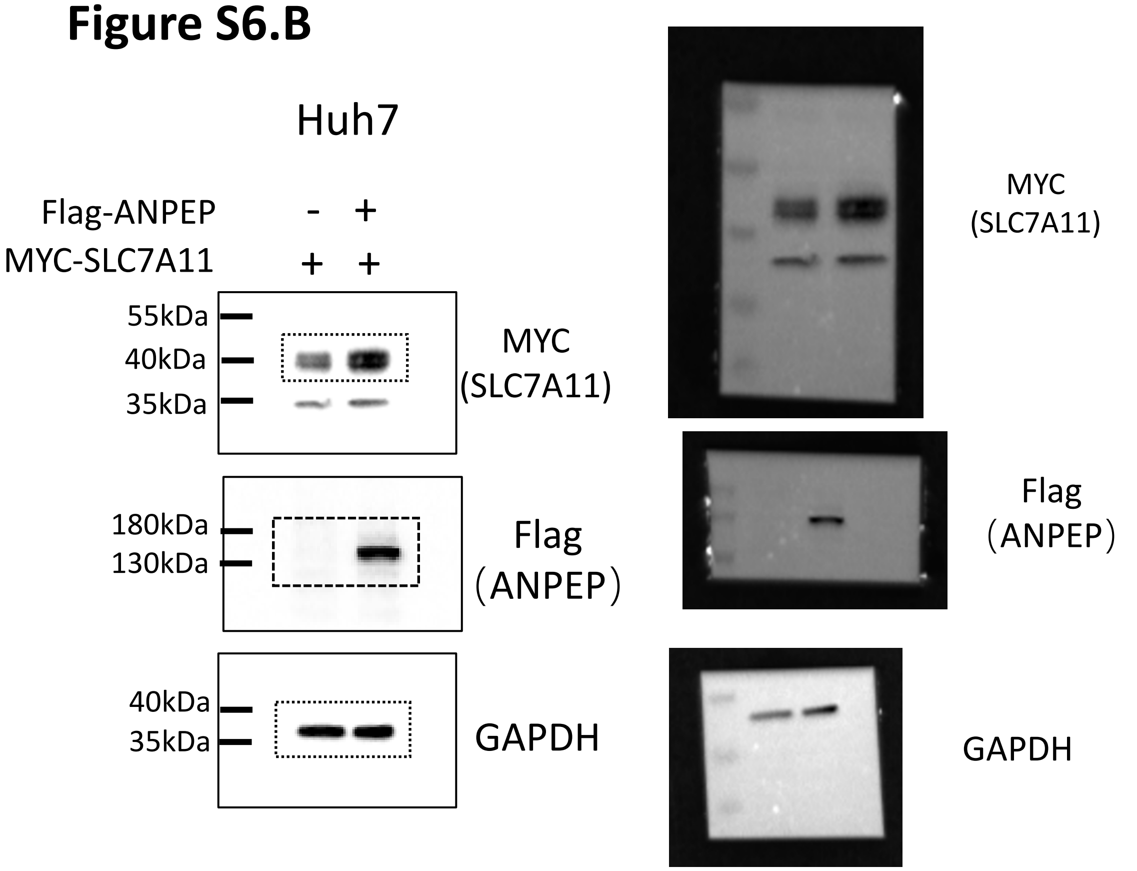

Figure S6.D

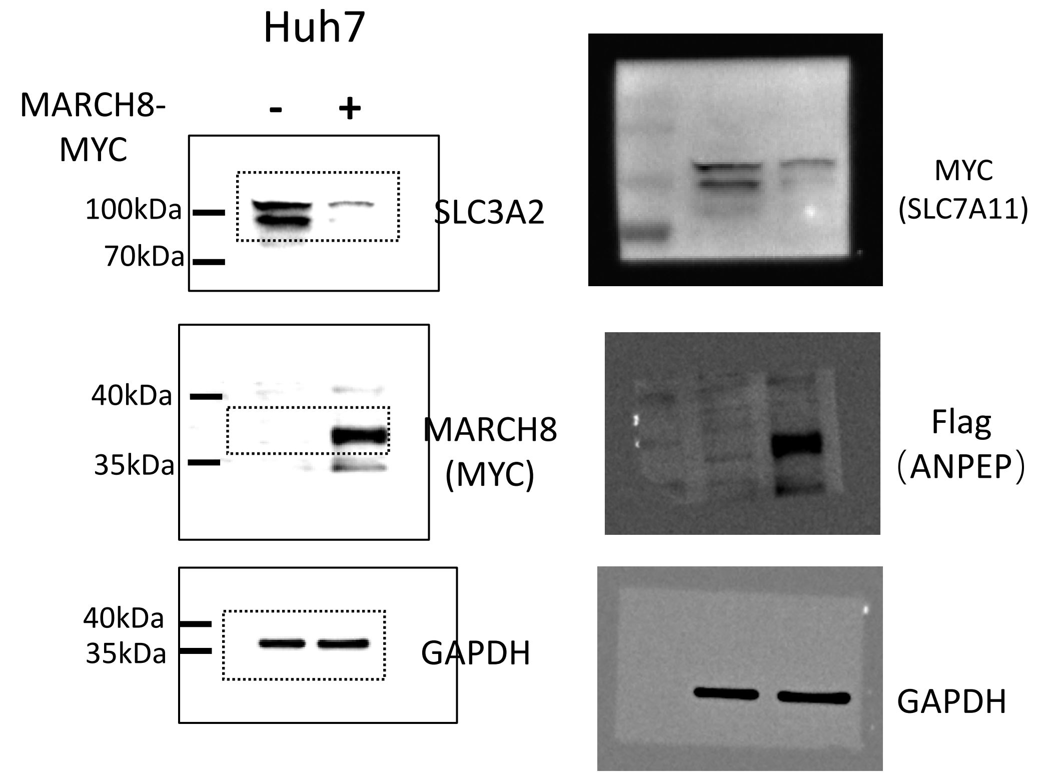

Supplement: Unedited blot and gel images [file jci-136-195685-s307.pdf]
